# Remotely-sensed, nocturnal, dew point correlates with malaria transmission in Southern Province, Zambia: a time-series study

**DOI:** 10.1186/1475-2875-13-231

**Published:** 2014-06-13

**Authors:** David Nygren, Cristina Stoyanov, Clemens Lewold, Fredrik Månsson, John Miller, Aniset Kamanga, Clive J Shiff

**Affiliations:** 1 Department of Infectious Diseases, Lund University, Malmö, Sweden; 2 Department of Molecular Microbiology and Immunology, Johns Hopkins University, Baltimore, USA; 3 PATH Malaria Control and Evaluation Partnership in Africa (MACEPA), Lusaka, Zambia; 4 Akros Global Health, Lusaka, Zambia

**Keywords:** Malaria, ARIMAX, Dew point, Remote sensing, Environment

## Abstract

**Background:**

*Plasmodium falciparum* transmission has decreased significantly in Zambia in the last decade. The malaria transmission is influenced by environmental variables. Incorporation of environmental variables in models of malaria transmission likely improves model fit and predicts probable trends in malaria disease. This work is based on the hypothesis that remotely-sensed environmental factors, including nocturnal dew point, are associated with malaria transmission and sustain foci of transmission during the low transmission season in the Southern Province of Zambia.

**Methods:**

Thirty-eight rural health centres in Southern Province, Zambia were divided into three zones based on transmission patterns. Correlations between weekly malaria cases and remotely-sensed nocturnal dew point, nocturnal land surface temperature as well as vegetation indices and rainfall were evaluated in time-series analyses from 2012 week 19 to 2013 week 36. Zonal as well as clinic-based, multivariate, autoregressive, integrated, moving average (ARIMAX) models implementing environmental variables were developed to model transmission in 2011 week 19 to 2012 week 18 and forecast transmission in 2013 week 37 to week 41.

**Results:**

During the dry, low transmission season significantly higher vegetation indices, nocturnal land surface temperature and nocturnal dew point were associated with the areas of higher transmission. Environmental variables improved ARIMAX models. Dew point and normalized differentiated vegetation index were significant predictors and improved all zonal transmission models. In the high-transmission zone, this was also seen for land surface temperature. Clinic models were improved by adding dew point and land surface temperature as well as normalized differentiated vegetation index. The mean average error of prediction for ARIMAX models ranged from 0.7 to 33.5%. Forecasts of malaria incidence were valid for three out of five rural health centres; however, with poor results at the zonal level.

**Conclusions:**

In this study, the fit of ARIMAX models improves when environmental variables are included. There is a significant association of remotely-sensed nocturnal dew point with malaria transmission. Interestingly, dew point might be one of the factors sustaining malaria transmission in areas of general aridity during the dry season.

## Background

In 2010, roughly 200 million malaria cases and 660,000 deaths related to malaria were reported worldwide and 86% of these deaths were among children under five years of age [1]. In Zambia, malaria is endemic with seasonal transmission peaking between December and May and coinciding with the rainy, wet season [2]. According to reports from the Ministry of Health (MoH) and the National Malaria Control Centre (NMCC) in Zambia, the number of malaria cases annually decreased by more than 60% between 2001 and 2008 [2]. In addition, a decrease from a parasite prevalence of 13.7% (2006) amongst children under five years of age to 8.4% (2012) has been seen in the area of the study, Southern Province, Zambia [3,4].

*Plasmodium falciparum,* the causative agent of severe malaria, is transmitted mainly by *Anopheles arabiensis* in Southern Province [5]. It is a nocturnally active mosquito with a high desiccation resistance [6]. Because of the nature and ecology of these mosquitoes, climate has a strong influence on their ability to forage and survive. Hence, temperature, humidity, rainfall, elevation, and vegetation are important determinants of malaria transmission. Development and replication of the parasite in the mosquito, the intrinsic incubation period, is affected not just by average but also by daily temperature variations [7,8]. When temperature increases, mosquitoes become infectious more rapidly [9]. Relative humidity plays an important role in mosquito survival. Recent studies have shown that low humidity shortens the lifespan of mosquitoes thereby influencing malaria transmission dynamics [10]. Rainfall is also key to ensure mosquito survival as water bodies are necessary for breeding sites [7]. Finally, vegetation, which also is reliant on moisture, impacts mosquito survival by acting as resting place and as source of food [11]. The role of moisture in the air is an important feature that regulates the flight conditions of potentially foraging mosquitoes and this has been shown in numerous studies where moisture has been defined as rainfall [12], relative humidity [13] or dew point [14]. However, humidity derived from meteorological measurements represents a broad scope of conditions and thus it does not reflect the instantaneous and very focal behaviour of mosquitoes. At critical times during dry seasons, which cover months in time, humidity must relate more to the insect’s refugia than the broad seasonal situation. Perusal of the literature in general suggests that this is the first time that a condition related to the dew point or moisture in the column of air at or near ground level has been obtained from satellite data reflecting discrete and focal ground conditions and is used for predicting malaria transmission foci. Furthermore, as these data are available nocturnally they relate directly to the environmental conditions affecting the potential flight and feeding behaviour of the vector species. Taken together, the connection between malarial transmission and environmental factors is evident across the spectrum of parasite and mosquito development, feeding and survival.

In both space and time, malaria transmission is focal and heterogeneous [15]. In Southern Zambia, during the dry season, malaria transmission is restricted a result of excessive heat and arid conditions. However when conditions ameliorate, restricted foci of transmission do occur and are likely to be the source of malaria parasites that spread during the subsequent peak transmission season, when the rains set in [16]. The basis for this lies in environmental factors that periodically permit foraging behaviour of the mosquitoes during the dry season and are likely to be critical in maintaining the malaria disease in the overall population.

Remotely-sensed data on environmental variables can be accessed through the moderated resolution imaging spectroradiometer (MODIS) sensor aboard NASA’s Aqua and Terra satellites. They view the entire Earth’s surface every one to two days; Terra passes north to south across the Equator in the afternoon while Aqua passes south to north in the evening. Through remote sensing estimates of elevation, normalized differentiated vegetation index (NDVI), nocturnal dew point (DWP), nocturnal land surface temperature (LST), and rainfall can be accessed and subsequently used in modelling transmission. NDVI is an index of the amount and healthiness of vegetation and is described as arbitrary units ranging from -1 to 1 and DWP is associated with relative humidity and is seen as a surrogate factor of air moisture since DWP is an absolute measure of water vapour in the air. LST is a measurement of the nocturnal temperature close to the ground

Here, differences in environmental variables are described among three regions in the Southern Province in Zambia with distinct transmission levels. The use of environmental variables in multivariate autoregressive integrated moving average (ARIMAX) models to investigate environmental impact on malarial transmission in the study area is described. The study incorporates dew point temperature as a surrogate for air moisture in addition to the previously studied NDVI, LST and rainfall variables [17-19]. This study could lead to identification of the remotely-sensed DWP as a significant determinant of transmission.

## Methods

### Study area, population and vectors

**Figure 1:**
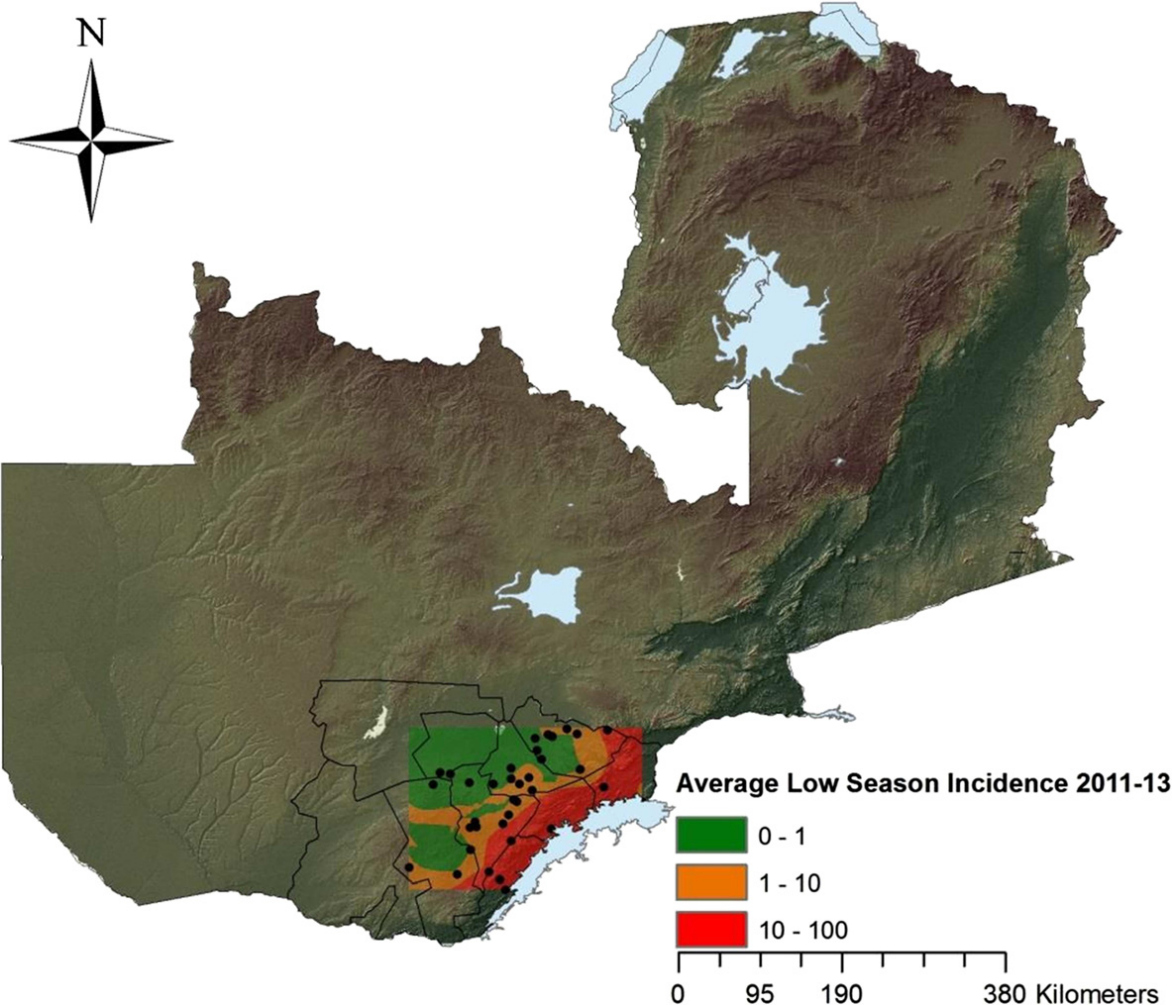
**Map of Zambia.** Showing district boundaries of Southern Province. The borders of the colour pattern in Southern Province represent the study area, with the dots representing each of the 38 health centres involved in the study. The coloured incidence map further represent the averages of weekly incidence during 2011-2013 for the dry, low season (weeks 19-48) created by using Kriging. Lake Kariba is seen east of the study area.

Southern Province, Zambia was chosen as the study area and 38 rural health centres (RHC) were included, see Figure 1*.* Coordinates of RHCs were acquired through NMCC and MoH, or when missing through field visits to clinics if possible. Population catchment estimates were obtained from the NMCC, as well as estimated yearly population growth numbers. These were cross-referenced with head counts from a sample of the clinics. Southern Province has an area of 85,283 sq. km, mainly covered by grasslands (68%), forests (12%) and savanna (12%) [20]. It is a drought-prone region with the least amount of rain compared to the rest of the country [3]. Its 2012 population is estimated at 1,589,926 out of which 75% live in rural areas and 14.4% are below five years of age [21]. The studied RHCs serve approximately 330,000 people of which the great majority is of the Tonga tribe.

### Case data

The number of weekly malaria cases confirmed by rapid diagnostic test (RDT) or microscopy was obtained from the NMCC weekly rapid reporting system database collected and housed by District Health Information System 2 software for each RHC [22]. This database is built on a surveillance model that uses mobile phones to report passively detected malaria cases at each RHC every week. This system was established to support malaria elimination efforts in areas of the country with low malaria disease burden or otherwise targeted for elimination. The study period incorporates 126 weeks and runs from 2011 week 19 until 2013 week 41. The dry, low season runs from week 19-48 and the wet, high season from week 49-18. RHCs were included only if they had available spatial coordinates, population estimates and 100% completeness rate in weekly reports through 2013 week 33. However, out of the total of 38 RHCs only 19 started reporting during the dry, low season of 2011. The remaining 19 RHCs started reporting between 2011 week 48 and 2012 week 19. These time periods marked the roll-out of the weekly reporting across the province during 2011. In total, 3,581 weekly reports with 53,203 malaria cases were analysed. The number of weekly reports with zero cases reported was 1,485. Diagnostic tests were available for the majority (92.1%) of weekly reports. Incidence data was calculated for each RHC using weekly case load and estimates of the population served.

### Buffer zones and transmission zones

Five-km buffer zones surrounding each RHC were created in an effort to account for their catchment area as well as for mosquito dispersal [7,23,24]. Buffer zone averages were then used when analysing environmental variables, except for dew point where raw data are of a lower resolution (5×5 km). Kariba and Siavonga RHCs were situated at 300 m distance from each other, which is why these were combined. The 38 RHCs were grouped into three separate transmission zones based on the average incidence levels during the dry, low transmission season, defined as week 19 to week 48. The zones were divided as follows: high transmission when incidence greater than ten cases/10,000 people per week, medium transmission when incidence of one to ten cases/10,000 people per week and low transmission when incidence of less than one case/10,000 people per week during a low season 2011-2013. There were nine, 15 and 14 RHCs included in the high, medium and low transmission zones, respectively.

### Remotely-sensed data

Remotely-sensed environmental data that have been used previously to predict or describe malarial transmission were chosen in our analysis. This included NDVI, LST and rainfall. Moreover, remotely-sensed DWP was added and elevation data obtained. With the exception of rainfall measurements that were provided by Meteosat by TAMSAT research group [25-28], data were collected by the MODIS-sensor aboard the NASA satellites Aqua and Terra [29,30]. Zonal environmental differences were analysed using non-parametric Kruskall-Wallis tests. All environmental data were rectified into weekly values.

### MODIS products

NDVI were obtained from MOD13A-products with 1-km pixel resolution [31]. NDVI use blue, red and near-infrared reflectance to determine daily vegetation indices. Monthly averages of buffers were used in the analysis. When buffers included water surface, thus resulting in erroneous and negative vegetation indices, raster data were converted to point data. This occurred at one RHC. Point data were then extracted and point values less than 0 were omitted from the analysis.

DWP was obtained from the MOD07-product with 5×5 km resolution [32]. Nocturnal measurements were averaged into eight days for the analysis. Due to cloud cover, 812 (20.1%) out of 4,033 analysed eight-daily averages had missing data. Missing data were imputed with the average of preceding and following weeks.

LST was obtained from the MOD11A-product with 1-km resolution [33]. Nocturnal measurements were averaged into eight days for the analysis. Due to cloud cover, 697 (17.3%) out of 4,033 of the 5-km RHC buffers had missing data. Missing values were imputed with the 1-km pixel representing the RHC’s position. If that was also cloud-covered, the average of preceding and following weeks was imputed. DWP and LST were expressed as ˚C.

### Elevation

Elevation data were obtained through Advanced Spaceborne Thermal Emission and Reflection Radiometer (ASTER), with a sensor with 30-m resolution, on board the Terra satellite used for creating a global digital elevation map [34].

### Rainfall

TAMSAT mean Tropical Applications of Meteorology using SATellite data and ground-based observations. This is an estimation of rainfall derived from thermal infrared channels on Meteosat, which is calibrated against ground-based rain gauge data. Monthly rainfall amounts were used in the analysis [25-28].

### Kriging

An interpolated incidence map was created to visualize malarial transmission in the region. The method of Kriging was used for this purpose and in short, it interpolates incidence data as an incidence map based on point values [35].

### ARIMAX modelling

ARIMAX models were chosen to fit the data because it accurately reflects the impact of short time fluctuations in time-series data. Models were fitted for the high, medium and low transmission zone with one model each. Five RHCs in the high-transmission zone were also independently modelled due to them having data from all seasons in the study period. These were Sinafala, Siatwinda, Siamuleya, Siavonga and Maamba RHCs. The ARIMAX(*p,d,q)* models were fitted with three categories of parameters: the autoregressive parameter (*p*), differencing steps (*d*) and the moving average parameter (*q*).

Separately for different modelled areas, association between malaria incidence and each temporally referenced environmental variable was evaluated. To determine at which lag time each particular variable was the most influential on malaria incidence, all correlations between one to 11 weeks of lag were examined. This procedure was performed for all models in the same way. Strongly correlated lag times (p-values ≤0.1) were chosen for further evaluation and were included in multiple variable ARIMAX models.

Log-transformed weekly incidence data from 2012 week 19 to 2013 week 36 were used as a *training* set for the ARIMAX model. The best possible combination of environmental covariates was determined by Akaike's information criterion (AIC) to find the best fit [36]. Model fit was then again assessed based on its AIC-value after adjustment of the model parameters. The accuracy of the model was tested on data for the *testing* period 2011w19 to 2012w18 and here evaluated with the mean average error (MAE) expressed as a percentage describing the mean of the weekly deviation between each actual measured and each predicted value [18]. The Ljung-Box Q statistic was calculated to determine if there were autocorrelations in the residuals. If statistically significant autocorrelation was found, the model was dropped [19]. A total of 417 models combining different environmental variables and ARIMAX parameters were evaluated before settling for the eight best models. See Additional file 1 for a log file describing modelling.

All models were analysed for forecasting ability. The choice was made to forecast four weeks forward starting 2013 week 37. Actual *versus* predicted incidence rate was calculated for these four weeks of forecast, and based on these values, MAE was calculated to evaluate how accurate forecasts were. MAE-values of ± 15% were rendered acceptable.

### Software

ArcGIS 9.1 and 10.2 ESRI, Redlands, California, USA was used while performing spatial statistics and further data management was performed in Microsoft Excel 2010, Microsoft. Redmond*,* Washington, USA*.* Kruskall-Wallis tests, Ljung-Box Q statistics, time-series analysis and ARIMAX modelling were performed in STATA 12.0, StataCorp, College Station, Texas, USA.

### Ethical considerations

Ethical approval was not sought for this study since aggregated, not individual, case data were used.

## Results

### Transmission and environment

**Figure 2:**
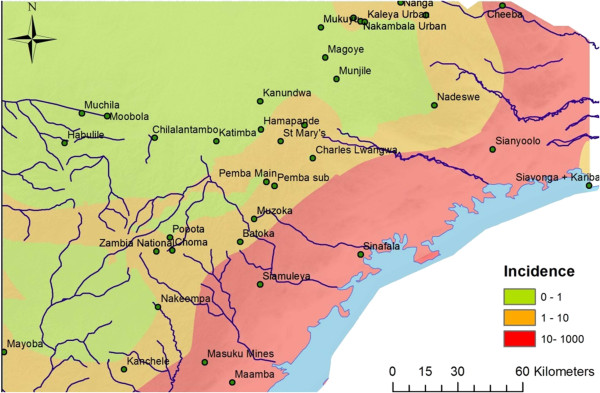
**Study area – low season incidence map.** Showing a Kriging interpolation of rural health centre (RHC) incidence data from the dry, low seasons of 2011-2013. The different colours indicate the transmission pattern at the 38 RHCs in Southern Province, Zambia.

**Figure 3:**
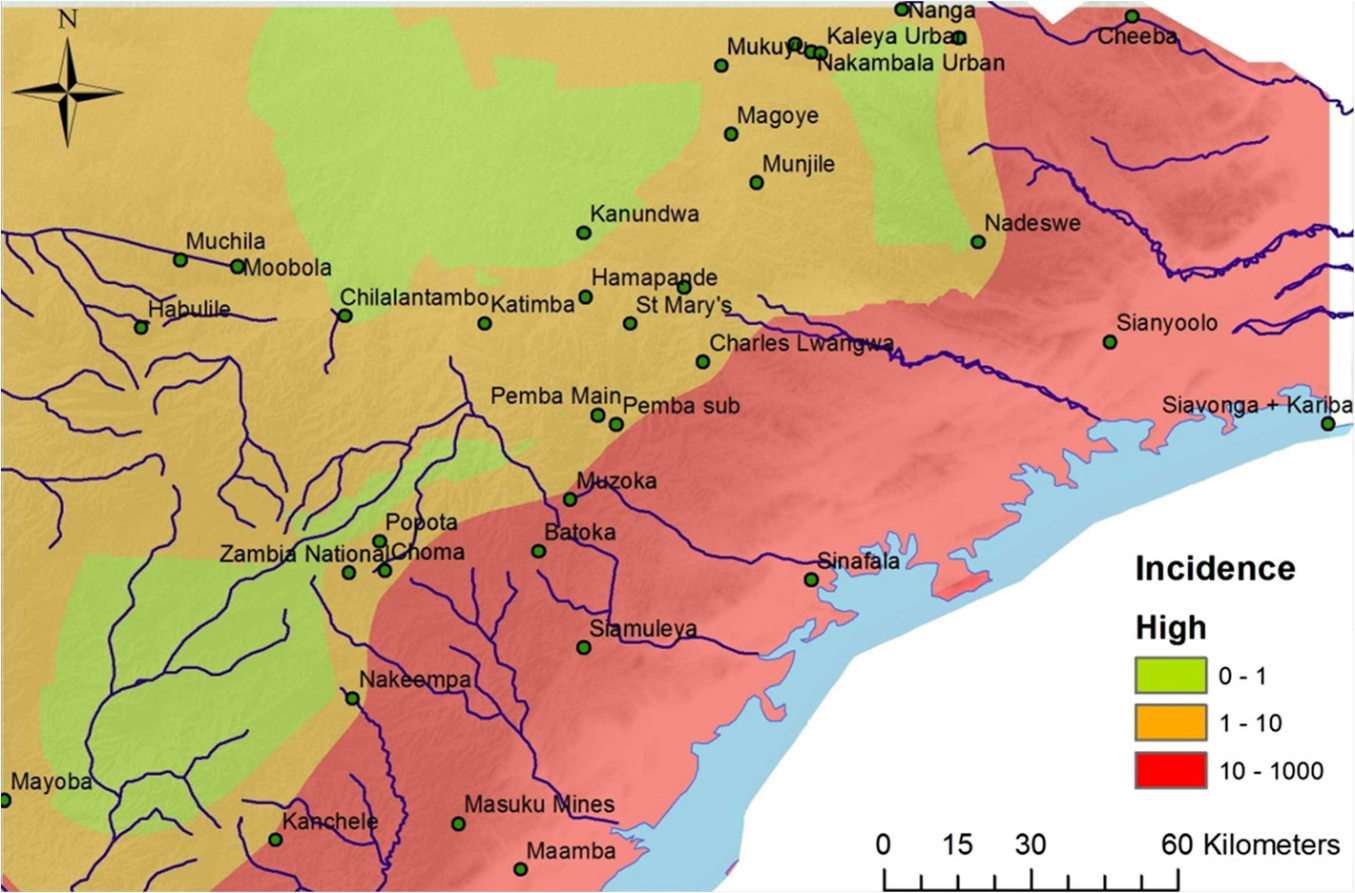
**Study area– high season incidence map.** Showing a Kriging interpolation of rural health centre (RHC) incidence data from the wet, high seasons of 2011-2013. The different colours indicate the transmission pattern at the 38 RHCs in Southern Province, Zambia.

Taken together, the average weekly incidence rates for all dry, low season weeks 2011-2013 were 0.61, 2.66 and 30.95 weekly cases/10,000 people for the low-, medium- and high-transmission zones, respectively. Between high and low season of 2011-2012 and 2012-2013, there was a downward trend in incidence with an average decrease of 32.1% for the entire study area (Tables 1, 2, Additional file 2). Kriging estimated incidence maps on both low and high season illustrating transmission patterns between seasons and zones are seen in Figures 2, 3.

**Table 1:** Rural health centre and zonal seasonal incidence

**ZONE**	**RHC**	**Pop. (2012)**	**Incidence low season 11**	**Incidence high season 11-12**	**Incidence low season 12**	**Incidence high season 12-13**	**Incidence low 13**
**HIGH**	Sinafala	6,441	47.37	94.22	32.24	122.54	56.17
	Masuku Mines	5,130	6.82	86.38	28.14	57.76	3.19
	Siamuleya	14,523	16.43	154.29	38.65	121.17	32.06
	Cheeba	2,319		59.30	12.79	69.66	13.50
	Siavonga and Kariba	18,119	9.74	14.82	10.51	9.20	6.73
	Sianyolo	12,942		47.00	13.55	51.47	12.84
	Maamba	14,003	89.70	187.58	45.23	69.08	20.13
	Siatwinda	9,691	75.39	133.24	82.72	67.33	21.88
	**Zonal pop. and mean**	**83,168**	**40.91**	**97.10**	**32.98**	**71.03**	**20.81**
**MEDIUM**	Batoka	15,115	4.04	34.88	8.18	24.38	4.08
	Choma	2,636	4.03	8.49	2.78	3.38	2.07
	Muzoka	5,082	1.37	14.76	3.28	9.66	3.00
	Pemba Main	7,622	0.42	2.03	1.49	5.04	1.43
	Pemba Sub	2,636	2.24	11.91	3.16	2.03	1.45
	Zambia National Health Services	2,721	2.64	3.68	4.41	5.92	3.39
	Kanchele	11,200	2.81	14.35	2.05	7.09	1.80
	Mayoba	2,933	4.47	5.45	2.05	2.80	0.72
	Nakambala Urban	36,711		2.84	1.21	2.04	1.09
	Nanga	9,912		1.89	1.45	1.97	2.34
	Research Station	6,127		0.93	1.31	2.53	1.32
	Nega Nega	4,904		11.65	1.36	7.21	2.20
	St Mary's	3,058			2.83	3.95	2.32
	ZCA Railways	5,524			2.53	3.65	2.07
	**Zonal pop. and mean**	**116,181**	**2.76**	**9.41**	**2.72**	**5.83**	**2.09**
**LOW**	Chilalantambo	2,636	0.39	1.04	0.25	0.51	0.62
	Nakeempa	7,622	0.28	4.31	0.92	2.11	1.00
	Popota	5,129	1.69	2.67	0.52	2.00	0.64
	Habulile	8,399	0.00	14.07	0.36	1.81	0.31
	Kaleya Urban	10,096		1.37	0.99	2.59	0.80
	Magoye	16,628		3.17	0.62	1.25	0.68
	Mukuyu	6,315		0.88	0.16	0.56	0.09
	Munjile	4,868		1.62	0.21	1.45	0.22
	Nadeswe	9,708			0.58	0.68	0.28
	Charles Lwangwa	2,405			0.83	3.33	0.45
	Hamapande	8,418			0.71	1.75	0.65
	Kanundwa	9,621			0.17	0.56	0.40
	Katimba	10,436			0.63	2.08	1.25
	Moobola	13,290		3.15	0.50	1.07	0.37
	Muchila	16,798		2.35	0.97	1.62	0.36
	**Zonal pop. and mean**	**132,369**	**0.59**	**3.46**	**0.56**	**1.56**	**0.54**

All 38 rural health centres (RHC) divided into three zones based on their weekly incidence per 10,000 individuals during the dry, low season. First column present population numbers in 2012 for each RHC as well as total population of zone. Then average of weekly seasonal incidence level for each RHC as well as zonal average is presented. Where missing values for Low Season 11 and High Season 11-12 these RHC had not yet started reporting weekly case data.

**Table 2:** Zonal and seasonal average incidence with confidence intervals

**Weekly seasonal average incidence/10,000 people**	**High-transmission**	**Medium-transmission**	**Low-transmission**
	**Zone**	**Zone**	**Zone**
2011 May-Nov	40.07 (33.90 -46,23)	3.17 (2.04-4.30)	0.74 (0.31-1.17)
2011 Dec-2012 Apr	106.95 (85.32-128.57)	10.34 (8.11-12.58)	3.48 (1.87-5.08)
2012 May-Nov	32.98 (22.92-43.04)	2.72 (1.67-3.77)	0.56 (0.34-0.78)
2012 Dec-2013 Apr	71.03 (56.96-85.09)	5.83 (4.73-6.94)	1.56 (1.02-2.11)
2013 May-Aug	19.80 (14.26-25.33)	2.08 (1.12-3.04)	0.54 (0.15-0.93)

Weekly seasonal average incidence values shown with 95% Confidence Intervals highlight the significant differences between the zones. These weekly values are averaged into seasonal values for high, medium and low-transmission zones.

In the zone of higher malaria transmission, NDVI, LST and DWP had higher measurements than in the lower transmission zone (Table 3, Additional file 2). This difference was accentuated during the low season. When comparing climatic variables between the high-, medium- and low-transmission zones, using Kruskall-Wallis test, there were significant differences seen between all three zones regarding LST (p < 0.001), DWP (p < 0.001) and NDVI (p < 0.001) during the dry, low season, with higher values for areas of higher transmission. The high-transmission zone was significantly lower elevated than the low- and medium-transmission zones (p = 0.035). No significant difference in rainfall measurements was found between all three zones during the months of lower transmission (p = 0.98).

**Table 3:** Zonal environmental differences during high and low season

**Months May-Nov**	**High zone**	**Medium zone**	**Low zone**	**Kruskall-Wallis test**
Av. NDVI	0.39	0.38	0.35	**p < 0.001**
Av. EVI	0.21	0.22	0.19	**p < 0.001**
Av. Noct. LST	17.15	12.78	13.04	**p < 0.001**
Av. Noct. DWP	1.17	-1.42	-1.51	**p < 0.001**
Rainfall mm	8.56	9.07	9.26	p = 0.98
**Incidence per 10000**	30.95	2.66	0.61	
**Months Dec-April**	**High zone**	**Medium zone**	**Low zone**	**Kruskall-Wallis test**
Av. NDVI	0.63	0.63	0.60	**p = 0.019**
Av. EVI	0.42	0.42	0.40	p = 0.072
Av. Noct. LST	20.32	16.79	16.98	**p < 0.001**
Av. Noct. DWP	9.42	7.83	7.81	**p < 0.001**
Rainfall mm	121.24	125.45	123.74	p = 0.74
**Incidence per 10,000**	87.89	8.05	2.49	
**Mean elevation**	785	1176	1134	**p = 0.035**

Normalized differentiated vegetation index (NDVI), enhanced vegetation index (EVI), nocturnal land-surface temperature (LST) and nocturnal dew point (DWP), rainfall, and incidence are shown for the three different zones. These are averaged seasonal values of the dry, low season (May-Nov) and for the wet, high season (Dec-April). A seasonal average of weekly incidence calculated per 10,000 people and week as well as mean elevation of the different zones are also shown. All study years (2011-2013) are used for the averaged values for both environmental variables and incidence. Kruskall-Wallis Test p-values are shown for whether there are significant differences between all three groups.

During the wet, high season the environmental differences decreased between the three zones while transmission as well as the environmental variables increased throughout the study area. However, significant differences between all three zones were still seen for LST (p < 0.001) as well as DWP (p < 0.001) and NDVI (p = 0.019). There were no significant differences between all three zones regarding rainfall (p = 0.74).

### ARIMAX models

**Figure 4:**
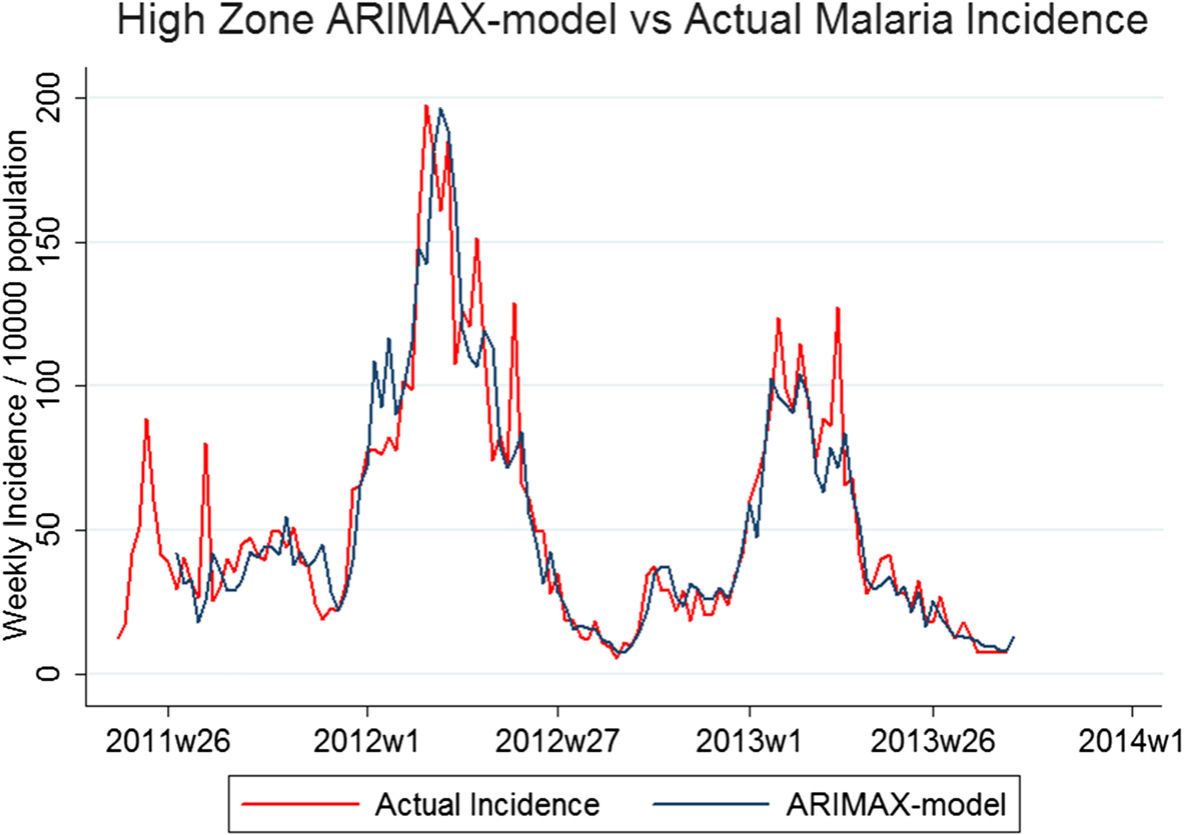
**ARIMAX model in high-transmission zone *vs. *actual incidence.** Graphs depicting two lines: actual malaria incidence *vs.* ARIMAX model prediction of incidence for the high-transmission zone, the best fit of the three zonal models. The testing period is 2011 w19 to 2012 w18 and the fitting period is 2012 w19 to 2013w36.

**Figure 5:**
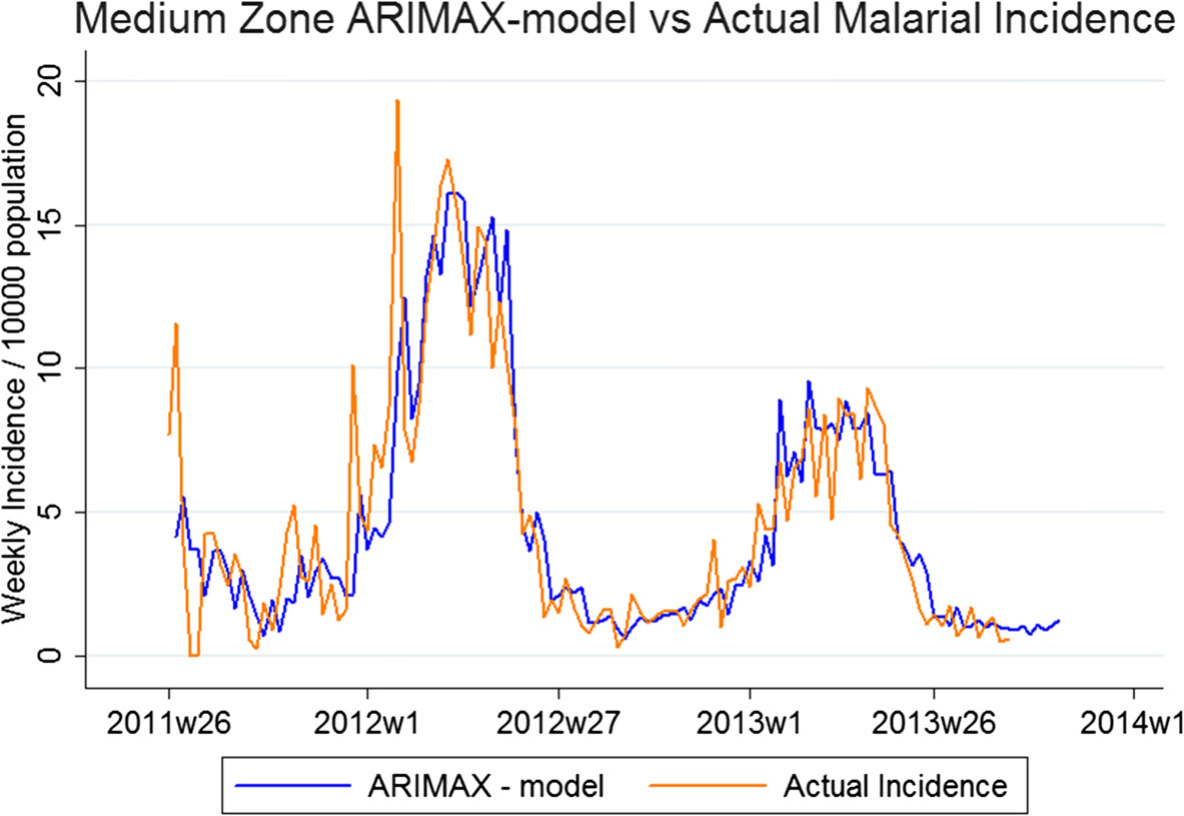
**ARIMAX model in medium-transmission zone *vs. *actual incidence.** Graphs depicting two lines: actual malaria incidence *vs.* ARIMAX model prediction of incidence for the medium-transmission zone, the second best fit of the three zonal models. The testing period is 2011 w19 to 2012 w18 and the fitting period is 2012 w19 to 2013 w36.

**Figure 6:**
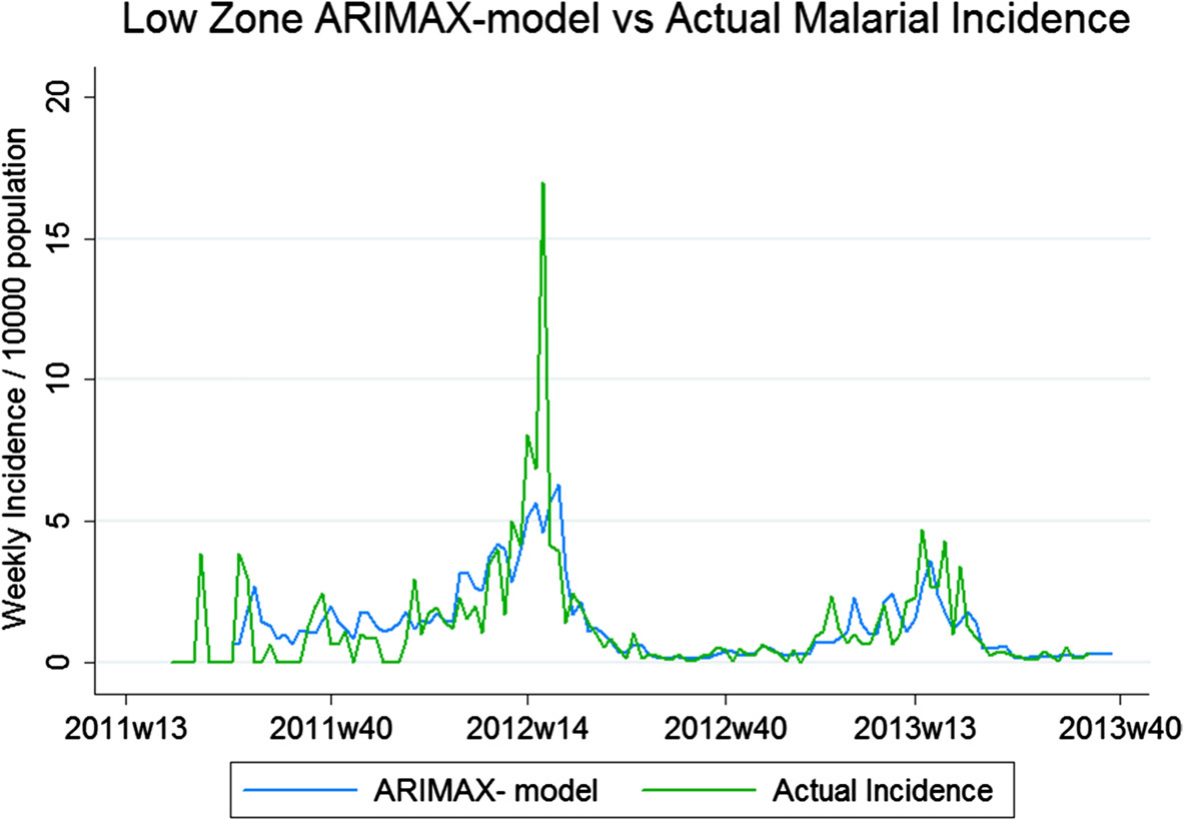
**ARIMAX model in low-transmission zone *vs. *actual incidence.** Graphs depicting two lines: actual malaria incidence *vs.* ARIMAX model prediction of incidence for the low-transmission zone, the worst fit of the three zonal models. The testing period is 2011 w19 to 2012 w18 and the fitting period is 2012 w19 to 2013 w36.

The best fitting parameters of the three zonal models were High Zone; ARIMAX(6,1,5), Medium Zone; ARIMAX(4,0,3) and Low Zone; ARIMAX (4,0,4). The MAE for the testing period for the high, medium and low zone-models were 6.4, 16.2 and 28.6%, respectively, highlighting better model fit in areas of higher transmission. Significant predictor covariates of the high-transmission zone included DWP (p < 0.01, coefficient = 0.033), LST (p < 0.001, coefficient = 0.14) and NDVI (p < 0.01, coefficient = 1.38), all with a seven-week lag time. NDVI (p = 0.19, coefficient = 0.83) with a lag time of five weeks also improved model fit, however non-significantly. For the medium-transmission zone, significant model variables were DWP (p < 0.001, coefficient = 0.076) and NDVI (p < 0.001, coefficient = 3.51) with lag times of five and eight weeks, respectively. For the low-transmission zone, significant model variables were DWP (p < 0.001, coefficient = 0.073), NDVI (p < 0.001, coefficient = 3.79) and non-significant LST (p = 0.348, coefficient = 0.024) with lag times of five, eight and three weeks, respectively (Table 4, Figures 4, 5, 6). Taken together, adding environmental variables improved the fit for all models, and DWP and NDVI were highly significant as predictors for all three zones. LST was a highly significant predictor of the model developed for the high-transmission zone. In addition, non-significant variables, i e, LST lagged at three weeks for low zone and NDVI lagged at five weeks for high zone, were kept in the models since they improved AIC.

**Table 4:** Zonal ARIMAX models’ variables, parameters and forecasts

**Model and parameters ( *p,d,q *)**	**AIC-fit**	**MAE-test**
High-ARIMAX( 6,1,5)	13.34	6.4%
Medium-ARIMAX (4,0,3)	80.61	16.2%
Low-ARIMAX (4,0,4)	137.44	28.6%
**High zone variables**	**Coefficient**	**p-value**
L7 DWP	0.033	**p < 0.01**
L7 LST	0.14	**p < 0.001**
L5 NDVI	0.83	p = 0.19
L7 NDVI	1.38	**p < 0.01**
**Medium zone variables**	**Coefficient**	**p-value**
L5 DWP	0.076	**p < 0.001**
L8 NDVI	3.51	**p < 0.001**
**Low zone variables**	**Coefficient**	**p-value**
L3 LST	0.024	p = 0.348
L8 NDVI	3.79	**p < 0.001**
L5 DWP	*0.073*	**p < 0.001**
**Forecast w37-41 2013**	**MAE**	**Predicted *vs. *** **actual incidence**
HIGH-ARIMAX	-29.40%	63.0 *vs.* 89.9
MEDIUM-ARIMAX	40.27%	3.77 *vs.* 3.48
LOW-ARIMAX*	-29.59%	0.91 *vs.* 1.48

Akaike’s information criteria (AIC) and mean average prediction error (MAE) are shown for the best-fit models of the high-, medium- and low-transmission zone ARIMAX models. MAE is calculated for the testing period of the models, i e, 2011 w19 through 2012 w18. Normalized differentiated vegetation index (NDVI), nocturnal land-surface temperature (LST) and nocturnal dew point (DWP) are shown with specific lag-times with its coefficients as well as p-values. Four-week forecasts MAE and predicted cases *vs.* actual cases for the four weekly forecasts of the zonal models are shown.

*ARIMAX LOW forecast only three weeks ahead, due to using LST lagged at three weeks.

The five ARIMAX models of single RHCs in the high zone had, apart from previous weeks’ incidence data, significant predictors involving LST, DWP and NDVI in the best fit for these models as well as non-significant variables that improved AIC. Predictor covariates for Maamba were NDVI (p = 0.982, coefficient = 0.025) lagged at seven weeks, and for Siamuleya LST (p < 0.05, coefficient = 0.091 and p < 0.01, coefficient = 0.15) lagged at five and seven weeks, respectively. Siatwinda predictors were DWP (p < 0.001, coefficient = 0.061) lagged at six weeks, Siavonga had NDVI (p < 0.001, coefficient = 8.09) lagged at seven weeks and Sinafala had NDVI (p < 0.01, coefficient = 1.13 and p = 0.328, coefficient = 1.13) lagged at four and six weeks, respectively. These RHC models had MAE for the testing period ranging from 0.7 to 33.5% (Table 5). None of these or the zonal models assessed showed any significant serial autocorrelation in its residuals when performing Ljung-Box Q test.

**Table 5:** Rural health centre ARIMAX-models’ variables, parameters and forecasts

**Model and parameters (p,d,q)**	**AIC-fit**	**MAE-test**
Maamba (5,0,5)	154.53	33,5%
Siamuleya (2,1,3)	125.57	0,7%
Siatwinda (4,0,2)	166.26	20,7%
Siavonga (1,0,0)	161.20	14,2%
Sinafala (3,0,2)	83.10	1,54%
**Maamba variables**	**Coefficient**	**p-value**
L7 NDVI	*0.025*	p = 0.982
**Siamuleya variables**	**Coefficient**	**p-value**
L5 LST	*0.091*	**p < 0.05**
L7 LST	*0.15*	**p < 0.01**
**Siatwinda variables**	**Coefficient**	**p-value**
L6 DWP	*0.061*	**p < 0.001**
**Siavonga variables**	**Coefficient**	**p-value**
L7 NDVI	*8.09*	**p < 0.001**
**Sinafala variables**	**Coefficient**	**p-value**
L6NDVI	*1.13*	P = 0.328
L4NDVI	*3.38*	**p < 0.01**
**Forecast model w37-41**	**Forecast MAE**	**Predicted vs actual incidence**
Maamba	-37.64%	41.6 vs. 75.1
Siamuleya*	-13.13%	17.1 vs. 22.3
Siatwinda	2.34%	182.6 vs. 191.9
Siavonga	-12.11%	21.8 vs. 29.1
Sinafala	*No data*	*No data*

Five rural health centres (RHC) from the high zone were modelled. Akaike’s information criteria (AIC) and mean average error (MAE) are shown for each of the five best-fit ARIMAX models. AIC was calculated for the fitting period. MAE was calculated for the testing period, 2011 w19 through 2012 w18. Normalized differentiated vegetation index (NDVI), nocturnal land-surface temperature (LST) and nocturnal dew point (DWP) are shown with specific lag-times with its coefficients as well as p-values for each RHC. Four-week forecasts MAE and predicted cases *vs.* actual cases for the four weekly forecasts of the health centre models are shown. Sinafala had insufficient data for the forecast period to be evaluated.

*Siamuleya forecast only three weeks ahead, due to missing data thereafter.

Finally, MAE in model forecasting of four weeks ranged from -37.64 to 40.27% when comparing all models (Tables 4, 5). Generally, RHC models showed better forecasts than the zonal models with three out of five RHC models forecasting with a MAE of -13.13 to 2.34% (Table 5).

## Discussion

There are significant correlations between remotely-sensed nocturnal dew point, normalized differentiated vegetation index, land-surface temperature, and malaria transmission in Southern Province, Zambia. Interestingly, remotely-sensed dew point could be a previously unidentified factor sustaining malaria transmission during the dry season. By implementing these environmental variables in models of transmission, better forecasts of changes in trends of malaria transmission could be created. However, predicting and forecasting abilities of models varied greatly.

### Transmission and environment

In this study, the zone of high transmission sees sustained high transmission also in the dry, low season (Table 2, Figure 2, Additional file 2). Small, albeit significant, environmental differences in DWP, LST and NDVI within the Southern Province are seen (Table 3, Additional file 2) and indicate that these factors are associated with malaria transmission, as well as increased in areas of higher transmission intensity. The strong association in the models between these environmental variables and the transmission intensity during the dry season could be helpful in pointing to foci of persistent malaria parasite circulation. Theories explaining the environmental impact could postulate that humidity and vegetation are important in creating possibilities for mosquito breeding, ovipositing and foraging. Additionally, temperature affects the survival time of mosquitoes and as it is usually high, it affects the extrinsic incubation period and the gonotrophic cycle of the mosquito [7,8]. As for elevation, it is known to generally correlate negatively with transmission, which is also seen here. Furthermore, humidity and temperature possibly permit and also sustain the foraging of mosquitoes, including not only the bite-transmitting sporozoites, but also ingestion of gametocytes before the extrinsic incubation period starts. It is important to understand that all these interactions of environmental variables are linked and inevitably affect each other and this needs to be studied further.

Lag times for all different environmental variables varied from three to eight weeks in the chosen models, variation in each significantly correlating environmental variable was also seen between the different models, and varying exogenous variables have been seen in similar studies [17-19]. These different environmental correlations relating to different levels of transmission imply that it will be difficult to create models that fit in different settings due to the complex and local interaction between environment and transmission. The diverse effects of environmental variables could explain why different lag times are apparent in different areas, e.g., why a significant lag of temperature can be five weeks in one zone and seven weeks in another. The idea that DWP, LST and NDVI could be factors sustaining transmission in the dry, low season in the zone of higher transmission is strengthened by the fact that they show significant correlations with incidence as well as improve the fit of the models used in this study.

Previously rainfall has been shown to influence malarial transmission [7]. Here, a correlation between rainfall and transmission is found, however, rainfall did not improve model fit when included in models. Endogeneity could here be a factor, but interestingly, there are no significant differences in rainfall between the zones. In other words, transmission rather could be driven by other sources of humidity during the dry, low season. The hypothesis of significant association of DWP with transmission is shown in this study and should be further studied when modelling malaria. Explanations of the higher measurements of DWP in areas of higher transmission could be that possibly groundwater tables here are higher, which both could sustain the vegetation, despite lack of rainfall, and by transpiration also increase water vapour in the air. The fact that DWP can be obtained from satellite makes detection of local areas of increased aerial moisture feasible and adds an attribute to NDVI to help define dry season foci of transmission. Healthy vegetation sustains transpiration, thereby creating humidity and possibly permitting dispersal and breeding as well as foraging behaviour of mosquitoes. Possibly, it is a combination of these factors as well as a factor of proximity to water [37], exemplified by the high zone’s vicinity to the Lake Kariba (Figures 1, 2, 3), that sustains the dry season transmission in high transmission zone.

### ARIMAX models

ARIMAX models were chosen because they account for the autoregression of transmission and have been tested in malaria modelling previously [17-19]. They also provide the opportunity to use exogenous variables such as remotely-sensed, spatially and temporally referenced environmental data. These types of models are often applied to time-series data and require stationary and normally distributed data. Consequently, data can be adjusted to remove the non-stationary component by adding a differencing step. Using log-transformed data in ARIMAX models’ variance of the series can be stabilized.

The environmental variables providing the best-fit model varied between the zones and RHC. Also, the ARIMAX parameters varied. Similar regional variations in predictors have also been seen in previous studies [17-19]. For zonal models, higher transmission was associated with better fit (Figure 4). This was expected and could be due to the fact that the models involving medium and low transmission had more weeks of zero transmission, complicating log-transformation for normalization of data and fitting of models. Previously, this issue has been addressed by fitting negative binomial distributions to the data of low incidence [36]. Such mathematical alterations as well as adding seasonal parameters to the model after more years of data could be useful in optimizing future models of malarial transmission in Zambia.

When looking at the *testing* period for the five models on single RHCs, the MAE ranged from 0.7 to 33.5% (Table 5). The MAE is calculated based on the predicted cases *vs.* actual cases, thus, when predicted cases were similar to the actual reported cases for the testing period, the MAE decreases. Lack of reported diagnoses due to stock-outs probably explains the slightly worse MAE for the Siatwinda model, which had 11 weeks of stock-outs compared to two to four weeks for the others. The high MAE of the Maamba model could possibly be explained by the fact that here microscopy was used as a back-up when stock-outs of RDT occurred, thus providing changing sensitivity and specificity of the diagnoses reported.

The forecasting ability of the zonal models was average (Table 4). Possibly, this was due to the zonal forecasts covering wide areas and during times of lower transmission, i e, the dry, low season, the zonal impact from a single RHC is greater than when all RHCs in the zone exhibit higher transmission, thus making the zonal model more vulnerable to local fluctuations and aberrations in transmission.

Interestingly, the RHC-level models show acceptable four-weekly forecasts (MAE -13.13-2.34%) in three out of five RHCs modelled (Table 5). The remaining two, Sinafala and Maamba*,* respectively, had missing reports of data starting 2013 week 33 and inconsistent sensitivity due to RDT stock-outs with back-up microscopy during the four-week forecast period. This highlights the importance of maintaining sensitivity in diagnostics and continuous reports in real-time modelling. Furthermore, since the models of single RHCs show better results in forecasting, one could argue that it seems that in forecasting it is presumably of greater importance with a higher resolution, ie, RHC-level rather than zonal. Nonetheless, predictions and forecasts of all models were varying and to be implemented and tested further investigation and studies are needed.

In other words, the environmental association with malaria transmission during the dry season has been shown here. Specifically, DWP as well as previously studied NDVI and LST have been identified as determinants of transmission. Further investigation of the environmental importance during the dry season could help facilitate active screening, including mass screen and treatment as well as mass drug administration directed towards predicted foci of sustained transmission. Such administration programmes has recently been highlighted as strategically important targets [38,39]. If accessible, more sensitive diagnostics such as PCR could then be used to identify greater parts of symptomatic, but most importantly, also asymptomatic carriers during this time period.

### Limitations

This study has numerous limitations here enumerated. First, passively detected case data, population estimates as well as satellite-based environmental data have limitations in accuracy. Passively detected cases may not adequately represent transmission, as a portion of the population may be represented by asymptomatic cases or because of differences in health-seeking behaviour. These errors become more emphasized at the RHC level due to smaller study groups. Furthermore, health-seeking behaviour that could create aberrations in data will affect areas with low transmission more, possibly in part explaining why zonal models of these areas performed worse. Second, the analysis used RHCs’ coordinates as the focal point of transmission because there were no available data on household coordinates. Due to mosquito flight dispersal [7,23,24] and big catchment areas, it is therefore hard to pinpoint where the actual transmission occurred. Nonetheless, Southern Province is a rural, agricultural area with a stable population who presumably tend to attend clinics since there are no other sources of anti-malarials [40]. This was accounted for by the use of buffer zones. However, using buffer zones and their average values could introduce a spatial error in the data, which in turn could have affected results. Third, missing data had to be imputed since remotely-sensed data was missing when cloud coverage occurred. Also, remotely-sensed data needed extrapolation to fit with the weekly reports of malaria cases. Fourth, the impact of RDT stock-outs on modelling is important to apprehend since the models are shaped reliant on the incidence. Zero cases due to a stock-out do not mean zero transmission. Some studies have used model estimates to fill out missing data values [17]; in this paper, at the time of inclusion, only RHCs with 100% reporting completeness were chosen, thus attempting to minimize missing data, now 7.9%. Fifth, and finally, it is hard to determine the spread of interventions in the area investigated. Available information exists but only at a province level. Thus, it is hard to exclude that differences in transmission are not due to inequalities in spread of interventions. However, during 2011-2013 extensive mass test-and-treat interventions have taken place in the eastern parts of the province, where malaria thrives. Therefore, it is probable that the high transmission zone also have the highest intervention cover in Southern Province.

## Conclusion

It is demonstrated that remotely-sensed nocturnal dew point is significantly associated with malaria transmission in rural Zambia. Further investigation of environmental variables in real-time modelling could help create means to identify dry season foci of sustained transmission through active screening programmes.

## Competing interests

The authors declare that they have no competing interests.

## Authors’ contributions

DN performed all analyses, all modelling, and wrote the draft as well as visited health centres in Zambia. CS was the major contributor to the study design, as well as choice of modelling. CL was involved as support during the entire process. FM supervised the study, contributing with statistical knowledge and opinions. JM provided all malaria case data, population data and coordinates. AK was of great assistance in GIS-related questions and CJS initiated and coordinated the study. All authors read and approved the final manuscript.

## Supplementary Material

Additional file 1 **Log-file of high zone - model creation.** A log-file of the different steps undertaken to evaluate the best fit of the model for High transmission Zone. Click here for file

Additional file 2 Graphs of environment variables: graphs explaining temporal variations of environmental variables studied within zonal comparisons. Click here for file
