# Supplementary material for: Remotely-sensed, nocturnal, dew point correlates with malaria transmission in Southern Province, Zambia: a time-series study
Source: Malar J. 2014 Jun 13;13:231. doi: 10.1186/1475-2875-13-231 (PMC4078093; doi:10.1186/1475-2875-13-231)
Supplement: Additional file 1 — Log-file of high zone - model creation. A log-file of the different steps undertaken to evaluate the best fit of the model for High transmission Zone. [file 1475-2875-13-231-S1.pdf]

Remotely sensed nocturnal dew point correlates with malaria transmission in Southern Province, Zambia: a time-series study.
David Nygren

```
----------------------------------------------------------------------------------------------
-------------------------------------------------------------------------------------------
      name:  LOGfile of High Zone - model creation.
       log:  C:\Users\David\Google Drive\Zambia - ISEX T10\Data Monze + Choma\FINAL MODELS -
GRAPHS AND DOFILES\Log-file - exemplifying the process using High Zone.smcl
  log type:  smcl
 opened on:  27 Dec 2013, 12:38:45

. * Importing casedata and climate variables for the High Zone

.
. import excel "C:\Users\David\Google Drive\Zambia - ISEX T10\Data Monze +
Choma\GISexcel\INCwVariables.xlsx", sheet("HI") firstrow

.
. * Creating a time-series data-set.

.
. gen wtime1=substr(TIME, 1, 122)

.
. gen wtime=weekly(wtime1, "yw")

.
. format wtime %tw

.
. tsset wtime
        time variable:  wtime, 2011w19 to 2013w43
                delta:  1 week

.
. * log-transforming incidence data for normalization

.
. gen lnHi= ln(Hi)
(7 missing values generated)

.
. hist lnHi
(bin=11, start=1.7295599, width=.32318029)

.
.
.
. * Evaluating need of further differentiating making data stationary, a requirement for ARIMA-
modeling

.
. dfuller lnHi

Dickey-Fuller test for unit root                   Number of obs   =       121

                               ---------- Interpolated Dickey-Fuller ---------
                  Test         1% Critical       5% Critical      10% Critical
               Statistic           Value             Value             Value
------------------------------------------------------------------------------
 Z(t)             -2.193            -3.503            -2.889            -2.579
------------------------------------------------------------------------------
MacKinnon approximate p-value for Z(t) = 0.2087

.
.
.
. * Here we see that there is still a unit root, i.e a stochastic trend and we need to
differentiate the incidence for it to be stationary.
```

```
.
.
.
. * Next step is to look at the univariate correlations between incidence and environmental
variables as well as which lag times that hold significant patterns.

.
. * We use var to analyze this and create lagged variables when associations less than or
similar to p=0.1 are seen. We look at lags 1-11 weeks prior to incidence

.
. * but only use those with a biologically plausible coefficient and lag time.

.
.
.
. var lnHi DWP if tin(2012w19, 2013w36), lags(1/11)

Vector autoregression

Sample:  2012w19 - 2013w36                        No. of obs      =        70
Log likelihood = -145.0064                        AIC             =  5.457326
FPE            =  .8455142                        HQIC            =   6.04424
Det(Sigma_ml)  =  .2159488                        SBIC            =  6.934908

Equation           Parms      RMSE     R-sq      chi2     P>chi2
----------------------------------------------------------------
lnHi                 23      .30326   0.9069   681.7289   0.0000
DWP                  23     2.30242   0.8791   508.9686   0.0000
----------------------------------------------------------------

------------------------------------------------------------------------------
             |      Coef.   Std. Err.      z    P>|z|     [95% Conf. Interval]
-------------+----------------------------------------------------------------
lnHi         |
        lnHi |
         L1. |   .4478496   .1150198     3.89   0.000      .222415    .6732842
         L2. |   .3354528   .1251641     2.68   0.007     .0901357      .58077
         L3. |  -.0091347   .1285797    -0.07   0.943    -.2611464     .242877
         L4. |  -.1428361   .1300393    -1.10   0.272    -.3977084    .1120362
         L5. |  -.1707216   .1274569    -1.34   0.180    -.4205326    .0790893
         L6. |  -.0594373   .1280258    -0.46   0.642    -.3103632    .1914886
         L7. |   .1769897    .127949     1.38   0.167    -.0737858    .4277652
         L8. |  -.0265747   .1312195    -0.20   0.840    -.2837602    .2306109
         L9. |  -.1629274   .1299029    -1.25   0.210    -.4175323    .0916775
        L10. |  -.1048561   .1305525    -0.80   0.422    -.3607342    .1510221
        L11. |   .1898408   .1102198     1.72   0.085     -.026186    .4058677
             |
         DWP |
         L1. |  -.0066701   .0163248    -0.41   0.683    -.0386662     .025326
         L2. |   .0021479    .018726     0.11   0.909    -.0345544    .0388503
         L3. |   .0101005   .0186378     0.54   0.588    -.0264289    .0466298
         L4. |  -.0131142   .0170476    -0.77   0.442    -.0465268    .0202985
         L5. |    .009305   .0171747     0.54   0.588    -.0243568    .0429669
         L6. |   .0350728   .0174658     2.01   0.045     .0008405    .0693052
         L7. |   .0273658   .0175494     1.56   0.119    -.0070303     .061762
         L8. |  -.0172147   .0176606    -0.97   0.330    -.0518288    .0173993
         L9. |  -.0025995   .0180603    -0.14   0.886    -.0379971    .0327981
        L10. |   .0061923   .0192034     0.32   0.747    -.0314456    .0438303
        L11. |   .0224036   .0168475     1.33   0.184    -.0106168    .0554241
             |
       _cons |   1.497839   .4300111     3.48   0.000     .6550326    2.340645
-------------+----------------------------------------------------------------
DWP          |
        lnHi |
         L1. |   .1306424   .8732582     0.15   0.881    -1.580912    1.842197
         L2. |   .3712285   .9502765     0.39   0.696    -1.491279    2.233736
         L3. |  -2.136934   .9762089    -2.19   0.029    -4.050268   -.2235997
         L4. |   1.519299     .98729     1.54   0.124    -.4157539    3.454352
         L5. |   -.699657   .9676839    -0.72   0.470    -2.596283    1.196969
```

```
         L6. |  -.6808712   .9720029    -0.70   0.484    -2.585962     1.22422
         L7. |   .5663852   .9714204     0.58   0.560    -1.337564    2.470334
         L8. |   .1146423   .9962507     0.12   0.908    -1.837973    2.067258
         L9. |  -.5955843   .9862542    -0.60   0.546    -2.528607    1.337438
        L10. |  -1.401578   .9911865    -1.41   0.157    -3.344268    .5411119
        L11. |   .6365379   .8368158     0.76   0.447    -1.003591    2.276667
             |
         DWP |
         L1. |   .6057632   .1239421     4.89   0.000      .362841    .8486853
         L2. |   .1949611   .1421726     1.37   0.170    -.0836921    .4736144
         L3. |  -.1178905   .1415024    -0.83   0.405    -.3952301    .1594491
         L4. |   .0708605   .1294294     0.55   0.584    -.1828164    .3245374
         L5. |   .1749345   .1303949     1.34   0.180    -.0806347    .4305038
         L6. |   -.061729   .1326048    -0.47   0.642    -.3216295    .1981715
         L7. |  -.0328258   .1332392    -0.25   0.805    -.2939697    .2283182
         L8. |   .2647082   .1340833     1.97   0.048     .0019098    .5275065
         L9. |  -.4156697   .1371185    -3.03   0.002     -.684417   -.1469225
        L10. |   .3383101   .1457967     2.32   0.020     .0525539    .6240663
        L11. |  -.0165741     .12791    -0.13   0.897    -.2672731     .234125
             |
       _cons |   7.745701   3.264749     2.37   0.018      1.34691    14.14449
------------------------------------------------------------------------------

.
. gen L6DWP=L6.DWP
(6 missing values generated)

.
. gen L7DWP=L7.DWP
(7 missing values generated)

.
.
.
. var lnHi EVI if tin(2012w19, 2013w36), lags(1/11)

Vector autoregression

Sample:  2012w19 - 2013w35                          No. of obs      =        69
Log likelihood =  174.8125                          AIC             = -3.733695
FPE            =  .0000864                          HQIC            = -3.142799
Det(Sigma_ml)  =  .0000216                          SBIC            = -2.244291

Equation           Parms      RMSE     R-sq      chi2     P>chi2
----------------------------------------------------------------
lnHi                 23     .326867   0.8891   553.4116   0.0000
EVI                  23     .022266   0.9670   2023.436   0.0000
----------------------------------------------------------------

------------------------------------------------------------------------------
             |      Coef.   Std. Err.      z    P>|z|     [95% Conf. Interval]
-------------+----------------------------------------------------------------
lnHi         |
        lnHi |
         L1. |   .7282734   .1234437     5.90   0.000     .4863283    .9702186
         L2. |   .2244119   .1467237     1.53   0.126    -.0631613    .5119851
         L3. |  -.1092487   .1379237    -0.79   0.428    -.3795742    .1610768
         L4. |    .017089   .1394745     0.12   0.902     -.256276     .290454
         L5. |   -.140225   .1364614    -1.03   0.304    -.4076845    .1272345
         L6. |   .0605324   .1387878     0.44   0.663    -.2114867    .3325514
         L7. |   .2432314   .1435545     1.69   0.090    -.0381303    .5245931
         L8. |  -.2545845   .1457994    -1.75   0.081    -.5403461    .0311771
         L9. |  -.0457895   .1537057    -0.30   0.766    -.3470472    .2554681
        L10. |   .0050498   .1397251     0.04   0.971    -.2688065     .278906
        L11. |    .124205   .1187349     1.05   0.296    -.1085111    .3569211
             |
         EVI |
         L1. |  -2.100534   1.810096    -1.16   0.246    -5.648258     1.44719
         L2. |   4.209475   2.139173     1.97   0.049     .0167735    8.402176
         L3. |  -1.840505   2.148687    -0.86   0.392    -6.051854    2.370845
         L4. |   1.760354   2.022775     0.87   0.384    -2.204213    5.724921
```

```
        L5. |   3.480571   2.382102     1.46   0.144    -1.188262    8.149405
        L6. |  -5.343221   2.340576    -2.28   0.022    -9.930665   -.7557772
        L7. |   2.603986   2.380513     1.09   0.274    -2.061733    7.269706
        L8. |   .9706414   1.942472     0.50   0.617    -2.836533    4.777816
        L9. |  -6.309196   2.031447    -3.11   0.002    -10.29076   -2.327633
       L10. |   4.716474   2.184002     2.16   0.031     .4359098    8.997039
       L11. |  -1.217352   1.840499    -0.66   0.508    -4.824665     2.38996
            |
      _cons |   .2183677   .2428276     0.90   0.369    -.2575656     .694301
-------------+----------------------------------------------------------------
EVI          |
        lnHi |
         L1. |   .0108323   .0084089     1.29   0.198    -.0056488    .0273134
         L2. |  -.0108705   .0099947    -1.09   0.277    -.0304598    .0087187
         L3. |   .0003939   .0093953     0.04   0.967    -.0180205    .0188083
         L4. |   .0032353   .0095009     0.34   0.733    -.0153861    .0218567
         L5. |   .0044601   .0092956     0.48   0.631    -.0137591    .0226792
         L6. |  -.0029292   .0094541    -0.31   0.757     -.021459    .0156005
         L7. |   .0037072   .0097788     0.38   0.705     -.015459    .0228733
         L8. |  -.0040519   .0099317    -0.41   0.683    -.0235177     .015414
         L9. |   -.000224   .0104703    -0.02   0.983    -.0207454    .0202974
        L10. |   .0125508    .009518     1.32   0.187    -.0061041    .0312057
        L11. |  -.0085834   .0080881    -1.06   0.289    -.0244358    .0072691
             |
         EVI |
         L1. |    .596644   .1233023     4.84   0.000     .3549758    .8383121
         L2. |   .1977649   .1457187     1.36   0.175    -.0878386    .4833684
         L3. |   .0999815   .1463669     0.68   0.495    -.1868923    .3868553
         L4. |   .7328531   .1377898     5.32   0.000     .4627899    1.002916
         L5. |  -.2333653   .1622669    -1.44   0.150    -.5514025    .0846719
         L6. |  -.2627655   .1594382    -1.65   0.099    -.5752585    .0497276
         L7. |  -.1254759   .1621587    -0.77   0.439     -.443301    .1923492
         L8. |   -.329755   .1323196    -2.49   0.013    -.5890967   -.0704134
         L9. |    .059779   .1383806     0.43   0.666    -.2114419    .3309999
        L10. |   .0990438   .1487725     0.67   0.506    -.1925448    .3906325
        L11. |   .0192087   .1253734     0.15   0.878    -.2265186    .2649359
             |
       _cons |   .0131866   .0165412     0.80   0.425    -.0192336    .0456068
------------------------------------------------------------------------------

.
. gen L10EVI=L10.EVI
(10 missing values generated)

.
.
.
. var lnHi LST if tin(2012w19, 2013w36), lags(1/11)

Vector autoregression

Sample:  2012w19 - 2013w36                         No. of obs      =        70
Log likelihood = -93.43695                         AIC             =  3.983913
FPE            =  .1937429                         HQIC            =  4.570827
Det(Sigma_ml)  =   .049483                         SBIC            =  5.461495

Equation           Parms      RMSE     R-sq      chi2     P>chi2
----------------------------------------------------------------
lnHi                 23     .303745   0.9066   679.3304   0.0000
LST                  23      1.1214   0.9193    797.699   0.0000
----------------------------------------------------------------

------------------------------------------------------------------------------
             |      Coef.   Std. Err.      z    P>|z|     [95% Conf. Interval]
-------------+----------------------------------------------------------------
lnHi         |
        lnHi |
         L1. |   .5954682   .1159689     5.13   0.000     .3681734     .822763
         L2. |   .4355438   .1387415     3.14   0.002     .1636155     .707472
         L3. |  -.0539089   .1349101    -0.40   0.689    -.3183279    .2105101
         L4. |  -.1307362   .1341624    -0.97   0.330    -.3936897    .1322173
```

```
         L5. |  -.0959831   .1261795    -0.76   0.447    -.3432903    .1513242
         L6. |   .0177119   .1255749     0.14   0.888    -.2284102    .2638341
         L7. |   .1349108   .1268597     1.06   0.288    -.1137296    .3835513
         L8. |  -.0562185   .1340666    -0.42   0.675    -.3189842    .2065471
         L9. |  -.2003127   .1329589    -1.51   0.132    -.4609073     .060282
        L10. |    .031677   .1334977     0.24   0.812    -.2299737    .2933278
        L11. |    .209614   .1183524     1.77   0.077    -.0223525    .4415805
             |
         LST |
         L1. |   .0401948   .0320676     1.25   0.210    -.0226565    .1030462
         L2. |  -.0714051   .0367968    -1.94   0.052    -.1435255    .0007153
         L3. |   .0610564   .0380978     1.60   0.109     -.013614    .1357268
         L4. |  -.0220908   .0384823    -0.57   0.566    -.0975146    .0533331
         L5. |   .0206853     .03635     0.57   0.569    -.0505593      .09193
         L6. |  -.0035974   .0377154    -0.10   0.924    -.0775183    .0703234
         L7. |   .1039718   .0385709     2.70   0.007     .0283742    .1795695
         L8. |  -.0182399   .0407487    -0.45   0.654     -.098106    .0616262
         L9. |  -.1044516   .0413544    -2.53   0.012    -.1855047   -.0233984
        L10. |   .0082784   .0423192     0.20   0.845    -.0746658    .0912225
        L11. |   .0523438   .0339324     1.54   0.123    -.0141624      .11885
             |
       _cons |  -.8539527    .460909    -1.85   0.064    -1.757318    .0494123
-------------+----------------------------------------------------------------
LST          |
        lnHi |
         L1. |  -.2465946   .4281477    -0.58   0.565    -1.085749    .5925593
         L2. |   .2460483   .5122222     0.48   0.631    -.7578887    1.249985
         L3. |   .1813626   .4980773     0.36   0.716    -.7948509    1.157576
         L4. |  -.2774781   .4953167    -0.56   0.575    -1.248281    .6933249
         L5. |  -.0956666   .4658445    -0.21   0.837    -1.008705    .8173718
         L6. |   .5219866   .4636122     1.13   0.260    -.3866765     1.43065
         L7. |   -1.03674   .4683558    -2.21   0.027      -1.9547   -.1187795
         L8. |   .5535659   .4949629     1.12   0.263    -.4165435    1.523675
         L9. |   .7364477   .4908734     1.50   0.134    -.2256465    1.698542
        L10. |  -.6686946   .4928628    -1.36   0.175    -1.634688    .2972988
        L11. |  -.7933353   .4369476    -1.82   0.069    -1.649737    .0630662
             |
         LST |
         L1. |   .6017886    .118391     5.08   0.000     .3697465    .8338306
         L2. |   .2441571   .1358508     1.80   0.072    -.0221056    .5104199
         L3. |   .0070809   .1406541     0.05   0.960    -.2685962    .2827579
         L4. |  -.0809593   .1420734    -0.57   0.569     -.359418    .1974993
         L5. |   .0131396   .1342012     0.10   0.922      -.24989    .2761692
         L6. |   -.001116   .1392423    -0.01   0.994     -.274026    .2717939
         L7. |   .1110869   .1424008     0.78   0.435    -.1680135    .3901872
         L8. |   .0056032   .1504411     0.04   0.970    -.2892559    .3004623
         L9. |  -.2095125   .1526771    -1.37   0.170    -.5087542    .0897291
        L10. |  -.0631968   .1562391    -0.40   0.686    -.3694198    .2430261
        L11. |   .1065035   .1252755     0.85   0.395     -.139032     .352039
             |
       _cons |   8.107776   1.701638     4.76   0.000     4.772626    11.44293
------------------------------------------------------------------------------

.
. gen L7LST=L7.LST
(7 missing values generated)

.
.
.
. var lnHi NDVI if tin(2012w19, 2013w36), lags(1/11)

Vector autoregression

Sample:  2012w19 - 2013w36                        No. of obs      =        70
Log likelihood =   164.896                        AIC             = -3.397029
FPE            =  .0001207                        HQIC            = -2.810115
Det(Sigma_ml)  =  .0000308                        SBIC            = -1.919447

Equation           Parms      RMSE     R-sq      chi2     P>chi2
----------------------------------------------------------------
```

```
lnHi                23      .29801   0.9101   708.4448   0.0000
NDVI                23     .028215   0.9642    1883.57   0.0000
-----------------------------------------------------------------

------------------------------------------------------------------------------
             |      Coef.   Std. Err.      z    P>|z|     [95% Conf. Interval]
-------------+----------------------------------------------------------------
lnHi         |
        lnHi |
         L1. |   .6478695   .1173855     5.52   0.000      .417798    .8779409
         L2. |   .2670647   .1352566     1.97   0.048     .0019667    .5321627
         L3. |  -.0801773   .1250103    -0.64   0.521     -.325193    .1648385
         L4. |    .032128   .1238746     0.26   0.795    -.2106618    .2749177
         L5. |  -.1499056   .1216782    -1.23   0.218    -.3883905    .0885792
         L6. |   .0388958   .1234998     0.31   0.753    -.2031594     .280951
         L7. |   .2728254   .1255873     2.17   0.030     .0266789    .5189719
         L8. |  -.2182587   .1333239    -1.64   0.102    -.4795688    .0430514
         L9. |  -.0844528   .1339531    -0.63   0.528    -.3469962    .1780905
        L10. |   .0220908   .1271504     0.17   0.862    -.2271194    .2713009
        L11. |    .210367   .1104641     1.90   0.057    -.0061386    .4268726
             |
        NDVI |
         L1. |  -2.055607     1.2305    -1.67   0.095    -4.467342    .3561281
         L2. |    1.64827   1.446066     1.14   0.254    -1.185967    4.482508
         L3. |  -1.271288   1.413702    -0.90   0.369    -4.042093    1.499517
         L4. |   1.911812   1.382224     1.38   0.167    -.7972977    4.620921
         L5. |   3.800446   1.529686     2.48   0.013     .8023167    6.798576
         L6. |  -2.352412   1.568563    -1.50   0.134     -5.42674    .7219156
         L7. |   2.391342   1.583975     1.51   0.131    -.7131909    5.495875
         L8. |   .7320028    1.38758     0.53   0.598    -1.987605    3.451611
         L9. |  -6.259987   1.418803    -4.41   0.000    -9.040789   -3.479185
        L10. |   2.472254   1.629339     1.52   0.129    -.7211908    5.665699
        L11. |  -1.602208   1.384862    -1.16   0.247    -4.316487    1.112071
             |
       _cons |    .402037   .2111031     1.90   0.057    -.0117175    .8157915
-------------+----------------------------------------------------------------
NDVI         |
        lnHi |
         L1. |   .0109094   .0111137     0.98   0.326     -.010873    .0326918
         L2. |  -.0104558   .0128057    -0.82   0.414    -.0355544    .0146429
         L3. |   .0027026   .0118356     0.23   0.819    -.0204947    .0258999
         L4. |   .0041993   .0117281     0.36   0.720    -.0187872    .0271859
         L5. |   .0082316   .0115201     0.71   0.475    -.0143474    .0308106
         L6. |  -.0074295   .0116926    -0.64   0.525    -.0303466    .0154875
         L7. |  -.0005792   .0118902    -0.05   0.961    -.0238836    .0227251
         L8. |   .0049267   .0126227     0.39   0.696    -.0198133    .0296667
         L9. |  -.0000739   .0126823    -0.01   0.995    -.0249306    .0247829
        L10. |   .0136786   .0120382     1.14   0.256    -.0099159     .037273
        L11. |  -.0034295   .0104584    -0.33   0.743    -.0239275    .0170686
             |
        NDVI |
         L1. |   .5985058   .1164998     5.14   0.000     .3701704    .8268412
         L2. |   .1360931   .1369089     0.99   0.320    -.1322434    .4044297
         L3. |   .0463343   .1338448     0.35   0.729    -.2159967    .3086652
         L4. |   .5105407   .1308646     3.90   0.000     .2540509    .7670305
         L5. |  -.0702115   .1448258    -0.48   0.628    -.3540649    .2136419
         L6. |  -.1388223   .1485066    -0.93   0.350    -.4298898    .1522453
         L7. |  -.0512604   .1499657    -0.34   0.732    -.3451877    .2426669
         L8. |  -.1965712   .1313717    -1.50   0.135     -.454055    .0609126
         L9. |   .1306462   .1343277     0.97   0.331    -.1326313    .3939237
        L10. |  -.1127831   .1542606    -0.73   0.465    -.4151282    .1895621
        L11. |  -.1088519   .1311143    -0.83   0.406    -.3658312    .1481274
             |
       _cons |   .0424343   .0199866     2.12   0.034     .0032613    .0816072
------------------------------------------------------------------------------

.
. gen L5NDVI=L5.NDVI
(5 missing values generated)

.
```

```
. gen L7NDVI=L7.NDVI
(7 missing values generated)

.
.
.
. var lnHi RAINFALL if tin(2012w19, 2013w36), lags(1/11)

Vector autoregression

Sample:  2012w19 - 2013w35                          No. of obs      =        69
Log likelihood = -332.5476                          AIC             =  10.97239
FPE            =  210.5241                          HQIC            =  11.56329
Det(Sigma_ml)  =  52.63102                          SBIC            =   12.4618

Equation           Parms      RMSE     R-sq      chi2     P>chi2
----------------------------------------------------------------
lnHi                 23      .33972   0.8803   507.2035   0.0000
RAINFALL             23     32.2359   0.8603   424.7591   0.0000
----------------------------------------------------------------

------------------------------------------------------------------------------
             |      Coef.   Std. Err.      z    P>|z|     [95% Conf. Interval]
-------------+----------------------------------------------------------------
lnHi         |
        lnHi |
         L1. |   .7666585   .1203811     6.37   0.000     .5307159    1.002601
         L2. |    .347196   .1482184     2.34   0.019     .0566933    .6376987
         L3. |  -.1072091   .1433246    -0.75   0.454    -.3881202    .1737019
         L4. |  -.0537564   .1437034    -0.37   0.708    -.3354098    .2278971
         L5. |  -.1139687   .1432925    -0.80   0.426    -.3948169    .1668794
         L6. |   .1167411   .1449544     0.81   0.421    -.1673643    .4008466
         L7. |   .1667119   .1489607     1.12   0.263    -.1252457    .4586694
         L8. |  -.3121845   .1491634    -2.09   0.036    -.6045394   -.0198295
         L9. |  -.0325593   .1600052    -0.20   0.839    -.3461637    .2810451
        L10. |   .0459889   .1482715     0.31   0.756    -.2446179    .3365958
        L11. |   .0301317   .1223463     0.25   0.805    -.2096626    .2699259
             |
    RAINFALL |
         L1. |  -.0008884   .0012091    -0.73   0.463    -.0032582    .0014815
         L2. |   .0003679   .0015574     0.24   0.813    -.0026846    .0034204
         L3. |   .0018237   .0015504     1.18   0.239    -.0012151    .0048625
         L4. |  -.0003084   .0015416    -0.20   0.841    -.0033298     .002713
         L5. |  -.0005486   .0015224    -0.36   0.719    -.0035325    .0024353
         L6. |  -.0002668   .0015381    -0.17   0.862    -.0032813    .0027478
         L7. |   .0017351   .0015345     1.13   0.258    -.0012725    .0047427
         L8. |   .0015445   .0015454     1.00   0.318    -.0014845    .0045734
         L9. |  -.0054244   .0015501    -3.50   0.000    -.0084625   -.0023862
        L10. |   .0027332   .0016809     1.63   0.104    -.0005613    .0060278
        L11. |   -.000067   .0012595    -0.05   0.958    -.0025355    .0024015
             |
       _cons |   .4656873   .3299495     1.41   0.158    -.1810018    1.112376
-------------+----------------------------------------------------------------
RAINFALL     |
        lnHi |
         L1. |   16.16858   11.42292     1.42   0.157    -6.219933     38.5571
         L2. |  -9.373096   14.06439    -0.67   0.505    -36.93879     18.1926
         L3. |   7.501239   13.60002     0.55   0.581    -19.15431    34.15678
         L4. |    16.7284   13.63596     1.23   0.220    -9.997588    43.45439
         L5. |  -4.288894   13.59697    -0.32   0.752    -30.93847    22.36069
         L6. |  -1.730918   13.75467    -0.13   0.900    -28.68958    25.22774
         L7. |   -11.9689   14.13482    -0.85   0.397    -39.67264    15.73484
         L8. |   18.89885   14.15406     1.34   0.182    -8.842605     46.6403
         L9. |  -1.905748   15.18283    -0.13   0.900    -31.66355    27.85205
        L10. |  -8.187956   14.06943    -0.58   0.561    -35.76353    19.38762
        L11. |   2.628069   11.60939     0.23   0.821    -20.12592    25.38206
             |
    RAINFALL |
         L1. |    .806386   .1147348     7.03   0.000     .5815099    1.031262
         L2. |  -.0163885   .1477828    -0.11   0.912    -.3060373    .2732604
         L3. |  -.0592322   .1471198    -0.40   0.687    -.3475817    .2291174
```

```
         L4. |  -.0808102   .1462784    -0.55   0.581    -.3675106    .2058901
         L5. |   .1531967   .1444602     1.06   0.289    -.1299401    .4363335
         L6. |  -.0475661   .1459462    -0.33   0.744    -.3336155    .2384833
         L7. |  -.0480017   .1456082    -0.33   0.742    -.3333885    .2373852
         L8. |   .0755903   .1466449     0.52   0.606    -.2118284    .3630091
         L9. |  -.2400493   .1470882    -1.63   0.103     -.528337    .0482384
        L10. |   .1853549   .1595005     1.16   0.245    -.1272603    .4979701
        L11. |  -.1739681   .1195095    -1.46   0.145    -.4082023    .0602662
             |
       _cons |   -64.1134   31.30879    -2.05   0.041    -125.4775   -2.749304
------------------------------------------------------------------------------

.
. gen L10RAINFALL=L10.RAINFALL
(10 missing values generated)

.
.
.
. *** These environmental variables are then tested in multivariate ARIMA-models -> ARIMAX.
These are

.
. * compared regarding to the Akaike's information criteria, with the lowest indicating the best
fit. The two best multivariate

.
. * models are chosen for further evaluation. Estimates stats show an overview. All use 1,1,1 as
arima-parameters now.

.
.
.
. ** Testing ARIMA-models with different nondifferentiated variables

.
.
.
. arima lnHi L6DWP L7DWP L10EVI L7LST L5NDVI L7NDVI if tin(2012w19, 2013w36), arima(1,1,1)

(setting optimization to BHHH)
Iteration 0:   log likelihood = -9.3104415
Iteration 1:   log likelihood = -8.4921827
Iteration 2:   log likelihood = -7.6024975
Iteration 3:   log likelihood = -7.2907722
Iteration 4:   log likelihood = -7.2122104
(switching optimization to BFGS)
Iteration 5:   log likelihood = -7.1883553
Iteration 6:   log likelihood = -7.1710588
Iteration 7:   log likelihood = -7.1696317
Iteration 8:   log likelihood =   -7.16855
Iteration 9:   log likelihood =  -7.168534
Iteration 10:  log likelihood = -7.1685148
Iteration 11:  log likelihood = -7.1685143

ARIMA regression

Sample:  2012w19 - 2013w36                      Number of obs      =        70
                                                Wald chi2(8)       =     43.02
Log likelihood = -7.168514                      Prob > chi2        =    0.0000

------------------------------------------------------------------------------
             |                 OPG
      D.lnHi |      Coef.   Std. Err.      z    P>|z|     [95% Conf. Interval]
-------------+----------------------------------------------------------------
lnHi         |
       L6DWP |
         D1. |   .0137968   .0183838     0.75   0.453    -.0222349    .0498284
             |
       L7DWP |
         D1. |    .027144   .0159155     1.71   0.088    -.0040498    .0583378
```

```
             |
      L10EVI |
         D1. |   -.769521   1.691665    -0.45   0.649    -4.085124    2.546082
             |
       L7LST |
         D1. |   .0820248    .032653     2.51   0.012     .0180261    .1460235
             |
      L5NDVI |
         D1. |    1.25172   1.013452     1.24   0.217    -.7346093    3.238049
             |
      L7NDVI |
         D1. |   2.373584   1.277386     1.86   0.063    -.1300453    4.877214
             |
       _cons |  -.0077443   .0257445    -0.30   0.764    -.0582026     .042714
-------------+----------------------------------------------------------------
ARMA         |
          ar |
         L1. |  -.5491054   .2400446    -2.29   0.022    -1.019584   -.0786268
             |
          ma |
         L1. |   .0880126   .3363914     0.26   0.794    -.5713024    .7473275
-------------+----------------------------------------------------------------
      /sigma |   .2675588   .0289992     9.23   0.000     .2107214    .3243962
------------------------------------------------------------------------------
Note: The test of the variance against zero is one sided, and the two-sided confidence interval
is truncated at zero.

.
. estat ic

-----------------------------------------------------------------------------
       Model |    Obs    ll(null)   ll(model)     df          AIC         BIC
-------------+---------------------------------------------------------------
           . |     70           .   -7.168514     10     34.33703    56.82198
-----------------------------------------------------------------------------
               Note:  N=Obs used in calculating BIC; see [R] BIC note

.
. estimates store M1

.
.
.
. arima lnHi L6DWP L7DWP L7LST L5NDVI L7NDVI if tin(2012w19, 2013w36), arima(1,1,1)

(setting optimization to BHHH)
Iteration 0:   log likelihood =  -8.723498
Iteration 1:   log likelihood = -8.0209724
Iteration 2:   log likelihood = -7.7574323
Iteration 3:   log likelihood = -7.4103456
Iteration 4:   log likelihood = -7.3931155
(switching optimization to BFGS)
Iteration 5:   log likelihood = -7.3730476
Iteration 6:   log likelihood = -7.3555546
Iteration 7:   log likelihood = -7.3553441
Iteration 8:   log likelihood = -7.3552553
Iteration 9:   log likelihood = -7.3552321
Iteration 10:  log likelihood = -7.3552286
Iteration 11:  log likelihood = -7.3552284

ARIMA regression

Sample:  2012w19 - 2013w36                      Number of obs      =        70
                                                Wald chi2(7)       =     39.76
Log likelihood = -7.355228                      Prob > chi2        =    0.0000

------------------------------------------------------------------------------
             |                 OPG
      D.lnHi |      Coef.   Std. Err.      z    P>|z|     [95% Conf. Interval]
-------------+----------------------------------------------------------------
lnHi         |
```

```
       L6DWP |
         D1. |   .0124134   .0183189     0.68   0.498    -.0234909    .0483178
             |
       L7DWP |
         D1. |   .0259898   .0159425     1.63   0.103    -.0052569    .0572366
             |
       L7LST |
         D1. |   .0845866   .0324404     2.61   0.009     .0210046    .1481687
             |
      L5NDVI |
         D1. |   .9839166   .9487261     1.04   0.300    -.8755524    2.843386
             |
      L7NDVI |
         D1. |   2.192994   1.329916     1.65   0.099    -.4135928     4.79958
             |
       _cons |  -.0076684   .0264469    -0.29   0.772    -.0595034    .0441666
-------------+----------------------------------------------------------------
ARMA         |
          ar |
         L1. |  -.5214658    .262344    -1.99   0.047    -1.035651    -.007281
             |
          ma |
         L1. |   .0860998   .3497618     0.25   0.806    -.5994208    .7716203
-------------+----------------------------------------------------------------
      /sigma |   .2683345   .0289748     9.26   0.000      .211545    .3251239
------------------------------------------------------------------------------
Note: The test of the variance against zero is one sided, and the two-sided confidence interval
is truncated at zero.

.
. estat ic

-----------------------------------------------------------------------------
       Model |    Obs    ll(null)   ll(model)     df          AIC         BIC
-------------+---------------------------------------------------------------
           . |     70           .   -7.355228      9     32.71046    52.94691
-----------------------------------------------------------------------------
               Note:  N=Obs used in calculating BIC; see [R] BIC note

.
. estimates store M2

.
.
.
. arima lnHi L7DWP L7LST L5NDVI L7NDVI if tin(2012w19, 2013w36), arima(1,1,1)

(setting optimization to BHHH)
Iteration 0:   log likelihood = -9.1384455
Iteration 1:   log likelihood = -8.0710161
Iteration 2:   log likelihood =  -7.763473
Iteration 3:   log likelihood = -7.7360739
Iteration 4:   log likelihood =  -7.728834
(switching optimization to BFGS)
Iteration 5:   log likelihood =  -7.722913
Iteration 6:   log likelihood = -7.7170635
Iteration 7:   log likelihood = -7.7169507
Iteration 8:   log likelihood = -7.7169214
Iteration 9:   log likelihood = -7.7169148
Iteration 10:  log likelihood = -7.7169145

ARIMA regression

Sample:  2012w19 - 2013w36                      Number of obs      =        70
                                                Wald chi2(6)       =     39.04
Log likelihood = -7.716914                      Prob > chi2        =    0.0000

------------------------------------------------------------------------------
             |                 OPG
      D.lnHi |      Coef.   Std. Err.      z    P>|z|     [95% Conf. Interval]
-------------+----------------------------------------------------------------
```

```
lnHi         |
       L7DWP |
         D1. |   .0270427   .0167322     1.62   0.106    -.0057517    .0598371
             |
       L7LST |
         D1. |   .0948768   .0278555     3.41   0.001     .0402811    .1494725
             |
      L5NDVI |
         D1. |   1.220218   .8941401     1.36   0.172    -.5322646      2.9727
             |
      L7NDVI |
         D1. |    2.11968   1.307911     1.62   0.105    -.4437791    4.683139
             |
       _cons |  -.0084875   .0261738    -0.32   0.746    -.0597873    .0428123
-------------+----------------------------------------------------------------
ARMA         |
          ar |
         L1. |  -.5004121   .2638259    -1.90   0.058    -1.017501    .0166772
             |
          ma |
         L1. |   .0616274   .3473492     0.18   0.859    -.6191646    .7424194
-------------+----------------------------------------------------------------
      /sigma |   .2697287   .0279892     9.64   0.000      .214871    .3245864
------------------------------------------------------------------------------
Note: The test of the variance against zero is one sided, and the two-sided confidence interval
is truncated at zero.

.
. estat ic

-----------------------------------------------------------------------------
       Model |    Obs    ll(null)   ll(model)     df          AIC         BIC
-------------+---------------------------------------------------------------
           . |     70           .   -7.716914      8     31.43383    49.42179
-----------------------------------------------------------------------------
               Note:  N=Obs used in calculating BIC; see [R] BIC note

.
. estimates store M3

.
.
.
. arima lnHi L7DWP L7LST L7NDVI if tin(2012w19, 2013w36), arima(1,1,1)

(setting optimization to BHHH)
Iteration 0:   log likelihood = -9.7522477
Iteration 1:   log likelihood = -9.0313967
Iteration 2:   log likelihood = -8.7719251
Iteration 3:   log likelihood = -8.7102241
Iteration 4:   log likelihood = -8.7059065
(switching optimization to BFGS)
Iteration 5:   log likelihood = -8.6825257
Iteration 6:   log likelihood = -8.6558003
Iteration 7:   log likelihood = -8.6556826
Iteration 8:   log likelihood = -8.6555932
Iteration 9:   log likelihood = -8.6555869
Iteration 10:  log likelihood = -8.6555858
Iteration 11:  log likelihood = -8.6555857

ARIMA regression

Sample:  2012w19 - 2013w36                      Number of obs      =        70
                                                Wald chi2(5)       =     41.82
Log likelihood = -8.655586                      Prob > chi2        =    0.0000

------------------------------------------------------------------------------
             |                 OPG
      D.lnHi |      Coef.   Std. Err.      z    P>|z|     [95% Conf. Interval]
-------------+----------------------------------------------------------------
lnHi         |
```

```
       L7DWP |
         D1. |   .0323079     .01793     1.80   0.072    -.0028342      .06745
             |
       L7LST |
         D1. |   .0929454   .0253781     3.66   0.000     .0432053    .1426855
             |
      L7NDVI |
         D1. |   1.960931   1.324157     1.48   0.139    -.6343693    4.556231
             |
       _cons |  -.0137697   .0266529    -0.52   0.605    -.0660084     .038469
-------------+----------------------------------------------------------------
ARMA         |
          ar |
         L1. |  -.4649715   .2826193    -1.65   0.100    -1.018895    .0889521
             |
          ma |
         L1. |   .0504677    .361023     0.14   0.889    -.6571243    .7580597
-------------+----------------------------------------------------------------
      /sigma |   .2734307   .0260862    10.48   0.000     .2223027    .3245587
------------------------------------------------------------------------------
Note: The test of the variance against zero is one sided, and the two-sided confidence interval
is truncated at zero.

.
. estat ic

-----------------------------------------------------------------------------
       Model |    Obs    ll(null)   ll(model)     df          AIC         BIC
-------------+---------------------------------------------------------------
           . |     70           .   -8.655586      7     31.31117    47.05064
-----------------------------------------------------------------------------
               Note:  N=Obs used in calculating BIC; see [R] BIC note

.
. estimates store M4

.
.
.
. arima lnHi L7DWP L7LST if tin(2012w19, 2013w36), arima(1,1,1)

(setting optimization to BHHH)
Iteration 0:   log likelihood = -11.513957
Iteration 1:   log likelihood = -11.005714
Iteration 2:   log likelihood = -10.901356
Iteration 3:   log likelihood = -10.893997
Iteration 4:   log likelihood = -10.890163
(switching optimization to BFGS)
Iteration 5:   log likelihood = -10.889136
Iteration 6:   log likelihood = -10.888497
Iteration 7:   log likelihood = -10.888494
Iteration 8:   log likelihood = -10.888494

ARIMA regression

Sample:  2012w19 - 2013w36                      Number of obs      =        70
                                                Wald chi2(4)       =     32.56
Log likelihood = -10.88849                      Prob > chi2        =    0.0000

------------------------------------------------------------------------------
             |                 OPG
      D.lnHi |      Coef.   Std. Err.      z    P>|z|     [95% Conf. Interval]
-------------+----------------------------------------------------------------
lnHi         |
       L7DWP |
         D1. |   .0352464   .0167862     2.10   0.036      .002346    .0681468
             |
       L7LST |
         D1. |    .103655   .0251477     4.12   0.000     .0543665    .1529435
             |
       _cons |  -.0208092   .0257976    -0.81   0.420    -.0713715    .0297531
```

```
-------------+----------------------------------------------------------------
ARMA         |
          ar |
         L1. |  -.4149715    .333382    -1.24   0.213    -1.068388    .2384452
             |
          ma |
         L1. |   .0601303   .3979042     0.15   0.880    -.7197476    .8400082
-------------+----------------------------------------------------------------
      /sigma |   .2824101   .0290265     9.73   0.000     .2255192    .3393011
------------------------------------------------------------------------------
Note: The test of the variance against zero is one sided, and the two-sided confidence interval
is truncated at zero.

.
. estat ic

-----------------------------------------------------------------------------
       Model |    Obs    ll(null)   ll(model)     df          AIC         BIC
-------------+---------------------------------------------------------------
           . |     70           .   -10.88849      6     33.77699    47.26796
-----------------------------------------------------------------------------
               Note:  N=Obs used in calculating BIC; see [R] BIC note

.
. estimates store M5

.
.
.
. arima lnHi L7LST if tin(2012w19, 2013w36), arima(1,1,1)

(setting optimization to BHHH)
Iteration 0:   log likelihood = -15.302867
Iteration 1:   log likelihood = -14.664458
Iteration 2:   log likelihood = -14.046832
Iteration 3:   log likelihood = -14.018271
Iteration 4:   log likelihood = -13.995699
(switching optimization to BFGS)
Iteration 5:   log likelihood = -13.994925
Iteration 6:   log likelihood = -13.994029
Iteration 7:   log likelihood =  -13.99401
Iteration 8:   log likelihood =  -13.99401

ARIMA regression

Sample:  2012w19 - 2013w36                      Number of obs      =        70
                                                Wald chi2(3)       =     33.39
Log likelihood = -13.99401                      Prob > chi2        =    0.0000

------------------------------------------------------------------------------
             |                 OPG
      D.lnHi |      Coef.   Std. Err.      z    P>|z|     [95% Conf. Interval]
-------------+----------------------------------------------------------------
lnHi         |
       L7LST |
         D1. |   .1235786   .0249941     4.94   0.000     .0745911    .1725661
             |
       _cons |  -.0260946   .0278617    -0.94   0.349    -.0807026    .0285133
-------------+----------------------------------------------------------------
ARMA         |
          ar |
         L1. |  -.4297409   .3398264    -1.26   0.206    -1.095788    .2363065
             |
          ma |
         L1. |   .1053449   .3947713     0.27   0.790    -.6683927    .8790825
-------------+----------------------------------------------------------------
      /sigma |   .2952518   .0326295     9.05   0.000     .2312992    .3592044
------------------------------------------------------------------------------
Note: The test of the variance against zero is one sided, and the two-sided confidence interval
is truncated at zero.
```

```
.
. estat ic

-----------------------------------------------------------------------------
       Model |    Obs    ll(null)   ll(model)     df          AIC         BIC
-------------+---------------------------------------------------------------
           . |     70           .   -13.99401      5     37.98802     49.2305
-----------------------------------------------------------------------------
               Note:  N=Obs used in calculating BIC; see [R] BIC note

.
. estimates store M6

.
.
.
. arima lnHi if tin(2012w19, 2013w36), arima(1,1,1)

(setting optimization to BHHH)
Iteration 0:   log likelihood =  -22.53879
Iteration 1:   log likelihood = -21.170514
Iteration 2:   log likelihood = -21.110811
Iteration 3:   log likelihood = -21.107847
Iteration 4:   log likelihood =   -21.1073
(switching optimization to BFGS)
Iteration 5:   log likelihood = -21.107196
Iteration 6:   log likelihood = -21.107167
Iteration 7:   log likelihood = -21.107166

ARIMA regression

Sample:  2012w19 - 2013w36                      Number of obs      =        70
                                                Wald chi2(2)       =      6.75
Log likelihood = -21.10717                      Prob > chi2        =    0.0342

------------------------------------------------------------------------------
             |                 OPG
      D.lnHi |      Coef.   Std. Err.      z    P>|z|     [95% Conf. Interval]
-------------+----------------------------------------------------------------
lnHi         |
       _cons |   -.033687   .0345996    -0.97   0.330     -.101501     .034127
-------------+----------------------------------------------------------------
ARMA         |
          ar |
         L1. |  -.4880343   .4129426    -1.18   0.237    -1.297387    .3213185
             |
          ma |
         L1. |   .2669846   .4758861     0.56   0.575     -.665735    1.199704
-------------+----------------------------------------------------------------
      /sigma |   .3269661   .0317527    10.30   0.000      .264732    .3892003
------------------------------------------------------------------------------
Note: The test of the variance against zero is one sided, and the two-sided confidence interval
is truncated at zero.

.
. estat ic

-----------------------------------------------------------------------------
       Model |    Obs    ll(null)   ll(model)     df          AIC         BIC
-------------+---------------------------------------------------------------
           . |     70           .   -21.10717      4     50.21433    59.20831
-----------------------------------------------------------------------------
               Note:  N=Obs used in calculating BIC; see [R] BIC note

.
. estimates store M7

.
.
.
. arima lnHi L7DWP L7NDVI if tin(2012w19, 2013w36), arima(1,1,1)
```

```
(setting optimization to BHHH)
Iteration 0:   log likelihood =  -15.58843
Iteration 1:   log likelihood = -13.845294
Iteration 2:   log likelihood = -13.628197
Iteration 3:   log likelihood = -13.627994
Iteration 4:   log likelihood = -13.587902
(switching optimization to BFGS)
Iteration 5:   log likelihood = -13.437526
Iteration 6:   log likelihood = -13.423772
Iteration 7:   log likelihood = -13.422658
Iteration 8:   log likelihood = -13.422509
Iteration 9:   log likelihood = -13.422478
Iteration 10:  log likelihood = -13.422458
Iteration 11:  log likelihood = -13.422458

ARIMA regression

Sample:  2012w19 - 2013w36                        Number of obs      =        70
                                                  Wald chi2(4)       =     27.07
Log likelihood = -13.42246                        Prob > chi2        =    0.0000

------------------------------------------------------------------------------
             |                 OPG
      D.lnHi |      Coef.   Std. Err.      z    P>|z|     [95% Conf. Interval]
-------------+----------------------------------------------------------------
lnHi         |
       L7DWP |
         D1. |   .0439282   .0181694     2.42   0.016     .0083169    .0795395
             |
      L7NDVI |
         D1. |    2.41869   1.321015     1.83   0.067    -.1704517    5.007831
             |
       _cons |  -.0152533   .0295543    -0.52   0.606    -.0731786     .042672
-------------+----------------------------------------------------------------
ARMA         |
          ar |
         L1. |  -.4179664   .3009655    -1.39   0.165    -1.007848     .171915
             |
          ma |
         L1. |   .0186104   .3658031     0.05   0.959    -.6983506    .7355714
-------------+----------------------------------------------------------------
      /sigma |   .2927459   .0226054    12.95   0.000     .2484402    .3370517
------------------------------------------------------------------------------
Note: The test of the variance against zero is one sided, and the two-sided confidence interval
is truncated at zero.

.
. estat ic

-----------------------------------------------------------------------------
       Model |    Obs    ll(null)   ll(model)     df          AIC         BIC
-------------+---------------------------------------------------------------
           . |     70           .   -13.42246      6     38.84492    52.33589
-----------------------------------------------------------------------------
               Note:  N=Obs used in calculating BIC; see [R] BIC note

.
. estimates store M101

.
.
.
. arima lnHi L7DWP if tin(2012w19, 2013w36), arima(1,1,1)

(setting optimization to BHHH)
Iteration 0:   log likelihood =  -17.93083
Iteration 1:   log likelihood = -16.578535
Iteration 2:   log likelihood = -16.466147
Iteration 3:   log likelihood = -16.454106
Iteration 4:   log likelihood = -16.425622
```

```
(switching optimization to BFGS)
Iteration 5:   log likelihood = -16.424587
Iteration 6:   log likelihood =  -16.42428
Iteration 7:   log likelihood = -16.424235
Iteration 8:   log likelihood = -16.424235

ARIMA regression

Sample:  2012w19 - 2013w36                      Number of obs      =        70
                                                Wald chi2(3)       =     14.18
Log likelihood = -16.42423                      Prob > chi2        =    0.0027

------------------------------------------------------------------------------
             |                 OPG
      D.lnHi |      Coef.   Std. Err.      z    P>|z|     [95% Conf. Interval]
-------------+----------------------------------------------------------------
lnHi         |
       L7DWP |
         D1. |    .048054   .0183351     2.62   0.009     .0121179    .0839901
             |
       _cons |  -.0247632   .0286251    -0.87   0.387    -.0808674     .031341
-------------+----------------------------------------------------------------
ARMA         |
          ar |
         L1. |  -.3643952   .3575193    -1.02   0.308     -1.06512    .3363298
             |
          ma |
         L1. |   .0340069   .4052951     0.08   0.933    -.7603569    .8283707
-------------+----------------------------------------------------------------
      /sigma |   .3056916   .0257309    11.88   0.000     .2552599    .3561233
------------------------------------------------------------------------------
Note: The test of the variance against zero is one sided, and the two-sided confidence interval
is truncated at zero.

.
. estat ic

-----------------------------------------------------------------------------
       Model |    Obs    ll(null)   ll(model)     df          AIC         BIC
-------------+---------------------------------------------------------------
           . |     70           .   -16.42423      5     42.84847    54.09095
-----------------------------------------------------------------------------
               Note:  N=Obs used in calculating BIC; see [R] BIC note

.
. estimates store M102

.
.
.
. arima lnHi L7LST L7NDVI if tin(2012w19, 2013w36), arima(1,1,1)

(setting optimization to BHHH)
Iteration 0:   log likelihood = -13.815654
Iteration 1:   log likelihood = -12.731133
Iteration 2:   log likelihood = -11.513754
Iteration 3:   log likelihood = -11.490257
Iteration 4:   log likelihood = -11.477087
(switching optimization to BFGS)
Iteration 5:   log likelihood = -11.476879
Iteration 6:   log likelihood = -11.476568
Iteration 7:   log likelihood = -11.476554
Iteration 8:   log likelihood = -11.476545
Iteration 9:   log likelihood = -11.476545

ARIMA regression

Sample:  2012w19 - 2013w36                      Number of obs      =        70
                                                Wald chi2(4)       =     32.35
Log likelihood = -11.47655                      Prob > chi2        =    0.0000
```

```
------------------------------------------------------------------------------
             |                 OPG
     D.lnHi |      Coef.   Std. Err.      z    P>|z|     [95% Conf. Interval]
-------------+----------------------------------------------------------------
lnHi         |
      L7LST |
        D1. |   .1116425    .024086     4.64   0.000     .0644349    .1588501
             |
     L7NDVI |
        D1. |   2.187114   1.369869     1.60   0.110    -.4977796    4.872007
             |
       _cons |  -.0177045   .0268065    -0.66   0.509    -.0702442    .0348353
-------------+----------------------------------------------------------------
ARMA         |
          ar |
         L1. |  -.4805499   .2554347    -1.88   0.060    -.9811928    .0200929
             |
          ma |
         L1. |   .0831911   .3110307     0.27   0.789    -.5264178       .6928
-------------+----------------------------------------------------------------
      /sigma |   .2846963   .0287175     9.91   0.000      .228411    .3409816
------------------------------------------------------------------------------
Note: The test of the variance against zero is one sided, and the two-sided confidence interval
is truncated at zero.

.
. estat ic

-----------------------------------------------------------------------------
       Model |    Obs    ll(null)   ll(model)     df          AIC         BIC
-------------+---------------------------------------------------------------
           . |     70           .   -11.47655      6     34.95309    48.44406
-----------------------------------------------------------------------------
               Note:  N=Obs used in calculating BIC; see [R] BIC note

.
. estimates store M103

.
.
.
.
.
.
.
. estimates stats M1 M2 M3 M4 M5 M6 M7 M101 M102 M103

-----------------------------------------------------------------------------
       Model |    Obs    ll(null)   ll(model)     df          AIC         BIC
-------------+---------------------------------------------------------------
          M1 |     70           .   -7.168514     10     34.33703    56.82198
          M2 |     70           .   -7.355228      9     32.71046    52.94691
          M3 |     70           .   -7.716914      8     31.43383    49.42179
          M4 |     70           .   -8.655586      7     31.31117    47.05064
          M5 |     70           .   -10.88849      6     33.77699    47.26796
          M6 |     70           .   -13.99401      5     37.98802     49.2305
          M7 |     70           .   -21.10717      4     50.21433    59.20831
        M101 |     70           .   -13.42246      6     38.84492    52.33589
        M102 |     70           .   -16.42423      5     42.84847    54.09095
        M103 |     70           .   -11.47655      6     34.95309    48.44406
-----------------------------------------------------------------------------
               Note:  N=Obs used in calculating BIC; see [R] BIC note

.
.
.
. * the next step is to further evaluate M3 and M4, due to them having the lowest AIC-values.

.
. * Here we perform modeling of these with different ARIMA-parameters, thereafter again looking
```

```
.
. * at an overview and choosing the best model.

.
. arima lnHi L7DWP L7LST L7NDVI if tin(2012w19, 2013w36), arima(1,1,1)

(setting optimization to BHHH)
Iteration 0:   log likelihood = -9.7522477
Iteration 1:   log likelihood = -9.0313967
Iteration 2:   log likelihood = -8.7719251
Iteration 3:   log likelihood = -8.7102241
Iteration 4:   log likelihood = -8.7059065
(switching optimization to BFGS)
Iteration 5:   log likelihood = -8.6825257
Iteration 6:   log likelihood = -8.6558003
Iteration 7:   log likelihood = -8.6556826
Iteration 8:   log likelihood = -8.6555932
Iteration 9:   log likelihood = -8.6555869
Iteration 10:  log likelihood = -8.6555858
Iteration 11:  log likelihood = -8.6555857

ARIMA regression

Sample:  2012w19 - 2013w36                        Number of obs      =        70
                                                  Wald chi2(5)       =     41.82
Log likelihood = -8.655586                        Prob > chi2        =    0.0000

------------------------------------------------------------------------------
             |                 OPG
      D.lnHi |      Coef.   Std. Err.      z    P>|z|     [95% Conf. Interval]
-------------+----------------------------------------------------------------
lnHi         |
       L7DWP |
         D1. |   .0323079     .01793     1.80   0.072    -.0028342      .06745
             |
       L7LST |
         D1. |   .0929454   .0253781     3.66   0.000     .0432053    .1426855
             |
      L7NDVI |
         D1. |   1.960931   1.324157     1.48   0.139    -.6343693    4.556231
             |
       _cons |  -.0137697   .0266529    -0.52   0.605    -.0660084     .038469
-------------+----------------------------------------------------------------
ARMA         |
          ar |
         L1. |  -.4649715   .2826193    -1.65   0.100    -1.018895    .0889521
             |
          ma |
         L1. |   .0504677    .361023     0.14   0.889    -.6571243    .7580597
-------------+----------------------------------------------------------------
      /sigma |   .2734307   .0260862    10.48   0.000     .2223027    .3245587
------------------------------------------------------------------------------
Note: The test of the variance against zero is one sided, and the two-sided confidence interval
is truncated at zero.

.
. estat ic

-----------------------------------------------------------------------------
       Model |    Obs    ll(null)   ll(model)     df          AIC         BIC
-------------+---------------------------------------------------------------
           . |     70           .   -8.655586      7     31.31117    47.05064
-----------------------------------------------------------------------------
               Note:  N=Obs used in calculating BIC; see [R] BIC note

.
. estimates store M8

.
.
.
```

```
. arima lnHi L7DWP L7LST L7NDVI if tin(2012w19, 2013w36), arima(2,1,1)

(setting optimization to BHHH)
Iteration 0:   log likelihood =   -10.3389
Iteration 1:   log likelihood = -9.3532444
Iteration 2:   log likelihood = -8.8555994
Iteration 3:   log likelihood = -8.5710963
Iteration 4:   log likelihood = -8.3680095
(switching optimization to BFGS)
Iteration 5:   log likelihood = -8.3051507
Iteration 6:   log likelihood = -8.0178239
Iteration 7:   log likelihood = -7.9551909
Iteration 8:   log likelihood = -7.9491677
Iteration 9:   log likelihood = -7.9485272
Iteration 10:  log likelihood = -7.9480986
Iteration 11:  log likelihood = -7.9480278
Iteration 12:  log likelihood = -7.9479546
Iteration 13:  log likelihood = -7.9479522
Iteration 14:  log likelihood = -7.9479505
(switching optimization to BHHH)
Iteration 15:  log likelihood = -7.9479504
Iteration 16:  log likelihood = -7.9479504  (backed up)
Iteration 17:  log likelihood = -7.9479504  (not concave)
Iteration 18:  log likelihood = -7.9479504
Iteration 19:  log likelihood = -7.9479504  (backed up)
(switching optimization to BFGS)
Iteration 20:  log likelihood = -7.9479504

ARIMA regression

Sample:  2012w19 - 2013w36                      Number of obs      =        70
                                                Wald chi2(6)       =    573.60
Log likelihood =  -7.94795                      Prob > chi2        =    0.0000

------------------------------------------------------------------------------
             |                 OPG
      D.lnHi |      Coef.   Std. Err.      z    P>|z|     [95% Conf. Interval]
-------------+----------------------------------------------------------------
lnHi         |
       L7DWP |
         D1. |   .0288628   .0159798     1.81   0.071    -.0024571    .0601827
             |
       L7LST |
         D1. |   .0983511   .0256379     3.84   0.000     .0481017    .1486005
             |
      L7NDVI |
         D1. |   1.814922   1.331916     1.36   0.173    -.7955845    4.425429
             |
       _cons |  -.0146129   .0268041    -0.55   0.586     -.067148    .0379222
-------------+----------------------------------------------------------------
ARMA         |
          ar |
         L1. |  -1.395268   .1244441   -11.21   0.000    -1.639174   -1.151362
         L2. |  -.4495805   .1279622    -3.51   0.000    -.7003819   -.1987791
             |
          ma |
         L1. |   .9999995   3869.844     0.00   1.000    -7583.754    7585.754
-------------+----------------------------------------------------------------
      /sigma |   .2677417   518.0446     0.00   0.500            0    1015.617
------------------------------------------------------------------------------
Note: The test of the variance against zero is one sided, and the two-sided confidence interval
is truncated at zero.

.
. estat ic

-----------------------------------------------------------------------------
       Model |    Obs    ll(null)   ll(model)     df          AIC         BIC
-------------+---------------------------------------------------------------
           . |     70           .    -7.94795      8      31.8959    49.88386
-----------------------------------------------------------------------------
```

```
               Note:  N=Obs used in calculating BIC; see [R] BIC note

.
. estimates store M9

.
.
.
. arima lnHi L7DWP L7LST L7NDVI if tin(2012w19, 2013w36), arima(1,1,2)

(setting optimization to BHHH)
Iteration 0:   log likelihood = -8.8282908
Iteration 1:   log likelihood = -7.7223218
Iteration 2:   log likelihood = -7.5110544
Iteration 3:   log likelihood = -7.4622259
Iteration 4:   log likelihood = -7.3925485
(switching optimization to BFGS)
Iteration 5:   log likelihood = -7.3866382
Iteration 6:   log likelihood = -7.2243717
Iteration 7:   log likelihood = -7.2086414
Iteration 8:   log likelihood = -7.2064282
Iteration 9:   log likelihood = -7.2041966
Iteration 10:  log likelihood = -7.2038888
Iteration 11:  log likelihood = -7.2038337
Iteration 12:  log likelihood = -7.2038288
Iteration 13:  log likelihood = -7.2038283

ARIMA regression

Sample:  2012w19 - 2013w36                      Number of obs      =        70
                                                Wald chi2(6)       =     46.38
Log likelihood = -7.203828                      Prob > chi2        =    0.0000

------------------------------------------------------------------------------
             |                 OPG
      D.lnHi |      Coef.   Std. Err.      z    P>|z|     [95% Conf. Interval]
-------------+----------------------------------------------------------------
lnHi         |
       L7DWP |
         D1. |   .0345842   .0156407     2.21   0.027     .0039291    .0652394
             |
       L7LST |
         D1. |   .1037044    .027102     3.83   0.000     .0505854    .1568234
             |
      L7NDVI |
         D1. |   1.455246   1.175361     1.24   0.216    -.8484194    3.758911
             |
       _cons |  -.0136808   .0368981    -0.37   0.711    -.0859997    .0586381
-------------+----------------------------------------------------------------
ARMA         |
          ar |
         L1. |   .0423816   .4368381     0.10   0.923    -.8138054    .8985686
             |
          ma |
         L1. |  -.4547861   .4207564    -1.08   0.280    -1.279454    .3698813
         L2. |   .3788203    .157377     2.41   0.016      .070367    .6872735
-------------+----------------------------------------------------------------
      /sigma |   .2674568   .0276677     9.67   0.000      .213229    .3216845
------------------------------------------------------------------------------
Note: The test of the variance against zero is one sided, and the two-sided confidence interval
is truncated at zero.

.
. estat ic

-----------------------------------------------------------------------------
       Model |    Obs    ll(null)   ll(model)     df          AIC         BIC
-------------+---------------------------------------------------------------
           . |     70           .   -7.203828      8     30.40766    48.39562
-----------------------------------------------------------------------------
               Note:  N=Obs used in calculating BIC; see [R] BIC note
```

```
.
. estimates store M10

.
.
.
. arima lnHi L7DWP L7LST L7NDVI if tin(2012w19, 2013w36), arima(3,1,1)

(setting optimization to BHHH)
Iteration 0:   log likelihood = -9.8947282
Iteration 1:   log likelihood = -7.9661504
Iteration 2:   log likelihood = -7.9512108
Iteration 3:   log likelihood = -7.9031224
Iteration 4:   log likelihood = -7.7439446
(switching optimization to BFGS)
Iteration 5:   log likelihood = -7.7340261
Iteration 6:   log likelihood = -7.7332886
Iteration 7:   log likelihood = -7.7292862
Iteration 8:   log likelihood = -7.7272856
Iteration 9:   log likelihood = -7.7259551
Iteration 10:  log likelihood = -7.7259339
Iteration 11:  log likelihood = -7.7259325
Iteration 12:  log likelihood = -7.7259321

ARIMA regression

Sample:  2012w19 - 2013w36                      Number of obs      =        70
                                                Wald chi2(7)       =     46.09
Log likelihood = -7.725932                      Prob > chi2        =    0.0000

------------------------------------------------------------------------------
             |                 OPG
      D.lnHi |      Coef.   Std. Err.      z    P>|z|     [95% Conf. Interval]
-------------+----------------------------------------------------------------
lnHi         |
       L7DWP |
         D1. |   .0345932   .0176804     1.96   0.050    -.0000598    .0692462
             |
       L7LST |
         D1. |   .1002924   .0267256     3.75   0.000     .0479112    .1526735
             |
      L7NDVI |
         D1. |   1.636917    1.24242     1.32   0.188    -.7981826    4.072016
             |
       _cons |  -.0139367   .0340507    -0.41   0.682    -.0806747    .0528014
-------------+----------------------------------------------------------------
ARMA         |
          ar |
         L1. |  -.3819334   .9156339    -0.42   0.677    -2.176543    1.412676
         L2. |   .1164578   .3879549     0.30   0.764    -.6439197    .8768354
         L3. |   .1705258   .1185227     1.44   0.150    -.0617745    .4028261
             |
          ma |
         L1. |  -.0254976   .9083686    -0.03   0.978    -1.805867    1.754872
-------------+----------------------------------------------------------------
      /sigma |   .2696637     .02742     9.83   0.000     .2159215    .3234059
------------------------------------------------------------------------------
Note: The test of the variance against zero is one sided, and the two-sided confidence interval
is truncated at zero.

.
. estat ic

-----------------------------------------------------------------------------
       Model |    Obs    ll(null)   ll(model)     df          AIC         BIC
-------------+---------------------------------------------------------------
           . |     70           .   -7.725932      9     33.45186    53.68832
-----------------------------------------------------------------------------
               Note:  N=Obs used in calculating BIC; see [R] BIC note
```

```
.
. estimates store M11

.
.
.
. arima lnHi L7DWP L7LST L7NDVI if tin(2012w19, 2013w36), arima(1,1,3)

(setting optimization to BHHH)
Iteration 0:   log likelihood = -9.0124757
Iteration 1:   log likelihood = -7.6920569
Iteration 2:   log likelihood = -7.1923682
Iteration 3:   log likelihood =  -7.156605
Iteration 4:   log likelihood = -6.7850828
(switching optimization to BFGS)
Iteration 5:   log likelihood = -6.6404728
Iteration 6:   log likelihood = -6.4705557
Iteration 7:   log likelihood = -6.3715012
Iteration 8:   log likelihood = -6.3343358
Iteration 9:   log likelihood = -6.3262272
Iteration 10:  log likelihood = -6.3213961
Iteration 11:  log likelihood =  -6.320881
Iteration 12:  log likelihood = -6.3207013
Iteration 13:  log likelihood = -6.3206908
Iteration 14:  log likelihood = -6.3206901
(switching optimization to BHHH)
Iteration 15:  log likelihood =   -6.32069
Iteration 16:  log likelihood =   -6.32069  (not concave)
Iteration 17:  log likelihood =   -6.32069
Iteration 18:  log likelihood =   -6.32069  (not concave)
Iteration 19:  log likelihood =   -6.32069
(switching optimization to BFGS)
Iteration 20:  log likelihood =   -6.32069

ARIMA regression

Sample:  2012w19 - 2013w36                      Number of obs      =        70
                                                Wald chi2(6)       =    168.54
Log likelihood =  -6.32069                      Prob > chi2        =    0.0000

------------------------------------------------------------------------------
             |                 OPG
      D.lnHi |      Coef.   Std. Err.      z    P>|z|     [95% Conf. Interval]
-------------+----------------------------------------------------------------
lnHi         |
       L7DWP |
         D1. |   .0317592   .0149325     2.13   0.033     .0024921    .0610264
             |
       L7LST |
         D1. |   .1101422   .0278721     3.95   0.000     .0555139    .1647705
             |
      L7NDVI |
         D1. |   1.155587   1.214763     0.95   0.341    -1.225305     3.53648
             |
       _cons |  -.0145485   .0397178    -0.37   0.714    -.0923939     .063297
-------------+----------------------------------------------------------------
ARMA         |
          ar |
         L1. |  -.8914096   .0922778    -9.66   0.000    -1.072271   -.7105485
             |
          ma |
         L1. |   .5483937          .        .       .            .           .
         L2. |  -.0278404   .2268625    -0.12   0.902    -.4724827    .4168019
         L3. |   .4237658   .1728267     2.45   0.014     .0850316    .7624999
-------------+----------------------------------------------------------------
      /sigma |   .2611169   .0355956     7.34   0.000     .1913508    .3308831
------------------------------------------------------------------------------
Note: The test of the variance against zero is one sided, and the two-sided confidence interval
is truncated at zero.

.
```

```
. estat ic

-----------------------------------------------------------------------------
       Model |    Obs    ll(null)   ll(model)     df          AIC         BIC
-------------+---------------------------------------------------------------
           . |     70           .    -6.32069      8     28.64138    46.62934
-----------------------------------------------------------------------------
               Note:  N=Obs used in calculating BIC; see [R] BIC note

.
. estimates store M12

.
.
.
. arima lnHi L7DWP L7LST L7NDVI if tin(2012w19, 2013w36), arima(3,1,2)

(setting optimization to BHHH)
Iteration 0:   log likelihood = -8.6528383
Iteration 1:   log likelihood = -7.0150709
Iteration 2:   log likelihood =  -5.015495
Iteration 3:   log likelihood = -3.9813145
Iteration 4:   log likelihood = -3.0855802
(switching optimization to BFGS)
Iteration 5:   log likelihood = -2.9508502
Iteration 6:   log likelihood = -2.8920121
Iteration 7:   log likelihood =  -2.831044
Iteration 8:   log likelihood =  -2.788252
Iteration 9:   log likelihood = -2.7751375
Iteration 10:  log likelihood = -2.7676815
Iteration 11:  log likelihood = -2.7671597
Iteration 12:  log likelihood = -2.7670839
Iteration 13:  log likelihood = -2.7670828
Iteration 14:  log likelihood = -2.7670819
(switching optimization to BHHH)
Iteration 15:  log likelihood = -2.7670818
Iteration 16:  log likelihood = -2.7670818  (not concave)
Iteration 17:  log likelihood = -2.7670818
Iteration 18:  log likelihood = -2.7670818  (not concave)
Iteration 19:  log likelihood = -2.7670818  (not concave)
(switching optimization to BFGS)
Iteration 20:  log likelihood = -2.7670818

ARIMA regression

Sample:  2012w19 - 2013w36                      Number of obs      =        70
                                                Wald chi2(7)       =    193.48
Log likelihood = -2.767082                      Prob > chi2        =    0.0000

------------------------------------------------------------------------------
             |                 OPG
      D.lnHi |      Coef.   Std. Err.      z    P>|z|     [95% Conf. Interval]
-------------+----------------------------------------------------------------
lnHi         |
       L7DWP |
         D1. |   .0348577   .0096329     3.62   0.000     .0159775    .0537379
             |
       L7LST |
         D1. |   .0769016   .0237688     3.24   0.001     .0303156    .1234876
             |
      L7NDVI |
         D1. |   .8263544   .8516829     0.97   0.332    -.8429134    2.495622
             |
       _cons |  -.0172087   .0325314    -0.53   0.597     -.080969    .0465517
-------------+----------------------------------------------------------------
ARMA         |
          ar |
         L1. |  -.1348012   .1681095    -0.80   0.423    -.4642897    .1946873
         L2. |  -.5763686   .1472674    -3.91   0.000    -.8650074   -.2877298
         L3. |  -.0719062   .1325071    -0.54   0.587    -.3316154    .1878029
             |
```

```
          ma |
         L1. |  -.2892138   .1261007    -2.29   0.022    -.5363667   -.0420609
         L2. |   .9999992          .        .       .            .           .
-------------+----------------------------------------------------------------
      /sigma |   .2424099   .0252213     9.61   0.000     .1929769    .2918428
------------------------------------------------------------------------------
Note: The test of the variance against zero is one sided, and the two-sided confidence interval
is truncated at zero.

.
. estat ic

-----------------------------------------------------------------------------
       Model |    Obs    ll(null)   ll(model)     df          AIC         BIC
-------------+---------------------------------------------------------------
           . |     70           .   -2.767082      9     23.53416    43.77062
-----------------------------------------------------------------------------
               Note:  N=Obs used in calculating BIC; see [R] BIC note

.
. estimates store M13

.
.
.
. arima lnHi L7DWP L7LST L7NDVI if tin(2012w19, 2013w36), arima(2,1,3)

(setting optimization to BHHH)
Iteration 0:   log likelihood = -8.0199815
Iteration 1:   log likelihood = -7.9145683
Iteration 2:   log likelihood = -5.1392213
Iteration 3:   log likelihood = -4.4336346
Iteration 4:   log likelihood =  -3.578524
(switching optimization to BFGS)
Iteration 5:   log likelihood = -3.3759562
Iteration 6:   log likelihood = -3.0840791
Iteration 7:   log likelihood = -2.9784461
Iteration 8:   log likelihood = -2.8304522
Iteration 9:   log likelihood = -2.8060936
Iteration 10:  log likelihood = -2.7941439
Iteration 11:  log likelihood = -2.7927727
Iteration 12:  log likelihood = -2.7923574
Iteration 13:  log likelihood = -2.7923339
Iteration 14:  log likelihood = -2.7923294
(switching optimization to BHHH)
Iteration 15:  log likelihood = -2.7923285  (not concave)
Iteration 16:  log likelihood = -2.7923284
Iteration 17:  log likelihood = -2.7923284  (not concave)
Iteration 18:  log likelihood = -2.7923284
Iteration 19:  log likelihood = -2.7923284  (not concave)
(switching optimization to BFGS)
Iteration 20:  log likelihood = -2.7923284

ARIMA regression

Sample:  2012w19 - 2013w36                      Number of obs      =        70
                                                Wald chi2(8)       =    166.96
Log likelihood = -2.792328                      Prob > chi2        =    0.0000

------------------------------------------------------------------------------
             |                 OPG
      D.lnHi |      Coef.   Std. Err.      z    P>|z|     [95% Conf. Interval]
-------------+----------------------------------------------------------------
lnHi         |
       L7DWP |
         D1. |   .0349159   .0103958     3.36   0.001     .0145406    .0552912
             |
       L7LST |
         D1. |    .076839   .0242775     3.17   0.002     .0292559     .124422
             |
      L7NDVI |
```

```
         D1. |   .8044022   .8430883     0.95   0.340    -.8480205    2.456825
             |
       _cons |  -.0172645   .0327839    -0.53   0.598    -.0815198    .0469907
-------------+----------------------------------------------------------------
ARMA         |
          ar |
         L1. |  -.0193171   .2970429    -0.07   0.948    -.6015105    .5628764
         L2. |  -.5627712   .1675782    -3.36   0.001    -.8912184   -.2343239
             |
          ma |
         L1. |  -.3991312   205.9648    -0.00   0.998    -404.0828    403.2846
         L2. |   1.031316   1434.685     0.00   0.999      -2810.9    2812.963
         L3. |  -.1072889   151.3947    -0.00   0.999    -296.8354    296.6208
-------------+----------------------------------------------------------------
      /sigma |   .2424148   171.2294     0.00   0.499            0    335.8458
------------------------------------------------------------------------------
Note: The test of the variance against zero is one sided, and the two-sided confidence interval
is truncated at zero.

.
. estat ic

-----------------------------------------------------------------------------
       Model |    Obs    ll(null)   ll(model)     df          AIC         BIC
-------------+---------------------------------------------------------------
           . |     70           .   -2.792328     10     25.58466    48.06961
-----------------------------------------------------------------------------
               Note:  N=Obs used in calculating BIC; see [R] BIC note

.
. estimates store M14

.
.
.
. arima lnHi L7DWP L7LST L7NDVI if tin(2012w19, 2013w36), arima(3,1,3)

(setting optimization to BHHH)
Iteration 0:   log likelihood = -8.6164103
Iteration 1:   log likelihood = -5.9962298
Iteration 2:   log likelihood = -5.3176578
Iteration 3:   log likelihood =  -5.011704
Iteration 4:   log likelihood = -4.8691457
(switching optimization to BFGS)
Iteration 5:   log likelihood = -3.7712148
Iteration 6:   log likelihood = -3.6981822
Iteration 7:   log likelihood = -3.6091857
Iteration 8:   log likelihood = -3.1931136
Iteration 9:   log likelihood = -3.0671351
Iteration 10:  log likelihood =  -3.040837
Iteration 11:  log likelihood = -3.0315237
Iteration 12:  log likelihood = -3.0242991
Iteration 13:  log likelihood =  -2.731562
Iteration 14:  log likelihood = -2.7181047
(switching optimization to BHHH)
Iteration 15:  log likelihood = -2.6839524
Iteration 16:  log likelihood = -2.6804678
Iteration 17:  log likelihood = -2.6795559  (backed up)
Iteration 18:  log likelihood = -2.6794514  (backed up)
Iteration 19:  log likelihood = -2.6793132  (backed up)
(switching optimization to BFGS)
Iteration 20:  log likelihood = -2.6793074  (backed up)
Iteration 21:  log likelihood = -2.6782521
Iteration 22:  log likelihood = -2.6753716
Iteration 23:  log likelihood = -2.6728943
Iteration 24:  log likelihood = -2.6711223
Iteration 25:  log likelihood = -2.6703488
Iteration 26:  log likelihood =  -2.670318
Iteration 27:  log likelihood =  -2.670309
Iteration 28:  log likelihood = -2.6703087
```

```
ARIMA regression

Sample:  2012w19 - 2013w36                      Number of obs      =        70
                                                Wald chi2(8)       =    168.32
Log likelihood = -2.670309                      Prob > chi2        =    0.0000

------------------------------------------------------------------------------
             |                 OPG
      D.lnHi |      Coef.   Std. Err.      z    P>|z|     [95% Conf. Interval]
-------------+----------------------------------------------------------------
lnHi         |
       L7DWP |
         D1. |   .0329735   .0095359     3.46   0.001     .0142836    .0516635
             |
       L7LST |
         D1. |   .0822982   .0247753     3.32   0.001     .0337395    .1308569
             |
      L7NDVI |
         D1. |   .5561476   .8133168     0.68   0.494    -1.037924    2.150219
             |
       _cons |  -.0180114   .0358244    -0.50   0.615     -.088226    .0522031
-------------+----------------------------------------------------------------
ARMA         |
          ar |
         L1. |  -1.031663    .184019    -5.61   0.000    -1.392334   -.6709928
         L2. |  -.6238293   .2429746    -2.57   0.010    -1.100051   -.1476079
         L3. |  -.5086514   .1744341    -2.92   0.004    -.8505359   -.1667668
             |
          ma |
         L1. |   .7009178   .2783042     2.52   0.012     .1554515    1.246384
         L2. |   .6902129   .2758946     2.50   0.012     .1494694    1.230956
         L3. |   1.008189          .        .       .            .           .
-------------+----------------------------------------------------------------
      /sigma |   .2384445   .0282472     8.44   0.000      .183081    .2938081
------------------------------------------------------------------------------
Note: The test of the variance against zero is one sided, and the two-sided confidence interval
is truncated at zero.

.
. estat ic

-----------------------------------------------------------------------------
       Model |    Obs    ll(null)   ll(model)     df          AIC         BIC
-------------+---------------------------------------------------------------
           . |     70           .   -2.670309     10     25.34062    47.82557
-----------------------------------------------------------------------------
               Note:  N=Obs used in calculating BIC; see [R] BIC note

.
. estimates store M15

.
.
.
. arima lnHi L7DWP L7LST L7NDVI if tin(2012w19, 2013w36), arima(4,1,3)

(setting optimization to BHHH)
Iteration 0:   log likelihood =  -8.949288
Iteration 1:   log likelihood = -7.2044002
Iteration 2:   log likelihood = -4.8597779
Iteration 3:   log likelihood = -4.4064908
Iteration 4:   log likelihood = -3.9327419
(switching optimization to BFGS)
Iteration 5:   log likelihood = -3.9194041
Iteration 6:   log likelihood = -3.7087738
Iteration 7:   log likelihood =  -2.732742
Iteration 8:   log likelihood = -2.6252262
Iteration 9:   log likelihood = -2.5779896
Iteration 10:  log likelihood = -2.4880842
Iteration 11:  log likelihood = -2.3682911
Iteration 12:  log likelihood = -2.3226854
```

```
Iteration 13:  log likelihood = -2.3126716
Iteration 14:  log likelihood =  -2.310756
(switching optimization to BHHH)
Iteration 15:  log likelihood = -2.3098801
Iteration 16:  log likelihood = -2.3098793  (backed up)
Iteration 17:  log likelihood = -2.3098791  (backed up)
Iteration 18:  log likelihood = -2.3098786  (backed up)
Iteration 19:  log likelihood = -2.3098786  (backed up)
(switching optimization to BFGS)
Iteration 20:  log likelihood = -2.3098786  (backed up)
Iteration 21:  log likelihood = -2.3097655
Iteration 22:  log likelihood =  -2.309695
Iteration 23:  log likelihood = -2.3096902
Iteration 24:  log likelihood = -2.3096118
Iteration 25:  log likelihood = -2.3096052
Iteration 26:  log likelihood = -2.3095915
Iteration 27:  log likelihood = -2.3095913

ARIMA regression

Sample:  2012w19 - 2013w36                        Number of obs      =        70
                                                  Wald chi2(9)       =    153.60
Log likelihood = -2.309591                        Prob > chi2        =    0.0000

------------------------------------------------------------------------------
             |                 OPG
      D.lnHi |      Coef.   Std. Err.      z    P>|z|     [95% Conf. Interval]
-------------+----------------------------------------------------------------
lnHi         |
       L7DWP |
         D1. |   .0321944   .0096359     3.34   0.001     .0133083    .0510804
             |
       L7LST |
         D1. |   .0852062   .0253795     3.36   0.001     .0354633     .134949
             |
      L7NDVI |
         D1. |   .6550234   .8970877     0.73   0.465    -1.103236    2.413283
             |
       _cons |    -.01816    .033887    -0.54   0.592    -.0845773    .0482572
-------------+----------------------------------------------------------------
ARMA         |
          ar |
         L1. |  -1.074796   .1888255    -5.69   0.000    -1.444887   -.7047045
         L2. |   -.692571   .2729863    -2.54   0.011    -1.227614   -.1575276
         L3. |  -.6216079   .2113414    -2.94   0.003    -1.035829   -.2073863
         L4. |  -.1127244   .1497059    -0.75   0.451    -.4061425    .1806936
             |
          ma |
         L1. |   .6923859   277.1136     0.00   0.998    -542.4404    543.8251
         L2. |   .6923966   277.1392     0.00   0.998    -542.4905    543.8752
         L3. |   .9999917          .        .       .            .           .
-------------+----------------------------------------------------------------
      /sigma |   .2386096   .0294169     8.11   0.000     .1809535    .2962658
------------------------------------------------------------------------------
Note: The test of the variance against zero is one sided, and the two-sided confidence interval
is truncated at zero.

.
. estat ic

-----------------------------------------------------------------------------
       Model |    Obs    ll(null)   ll(model)     df          AIC         BIC
-------------+---------------------------------------------------------------
           . |     70           .   -2.309591     11     26.61918    51.35263
-----------------------------------------------------------------------------
               Note:  N=Obs used in calculating BIC; see [R] BIC note

.
. estimates store M16

.
```

```
.
.
. arima lnHi L7DWP L7LST L7NDVI if tin(2012w19, 2013w36), arima(3,1,4)

(setting optimization to BHHH)
Iteration 0:   log likelihood = -9.3827505
Iteration 1:   log likelihood = -9.1189029
Iteration 2:   log likelihood = -8.0685974
Iteration 3:   log likelihood = -8.0377924
Iteration 4:   log likelihood = -7.4603563
(switching optimization to BFGS)
Iteration 5:   log likelihood = -4.3254709
Iteration 6:   log likelihood = -3.9883552
Iteration 7:   log likelihood = -3.9393892
Iteration 8:   log likelihood = -3.1549791
Iteration 9:   log likelihood = -2.9573042
Iteration 10:  log likelihood = -2.3665009
Iteration 11:  log likelihood = -1.8867526
Iteration 12:  log likelihood = -1.7922045
Iteration 13:  log likelihood = -1.7311258
Iteration 14:  log likelihood = -1.5885524
(switching optimization to BHHH)
Iteration 15:  log likelihood = -1.4741346
Iteration 16:  log likelihood = -1.3672796
Iteration 17:  log likelihood = -1.1883708
Iteration 18:  log likelihood = -1.0896302
Iteration 19:  log likelihood = -.95133313
(switching optimization to BFGS)
Iteration 20:  log likelihood = -.56232136
Iteration 21:  log likelihood = -.34832387
Iteration 22:  log likelihood = -.11111049
Iteration 23:  log likelihood = -.08789023
Iteration 24:  log likelihood =  .22166144
Iteration 25:  log likelihood =  .33353928
Iteration 26:  log likelihood =  .39507182
Iteration 27:  log likelihood =  .44010958
Iteration 28:  log likelihood =  .45838253
Iteration 29:  log likelihood =  .46087788
(switching optimization to BHHH)
Iteration 30:  log likelihood =  .46529517
Iteration 31:  log likelihood =  .46598436  (backed up)
Iteration 32:  log likelihood =  .46598683  (backed up)
Iteration 33:  log likelihood =   .4659948  (backed up)
Iteration 34:  log likelihood =  .46599507  (backed up)
(switching optimization to BFGS)
Iteration 35:  log likelihood =   .4659954  (backed up)
Iteration 36:  log likelihood =   .4674284
Iteration 37:  log likelihood =  .46762752
Iteration 38:  log likelihood =  .46789905
Iteration 39:  log likelihood =  .46797455
Iteration 40:  log likelihood =  .46803834
Iteration 41:  log likelihood =   .4680605
Iteration 42:  log likelihood =  .46807355
Iteration 43:  log likelihood =  .46807411
Iteration 44:  log likelihood =  .46807872
(switching optimization to BHHH)
Iteration 45:  log likelihood =  .46807887  (not concave)
Iteration 46:  log likelihood =  .46807894  (not concave)
Iteration 47:  log likelihood =  .46807895  (not concave)
Iteration 48:  log likelihood =  .46807896  (not concave)
Iteration 49:  log likelihood =  .46807896  (not concave)
(switching optimization to BFGS)
Iteration 50:  log likelihood =  .46807896
Iteration 51:  log likelihood =  .46807896

ARIMA regression

Sample:  2012w19 - 2013w36                      Number of obs      =        70
                                                Wald chi2(9)       =  1.65e+06
Log likelihood =   .468079                      Prob > chi2        =    0.0000
```

```
------------------------------------------------------------------------------
             |                 OPG
     D.lnHi |      Coef.   Std. Err.      z    P>|z|     [95% Conf. Interval]
-------------+----------------------------------------------------------------
lnHi         |
       L7DWP |
         D1. |    .034341   .0104383     3.29   0.001     .0138823    .0547996
             |
       L7LST |
         D1. |   .0906228    .025188     3.60   0.000     .0412553    .1399903
             |
      L7NDVI |
         D1. |   1.147173   .6944325     1.65   0.099    -.2138892    2.508236
             |
       _cons |  -.0110905   .0415753    -0.27   0.790    -.0925766    .0703957
-------------+----------------------------------------------------------------
ARMA         |
          ar |
         L1. |  -.5645081   .2146319    -2.63   0.009    -.9851789   -.1438372
         L2. |  -1.073145   .0758612   -14.15   0.000     -1.22183   -.9244596
         L3. |  -.2058557   .2080339    -0.99   0.322    -.6135946    .2018832
             |
          ma |
         L1. |   .2389678   .1924707     1.24   0.214    -.1382677    .6162034
         L2. |   1.660302          .        .       .            .           .
         L3. |   .1293803   .1883829     0.69   0.492    -.2398434    .4986039
         L4. |   .7197922   .0816351     8.82   0.000     .5597904    .8797941
-------------+----------------------------------------------------------------
      /sigma |   .2268819   .0254074     8.93   0.000     .1770844    .2766794
------------------------------------------------------------------------------
Note: The test of the variance against zero is one sided, and the two-sided confidence interval
is truncated at zero.

.
. estat ic

-----------------------------------------------------------------------------
       Model |    Obs    ll(null)   ll(model)     df          AIC         BIC
-------------+---------------------------------------------------------------
           . |     70           .     .468079     11     21.06384    45.79729
-----------------------------------------------------------------------------
               Note:  N=Obs used in calculating BIC; see [R] BIC note

.
. estimates store M17

.
.
.
. arima lnHi L7DWP L7LST L7NDVI if tin(2012w19, 2013w36), arima(4,1,4)

(setting optimization to BHHH)
Iteration 0:   log likelihood = -7.4185338
Iteration 1:   log likelihood = -6.7065407
Iteration 2:   log likelihood = -6.6487751  (backed up)
Iteration 3:   log likelihood =  -5.029474
Iteration 4:   log likelihood = -4.2432919
(switching optimization to BFGS)
Iteration 5:   log likelihood = -3.2087299
Iteration 6:   log likelihood = -3.0656215
Iteration 7:   log likelihood = -1.6122322
Iteration 8:   log likelihood = -1.5177742  (backed up)
Iteration 9:   log likelihood = -1.2153631
Iteration 10:  log likelihood = -.97954177
Iteration 11:  log likelihood = -.91109133
Iteration 12:  log likelihood = -.87299925
Iteration 13:  log likelihood = -.85220552
Iteration 14:  log likelihood = -.83489047
(switching optimization to BHHH)
Iteration 15:  log likelihood = -.81889539
Iteration 16:  log likelihood = -.81886883  (backed up)
```

```
Iteration 17:  log likelihood = -.81883558  (backed up)
Iteration 18:  log likelihood = -.81882925  (backed up)
Iteration 19:  log likelihood = -.81882563  (backed up)
(switching optimization to BFGS)
Iteration 20:  log likelihood = -.81882558  (backed up)
Iteration 21:  log likelihood = -.81641083
Iteration 22:  log likelihood = -.81536936
Iteration 23:  log likelihood = -.81474346
Iteration 24:  log likelihood = -.81445366
Iteration 25:  log likelihood = -.81444099
Iteration 26:  log likelihood = -.81442041
Iteration 27:  log likelihood = -.81441586
Iteration 28:  log likelihood =  -.8144144
Iteration 29:  log likelihood = -.81441431

ARIMA regression

Sample:  2012w19 - 2013w36                      Number of obs      =        70
                                                Wald chi2(10)      =    142.62
Log likelihood = -.8144143                      Prob > chi2        =    0.0000

------------------------------------------------------------------------------
             |                 OPG
      D.lnHi |      Coef.   Std. Err.      z    P>|z|     [95% Conf. Interval]
-------------+----------------------------------------------------------------
lnHi         |
       L7DWP |
         D1. |   .0354344   .0109965     3.22   0.001     .0138817     .056987
             |
       L7LST |
         D1. |     .08113   .0240324     3.38   0.001     .0340273    .1282327
             |
      L7NDVI |
         D1. |   .8966042   .7538393     1.19   0.234    -.5808937    2.374102
             |
       _cons |  -.0105454   .0093561    -1.13   0.260     -.028883    .0077923
-------------+----------------------------------------------------------------
ARMA         |
          ar |
         L1. |  -.1652131   .2014606    -0.82   0.412    -.5600686    .2296424
         L2. |   .2569096   .2240266     1.15   0.251    -.1821745    .6959936
         L3. |  -.0211734   .1923321    -0.11   0.912    -.3981373    .3557905
         L4. |   .3959794   .1987356     1.99   0.046     .0064648     .785494
             |
          ma |
         L1. |  -.2766778   2891.702    -0.00   1.000    -5667.908    5667.355
         L2. |  -.0254686   5840.076    -0.00   1.000    -11446.36    11446.31
         L3. |   .3217963   2948.522     0.00   1.000    -5778.674    5779.318
         L4. |   -1.01965          .        .       .            .           .
-------------+----------------------------------------------------------------
      /sigma |   .2271688   .0294384     7.72   0.000     .1694706    .2848669
------------------------------------------------------------------------------
Note: The test of the variance against zero is one sided, and the two-sided confidence interval
is truncated at zero.

.
. estat ic

-----------------------------------------------------------------------------
       Model |    Obs    ll(null)   ll(model)     df          AIC         BIC
-------------+---------------------------------------------------------------
           . |     70           .   -.8144143     12     25.62883    52.61077
-----------------------------------------------------------------------------
               Note:  N=Obs used in calculating BIC; see [R] BIC note

.
. estimates store M18

.
.
.
```

```
. arima lnHi L7DWP L7LST L7NDVI if tin(2012w19, 2013w36), arima(5,1,4)

(setting optimization to BHHH)
Iteration 0:   log likelihood = -8.6660413
Iteration 1:   log likelihood = -7.4933281
Iteration 2:   log likelihood = -6.6001479
Iteration 3:   log likelihood = -4.9265265
Iteration 4:   log likelihood = -3.0815571
(switching optimization to BFGS)
Iteration 5:   log likelihood = -3.0497983
Iteration 6:   log likelihood = -2.4608418
Iteration 7:   log likelihood = -2.1256286
Iteration 8:   log likelihood = -1.9522951
Iteration 9:   log likelihood = -1.9092147
Iteration 10:  log likelihood = -1.8852196
Iteration 11:  log likelihood = -1.8651819
Iteration 12:  log likelihood = -1.8484328
Iteration 13:  log likelihood =  -1.751299
Iteration 14:  log likelihood = -1.6314613
(switching optimization to BHHH)
Iteration 15:  log likelihood = -1.6254892
Iteration 16:  log likelihood = -1.6123204  (backed up)
Iteration 17:  log likelihood = -1.5871101
Iteration 18:  log likelihood = -1.5610015
Iteration 19:  log likelihood = -1.5532594  (backed up)
(switching optimization to BFGS)
Iteration 20:  log likelihood =  -1.546313  (backed up)
Iteration 21:  log likelihood = -1.5287667
Iteration 22:  log likelihood = -1.4694058
Iteration 23:  log likelihood = -1.3165206
Iteration 24:  log likelihood = -1.2329469
Iteration 25:  log likelihood = -1.1991476
Iteration 26:  log likelihood = -1.1990275
Iteration 27:  log likelihood = -1.1867903
Iteration 28:  log likelihood = -1.1867751
Iteration 29:  log likelihood = -1.1865219
(switching optimization to BHHH)
Iteration 30:  log likelihood = -1.1865143
Iteration 31:  log likelihood = -1.1865141  (backed up)
Iteration 32:  log likelihood = -1.1865141  (backed up)
Iteration 33:  log likelihood = -1.1865141  (backed up)
Iteration 34:  log likelihood =  -1.186514  (backed up)
(switching optimization to BFGS)
Iteration 35:  log likelihood =  -1.186514  (backed up)
Iteration 36:  log likelihood = -1.1865129  (backed up)
Iteration 37:  log likelihood = -1.1865098
Iteration 38:  log likelihood = -1.1865075
Iteration 39:  log likelihood = -1.1865071
Iteration 40:  log likelihood =  -1.186507

ARIMA regression

Sample:  2012w19 - 2013w36                      Number of obs      =        70
                                                Wald chi2(10)      =    302.86
Log likelihood = -1.186507                      Prob > chi2        =    0.0000

------------------------------------------------------------------------------
             |                 OPG
      D.lnHi |      Coef.   Std. Err.      z    P>|z|     [95% Conf. Interval]
-------------+----------------------------------------------------------------
lnHi         |
       L7DWP |
         D1. |   .0280905   .0123336     2.28   0.023     .0039171     .052264
             |
       L7LST |
         D1. |    .097955   .0231786     4.23   0.000     .0525259    .1433842
             |
      L7NDVI |
         D1. |   1.237662   .9204173     1.34   0.179    -.5663227    3.041647
             |
       _cons |  -.0122279    .034179    -0.36   0.721    -.0792175    .0547617
```

```
-------------+----------------------------------------------------------------
ARMA         |
          ar |
         L1. |  -1.494209    .539063    -2.77   0.006    -2.550754   -.4376654
         L2. |  -1.232403   .7607255    -1.62   0.105    -2.723398    .2585914
         L3. |  -.8083907   .7093903    -1.14   0.254     -2.19877    .5819888
         L4. |    .050606   .5727281     0.09   0.930     -1.07192    1.173132
         L5. |   .2338987   .1857721     1.26   0.208     -.130208    .5980053
             |
          ma |
         L1. |   1.153361          .        .       .            .           .
         L2. |   1.152874   .4760735     2.42   0.015     .2197872    2.085961
         L3. |   1.155986          .        .       .            .           .
         L4. |   .1564729   .5045041     0.31   0.756    -.8323369    1.145283
-------------+----------------------------------------------------------------
      /sigma |   .2330919   .0744154     3.13   0.001     .0872404    .3789435
------------------------------------------------------------------------------
Note: The test of the variance against zero is one sided, and the two-sided confidence interval
is truncated at zero.

.
. estat ic

-----------------------------------------------------------------------------
       Model |    Obs    ll(null)   ll(model)     df          AIC         BIC
-------------+---------------------------------------------------------------
           . |     70           .   -1.186507     12     26.37301    53.35496
-----------------------------------------------------------------------------
               Note:  N=Obs used in calculating BIC; see [R] BIC note

.
. estimates store M19

.
.
.
. arima lnHi L7DWP L7LST L7NDVI if tin(2012w19, 2013w36), arima(4,1,5)

(setting optimization to BHHH)
Iteration 0:   log likelihood = -8.3758502
Iteration 1:   log likelihood = -7.2040162
Iteration 2:   log likelihood = -6.9245898
Iteration 3:   log likelihood = -5.4282582
Iteration 4:   log likelihood = -5.4101131
(switching optimization to BFGS)
Iteration 5:   log likelihood = -4.4119618
Iteration 6:   log likelihood = -3.9043452  (backed up)
Iteration 7:   log likelihood = -3.7229266  (backed up)
Iteration 8:   log likelihood = -3.4317284
Iteration 9:   log likelihood = -3.2365334
Iteration 10:  log likelihood =  -3.014677
Iteration 11:  log likelihood = -2.1755105
Iteration 12:  log likelihood = -1.9069537
Iteration 13:  log likelihood = -1.7637311
Iteration 14:  log likelihood = -1.5559286
(switching optimization to BHHH)
Iteration 15:  log likelihood = -1.4732786
Iteration 16:  log likelihood = -1.2539688
Iteration 17:  log likelihood = -1.2513287
Iteration 18:  log likelihood = -1.1434955
Iteration 19:  log likelihood = -.91513965
(switching optimization to BFGS)
Iteration 20:  log likelihood = -.55701077
Iteration 21:  log likelihood = -.13032222
Iteration 22:  log likelihood = -.06174204
Iteration 23:  log likelihood =  .03395064
Iteration 24:  log likelihood =  .04272021
Iteration 25:  log likelihood =  .19725636
Iteration 26:  log likelihood =  .27626549
Iteration 27:  log likelihood =  .35741815
Iteration 28:  log likelihood =  .47111688
```

```
Iteration 29:  log likelihood =  .50297974
(switching optimization to BHHH)
Iteration 30:  log likelihood =  .66206583
Iteration 31:  log likelihood =  .66275296  (backed up)
Iteration 32:  log likelihood =  .70532878
Iteration 33:  log likelihood =  .99362873
Iteration 34:  log likelihood =  .99743614  (backed up)
(switching optimization to BFGS)
Iteration 35:  log likelihood =  1.1032547
Iteration 36:  log likelihood =  1.2091644
Iteration 37:  log likelihood =  1.3606033
Iteration 38:  log likelihood =  1.4093994
Iteration 39:  log likelihood =  1.4178106  (backed up)
Iteration 40:  log likelihood =  1.5967605
Iteration 41:  log likelihood =  1.6475316
Iteration 42:  log likelihood =  1.7164017
Iteration 43:  log likelihood =  1.7409038
Iteration 44:  log likelihood =  1.7464563
(switching optimization to BHHH)
Iteration 45:  log likelihood =  1.7492166
Iteration 46:  log likelihood =  1.7492174  (backed up)
Iteration 47:  log likelihood =  1.7492176  (not concave)
Iteration 48:  log likelihood =  1.7621849
Iteration 49:  log likelihood =  1.7623786  (backed up)
(switching optimization to BFGS)
Iteration 50:  log likelihood =  1.7647331  (backed up)
Iteration 51:  log likelihood =  1.7704945
Iteration 52:  log likelihood =  1.7741873
Iteration 53:  log likelihood =   1.775171
Iteration 54:  log likelihood =  1.7756693
Iteration 55:  log likelihood =  1.7777921
Iteration 56:  log likelihood =  1.7810642
Iteration 57:  log likelihood =  1.7812222
Iteration 58:  log likelihood =   1.781691
Iteration 59:  log likelihood =  1.7817063
(switching optimization to BHHH)
Iteration 60:  log likelihood =  1.7818446
Iteration 61:  log likelihood =  1.7818449  (backed up)
Iteration 62:  log likelihood =   1.781845  (not concave)
Iteration 63:  log likelihood =  1.7818503
Iteration 64:  log likelihood =  1.7818504  (not concave)
(switching optimization to BFGS)
Iteration 65:  log likelihood =  1.7818511
Iteration 66:  log likelihood =  1.7818511  (backed up)
Iteration 67:  log likelihood =  1.7818511  (backed up)
Iteration 68:  log likelihood =  1.7818519  (backed up)
Iteration 69:  log likelihood =  1.7818521  (backed up)
Iteration 70:  log likelihood =  1.7818522  (backed up)
Iteration 71:  log likelihood =  1.7818525  (backed up)
Iteration 72:  log likelihood =  1.7818526  (backed up)
Iteration 73:  log likelihood =  1.7818526  (backed up)
Iteration 74:  log likelihood =  1.7818531
(switching optimization to BHHH)
Iteration 75:  log likelihood =  1.7818533  (not concave)
Iteration 76:  log likelihood =  1.7818537
Iteration 77:  log likelihood =  1.7818537  (not concave)
Iteration 78:  log likelihood =  1.7818538  (not concave)
Iteration 79:  log likelihood =  1.7818539  (not concave)
(switching optimization to BFGS)
Iteration 80:  log likelihood =  1.7818539
Iteration 81:  log likelihood =  1.7818542  (backed up)
Iteration 82:  log likelihood =  1.7818544  (backed up)
Iteration 83:  log likelihood =  1.7818545  (backed up)
Iteration 84:  log likelihood =  1.7818545  (backed up)
Iteration 85:  log likelihood =  1.7818545  (backed up)
Iteration 86:  log likelihood =  1.7818545  (backed up)
Iteration 87:  log likelihood =  1.7818545  (backed up)
Iteration 88:  log likelihood =  1.7818546  (backed up)
Iteration 89:  log likelihood =  1.7818546  (backed up)
(switching optimization to BHHH)
Iteration 90:  log likelihood =  1.7818546  (not concave)
```

```
Iteration 91:  log likelihood =  1.7818546  (not concave)
Iteration 92:  log likelihood =  1.7818546  (not concave)
Iteration 93:  log likelihood =  1.7818546  (not concave)
Iteration 94:  log likelihood =  1.7818546  (not concave)
(switching optimization to BFGS)
Iteration 95:  log likelihood =  1.7818546
Iteration 96:  log likelihood =  1.7818546  (backed up)
Iteration 97:  log likelihood =  1.7818546  (backed up)
Iteration 98:  log likelihood =  1.7818547  (backed up)
Iteration 99:  log likelihood =  1.7818547  (backed up)
Iteration 100: log likelihood =  1.7818547  (backed up)
Iteration 101: log likelihood =  1.7818547  (backed up)
Iteration 102: log likelihood =  1.7818547  (backed up)
Iteration 103: log likelihood =  1.7818547  (backed up)
Iteration 104: log likelihood =  1.7818547  (backed up)
(switching optimization to BHHH)
Iteration 105: log likelihood =  1.7818547  (not concave)
Iteration 106: log likelihood =  1.7818547  (not concave)
Iteration 107: log likelihood =  1.7818547  (not concave)
Iteration 108: log likelihood =  1.7818547  (not concave)
Iteration 109: log likelihood =  1.7818547  (not concave)
(switching optimization to BFGS)
Iteration 110: log likelihood =  1.7818547

ARIMA regression

Sample:  2012w19 - 2013w36                      Number of obs      =        70
                                                Wald chi2(10)      =  56195.43
Log likelihood =  1.781855                      Prob > chi2        =    0.0000

------------------------------------------------------------------------------
             |                 OPG
      D.lnHi |      Coef.   Std. Err.      z    P>|z|     [95% Conf. Interval]
-------------+----------------------------------------------------------------
lnHi         |
       L7DWP |
         D1. |   .0323207   .0106787     3.03   0.002     .0113909    .0532506
             |
       L7LST |
         D1. |   .0928433   .0281087     3.30   0.001     .0377513    .1479352
             |
      L7NDVI |
         D1. |   1.094203    .739757     1.48   0.139    -.3556938      2.5441
             |
       _cons |   -.011172   .0423245    -0.26   0.792    -.0941265    .0717825
-------------+----------------------------------------------------------------
ARMA         |
          ar |
         L1. |  -1.539203   .2180173    -7.06   0.000    -1.966509   -1.111897
         L2. |  -1.708601   .3009988    -5.68   0.000    -2.298548   -1.118655
         L3. |  -1.282053   .2871917    -4.46   0.000    -1.844939    -.719168
         L4. |  -.2866432   .2204006    -1.30   0.193    -.7186204     .145334
             |
          ma |
         L1. |   1.228624   .2058173     5.97   0.000      .825229    1.632018
         L2. |   2.494192          .        .       .            .           .
         L3. |   2.631736          .        .       .            .           .
         L4. |   1.704252   .1995554     8.54   0.000     1.313131    2.095374
         L5. |   1.338078   .4764923     2.81   0.005     .4041704    2.271986
-------------+----------------------------------------------------------------
      /sigma |   .1641459   .0281133     5.84   0.000     .1090449    .2192469
------------------------------------------------------------------------------
Note: The test of the variance against zero is one sided, and the two-sided confidence interval
is truncated at zero.

.
. estat ic

-----------------------------------------------------------------------------
       Model |    Obs    ll(null)   ll(model)     df          AIC         BIC
-------------+---------------------------------------------------------------
```

```
           . |     70          .    1.781855     12     20.43629    47.41823
-----------------------------------------------------------------------------
               Note:  N=Obs used in calculating BIC; see [R] BIC note

.
. estimates store M20

.
.
.
. arima lnHi L7DWP L7LST L7NDVI if tin(2012w19, 2013w36), arima(5,1,5)

(setting optimization to BHHH)
Iteration 0:   log likelihood = -8.4349939
Iteration 1:   log likelihood = -7.2929985
Iteration 2:   log likelihood = -6.5863412
Iteration 3:   log likelihood =  -5.198018
Iteration 4:   log likelihood = -4.0494248
(switching optimization to BFGS)
Iteration 5:   log likelihood = -3.9987961
Iteration 6:   log likelihood = -3.7656007
Iteration 7:   log likelihood = -3.6407504
Iteration 8:   log likelihood = -2.7862247
Iteration 9:   log likelihood = -2.7182202
Iteration 10:  log likelihood = -2.3577141
Iteration 11:  log likelihood = -1.3342342
Iteration 12:  log likelihood = -1.1211243
Iteration 13:  log likelihood = -.47931093
Iteration 14:  log likelihood = -.00159409
(switching optimization to BHHH)
Iteration 15:  log likelihood =  .64498609
Iteration 16:  log likelihood =  .66912387  (backed up)
Iteration 17:  log likelihood =  .74859167  (backed up)
Iteration 18:  log likelihood =  .82555073  (backed up)
Iteration 19:  log likelihood =  .83916954  (backed up)
(switching optimization to BFGS)
Iteration 20:  log likelihood =  .88300907
Iteration 21:  log likelihood =  1.5047942
Iteration 22:  log likelihood =  1.9491435
Iteration 23:  log likelihood =  2.0303644
Iteration 24:  log likelihood =  2.1974149
Iteration 25:  log likelihood =  2.3363449
Iteration 26:  log likelihood =  2.5476129
Iteration 27:  log likelihood =  2.6654187
Iteration 28:  log likelihood =  2.7043335
Iteration 29:  log likelihood =  2.7059103
(switching optimization to BHHH)
Iteration 30:  log likelihood =  2.7155059
Iteration 31:  log likelihood =  2.7160107  (backed up)
Iteration 32:  log likelihood =   2.716388  (backed up)
Iteration 33:  log likelihood =  2.7170746  (backed up)
Iteration 34:  log likelihood =  2.7174297  (backed up)
(switching optimization to BFGS)
Iteration 35:  log likelihood =  2.7175773  (backed up)
Iteration 36:  log likelihood =   2.725268
Iteration 37:  log likelihood =  2.7316458
Iteration 38:  log likelihood =   2.740707
Iteration 39:  log likelihood =  2.7421546
Iteration 40:  log likelihood =  2.7440569
Iteration 41:  log likelihood =  2.7447739
Iteration 42:  log likelihood =  2.7456595
Iteration 43:  log likelihood =  2.7462631
Iteration 44:  log likelihood =  2.7462968
(switching optimization to BHHH)
Iteration 45:  log likelihood =  2.7463591
Iteration 46:  log likelihood =  2.7463591  (not concave)
Iteration 47:  log likelihood =  2.7463596  (not concave)
Iteration 48:  log likelihood =  2.7463598  (not concave)
Iteration 49:  log likelihood =  2.7463599
(switching optimization to BFGS)
Iteration 50:  log likelihood =  2.7463599
```

```
ARIMA regression

Sample:  2012w19 - 2013w36                        Number of obs      =        70
                                                  Wald chi2(12)      =  10386.45
Log likelihood =   2.74636                        Prob > chi2        =    0.0000

------------------------------------------------------------------------------
             |                 OPG
      D.lnHi |      Coef.   Std. Err.      z    P>|z|     [95% Conf. Interval]
-------------+----------------------------------------------------------------
lnHi         |
       L7DWP |
         D1. |   .0344682   .0133391     2.58   0.010     .0083241    .0606124
             |
       L7LST |
         D1. |   .0892618   .0277029     3.22   0.001     .0349652    .1435585
             |
      L7NDVI |
         D1. |   1.433432   .6205987     2.31   0.021     .2170813    2.649783
             |
       _cons |  -.0101395   .0345735    -0.29   0.769    -.0779022    .0576233
-------------+----------------------------------------------------------------
ARMA         |
          ar |
         L1. |  -1.702687   .2898665    -5.87   0.000    -2.270815   -1.134559
         L2. |  -2.179833   .5184777    -4.20   0.000     -3.19603   -1.163635
         L3. |  -1.876594   .6429881    -2.92   0.004    -3.136828   -.6163604
         L4. |  -.8024185   .5060958    -1.59   0.113    -1.794348    .1895109
         L5. |  -.2849221   .2491764    -1.14   0.253    -.7732988    .2034546
             |
          ma |
         L1. |   1.418338   .1997268     7.10   0.000     1.026881    1.809795
         L2. |   2.287242          .        .       .            .           .
         L3. |   2.264774   .2488493     9.10   0.000     1.777039     2.75251
         L4. |   1.258892   .0705503    17.84   0.000     1.120616    1.397168
         L5. |   .9108266   .2415461     3.77   0.000     .4374049    1.384248
-------------+----------------------------------------------------------------
      /sigma |   .2141926   .0504139     4.25   0.000     .1153832     .313002
------------------------------------------------------------------------------
Note: The test of the variance against zero is one sided, and the two-sided confidence interval
is truncated at zero.

.
. estat ic

-----------------------------------------------------------------------------
       Model |    Obs    ll(null)   ll(model)     df          AIC         BIC
-------------+---------------------------------------------------------------
           . |     70           .     2.74636     14     22.50728    53.98621
-----------------------------------------------------------------------------
               Note:  N=Obs used in calculating BIC; see [R] BIC note

.
. estimates store M201

.
.
.
. arima lnHi L7DWP L7LST L7NDVI if tin(2012w19, 2013w36), arima(6,1,5)

(setting optimization to BHHH)
Iteration 0:   log likelihood = -8.4983424
Iteration 1:   log likelihood = -7.7806043
Iteration 2:   log likelihood = -2.5295176
Iteration 3:   log likelihood = -.82861412
Iteration 4:   log likelihood =  .21502057
(switching optimization to BFGS)
Iteration 5:   log likelihood =  .49062186
Iteration 6:   log likelihood =  1.0122859
Iteration 7:   log likelihood =  1.2426824  (backed up)
```

```
Iteration 8:   log likelihood =  2.1311737
Iteration 9:   log likelihood =   2.911629
Iteration 10:  log likelihood =  3.2472176
Iteration 11:  log likelihood =  3.3452208
Iteration 12:  log likelihood =  3.7472824
Iteration 13:  log likelihood =  3.8876548
Iteration 14:  log likelihood =  3.9140271
(switching optimization to BHHH)
Iteration 15:  log likelihood =  4.3092151
Iteration 16:  log likelihood =  4.3099856  (backed up)
Iteration 17:  log likelihood =  4.3104457  (backed up)
Iteration 18:  log likelihood =  4.3108437  (backed up)
Iteration 19:  log likelihood =  4.3109242  (backed up)
(switching optimization to BFGS)
Iteration 20:  log likelihood =  4.3111349  (backed up)
Iteration 21:  log likelihood =  4.3198467  (backed up)
Iteration 22:  log likelihood =  4.5266731
Iteration 23:  log likelihood =  4.5973076
Iteration 24:  log likelihood =  5.0379514
Iteration 25:  log likelihood =  5.1199352
Iteration 26:  log likelihood =  5.6985621
Iteration 27:  log likelihood =  5.8394127
Iteration 28:  log likelihood =  6.1943807
Iteration 29:  log likelihood =  6.3038509
(switching optimization to BHHH)
Iteration 30:  log likelihood =  6.4285891
Iteration 31:  log likelihood =   6.445726  (backed up)
Iteration 32:  log likelihood =  6.4518256  (backed up)
Iteration 33:  log likelihood =  6.4569345  (backed up)
Iteration 34:  log likelihood =  6.4579724  (backed up)
(switching optimization to BFGS)
Iteration 35:  log likelihood =  6.4583558  (backed up)
Iteration 36:  log likelihood =  6.4589535  (backed up)
Iteration 37:  log likelihood =  6.4625017
Iteration 38:  log likelihood =  6.5610228
Iteration 39:  log likelihood =  6.5766496
Iteration 40:  log likelihood =  6.6788158
Iteration 41:  log likelihood =   6.682801
Iteration 42:  log likelihood =   6.698556
Iteration 43:  log likelihood =  6.7091954
Iteration 44:  log likelihood =  6.7121414
(switching optimization to BHHH)
Iteration 45:  log likelihood =   6.712269
Iteration 46:  log likelihood =  6.7122691  (backed up)
Iteration 47:  log likelihood =  6.7122692  (not concave)
Iteration 48:  log likelihood =  6.7122738  (not concave)
Iteration 49:  log likelihood =  6.7122778  (not concave)
(switching optimization to BFGS)
Iteration 50:  log likelihood =  6.7122798
Iteration 51:  log likelihood =  6.7122826  (backed up)
Iteration 52:  log likelihood =  6.7122843  (backed up)
Iteration 53:  log likelihood =  6.7122845  (backed up)
Iteration 54:  log likelihood =  6.7122849  (backed up)
Iteration 55:  log likelihood =  6.7122852  (backed up)
Iteration 56:  log likelihood =  6.7122853  (backed up)
Iteration 57:  log likelihood =  6.7122853  (backed up)
Iteration 58:  log likelihood =  6.7122853  (backed up)
Iteration 59:  log likelihood =  6.7122853  (backed up)
(switching optimization to BHHH)
Iteration 60:  log likelihood =  6.7122854  (not concave)
Iteration 61:  log likelihood =  6.7122861  (not concave)
Iteration 62:  log likelihood =  6.7122875  (not concave)
Iteration 63:  log likelihood =  6.7122883  (not concave)
Iteration 64:  log likelihood =  6.7122885  (not concave)
(switching optimization to BFGS)
Iteration 65:  log likelihood =  6.7122886
Iteration 66:  log likelihood =  6.7122886

ARIMA regression

Sample:  2012w19 - 2013w36                      Number of obs      =        70
```

```
                                                Wald chi2(12)      =   3412.06
Log likelihood =  6.712289                      Prob > chi2        =    0.0000

------------------------------------------------------------------------------
             |                 OPG
      D.lnHi |      Coef.   Std. Err.      z    P>|z|     [95% Conf. Interval]
-------------+----------------------------------------------------------------
lnHi         |
       L7DWP |
         D1. |   .0455337   .0114499     3.98   0.000     .0230922    .0679751
             |
       L7LST |
         D1. |   .1298926    .019186     6.77   0.000     .0922888    .1674965
             |
      L7NDVI |
         D1. |   1.856481   .3100433     5.99   0.000     1.248807    2.464155
             |
       _cons |  -.0088469   .0014911    -5.93   0.000    -.0117694   -.0059243
-------------+----------------------------------------------------------------
ARMA         |
          ar |
         L1. |  -.0343182   .1879693    -0.18   0.855    -.4027314    .3340949
         L2. |    .398163   .1595684     2.50   0.013     .0854147    .7109112
         L3. |   .3644989   .2214269     1.65   0.100    -.0694899    .7984876
         L4. |   .5785544   .1322369     4.38   0.000     .3193749    .8377339
         L5. |  -.5280918   .1337843    -3.95   0.000    -.7903041   -.2658794
         L6. |  -.4942581   .1644867    -3.00   0.003    -.8166462   -.1718701
             |
          ma |
         L1. |  -.7265425   .8227189    -0.88   0.377    -2.339042    .8859569
         L2. |  -.2957482          .        .       .            .           .
         L3. |  -.1713601   .3885656    -0.44   0.659    -.9329347    .5902145
         L4. |  -.8307625   .6160885    -1.35   0.178    -2.038274    .3767487
         L5. |   1.037705          .        .       .            .           .
-------------+----------------------------------------------------------------
      /sigma |   .1905656   .0295717     6.44   0.000     .1326062     .248525
------------------------------------------------------------------------------
Note: The test of the variance against zero is one sided, and the two-sided confidence interval
is truncated at zero.

.
. estat ic

-----------------------------------------------------------------------------
       Model |    Obs    ll(null)   ll(model)     df          AIC         BIC
-------------+---------------------------------------------------------------
           . |     70           .    6.712289     14     14.57542    46.05436
-----------------------------------------------------------------------------
               Note:  N=Obs used in calculating BIC; see [R] BIC note

.
. estimates store M202

.
.
.
. arima lnHi L7DWP L7LST if tin(2012w19, 2013w36), arima(1,1,1)

(setting optimization to BHHH)
Iteration 0:   log likelihood = -11.513957
Iteration 1:   log likelihood = -11.005714
Iteration 2:   log likelihood = -10.901356
Iteration 3:   log likelihood = -10.893997
Iteration 4:   log likelihood = -10.890163
(switching optimization to BFGS)
Iteration 5:   log likelihood = -10.889136
Iteration 6:   log likelihood = -10.888497
Iteration 7:   log likelihood = -10.888494
Iteration 8:   log likelihood = -10.888494

ARIMA regression
```

```
Sample:  2012w19 - 2013w36                        Number of obs      =        70
                                                  Wald chi2(4)       =     32.56
Log likelihood = -10.88849                        Prob > chi2        =    0.0000

------------------------------------------------------------------------------
             |                 OPG
      D.lnHi |      Coef.   Std. Err.      z    P>|z|     [95% Conf. Interval]
-------------+----------------------------------------------------------------
lnHi         |
       L7DWP |
         D1. |   .0352464   .0167862     2.10   0.036      .002346    .0681468
             |
       L7LST |
         D1. |    .103655   .0251477     4.12   0.000     .0543665    .1529435
             |
       _cons |  -.0208092   .0257976    -0.81   0.420    -.0713715    .0297531
-------------+----------------------------------------------------------------
ARMA         |
          ar |
         L1. |  -.4149715    .333382    -1.24   0.213    -1.068388    .2384452
             |
          ma |
         L1. |   .0601303   .3979042     0.15   0.880    -.7197476    .8400082
-------------+----------------------------------------------------------------
      /sigma |   .2824101   .0290265     9.73   0.000     .2255192    .3393011
------------------------------------------------------------------------------
Note: The test of the variance against zero is one sided, and the two-sided confidence interval
is truncated at zero.

.
. estat ic

-----------------------------------------------------------------------------
       Model |    Obs    ll(null)   ll(model)     df          AIC         BIC
-------------+---------------------------------------------------------------
           . |     70           .   -10.88849      6     33.77699    47.26796
-----------------------------------------------------------------------------
               Note:  N=Obs used in calculating BIC; see [R] BIC note

.
. estimates store M21

.
.
.
. arima lnHi L7DWP L7LST if tin(2012w19, 2013w36), arima(2,1,1)

(setting optimization to BHHH)
Iteration 0:   log likelihood = -11.890506
Iteration 1:   log likelihood = -11.352826
Iteration 2:   log likelihood = -11.184893
Iteration 3:   log likelihood = -11.060925
Iteration 4:   log likelihood =  -10.93418
(switching optimization to BFGS)
Iteration 5:   log likelihood = -10.909406
Iteration 6:   log likelihood = -10.861383
Iteration 7:   log likelihood = -10.816139
Iteration 8:   log likelihood = -10.734573
Iteration 9:   log likelihood = -10.734573  (backed up)
Iteration 10:  log likelihood = -10.734573  (backed up)
Iteration 11:  log likelihood = -10.734573  (backed up)
BFGS stepping has contracted, resetting BFGS Hessian (0)
Iteration 12:  log likelihood = -10.734573  (backed up)
Iteration 13:  log likelihood = -10.576069  (backed up)
Iteration 14:  log likelihood = -10.512666  (backed up)
(switching optimization to BHHH)
Iteration 15:  log likelihood = -10.039145  (backed up)
Iteration 16:  log likelihood = -9.9471668
Iteration 17:  log likelihood = -9.8420982
Iteration 18:  log likelihood =  -9.840031  (backed up)
```

```
Iteration 19:  log likelihood = -9.8394741  (backed up)
(switching optimization to BFGS)
Iteration 20:  log likelihood = -9.8393958  (backed up)
Iteration 21:  log likelihood = -9.7869523
Iteration 22:  log likelihood = -9.7829896
Iteration 23:  log likelihood = -9.7791964
Iteration 24:  log likelihood = -9.7790818
Iteration 25:  log likelihood = -9.7790392
Iteration 26:  log likelihood = -9.7790383

ARIMA regression

Sample:  2012w19 - 2013w36                      Number of obs      =        70
                                                Wald chi2(5)       =    456.28
Log likelihood = -9.779038                      Prob > chi2        =    0.0000

------------------------------------------------------------------------------
             |                 OPG
      D.lnHi |      Coef.   Std. Err.      z    P>|z|     [95% Conf. Interval]
-------------+----------------------------------------------------------------
lnHi         |
       L7DWP |
         D1. |    .032302   .0155313     2.08   0.038     .0018612    .0627428
             |
       L7LST |
         D1. |   .1079328   .0254211     4.25   0.000     .0581085    .1577572
             |
       _cons |  -.0209254   .0259211    -0.81   0.420      -.07173    .0298791
-------------+----------------------------------------------------------------
ARMA         |
          ar |
         L1. |  -1.325108   .1291718   -10.26   0.000     -1.57828   -1.071936
         L2. |  -.4029192   .1392834    -2.89   0.004    -.6759097   -.1299288
             |
          ma |
         L1. |   .9999958   604.2722     0.00   0.999    -1183.352    1185.352
-------------+----------------------------------------------------------------
      /sigma |   .2744847   82.91785     0.00   0.499            0    162.7905
------------------------------------------------------------------------------
Note: The test of the variance against zero is one sided, and the two-sided confidence interval
is truncated at zero.

.
. estat ic

-----------------------------------------------------------------------------
       Model |    Obs    ll(null)   ll(model)     df          AIC         BIC
-------------+---------------------------------------------------------------
           . |     70           .   -9.779038      7     33.55808    49.29754
-----------------------------------------------------------------------------
               Note:  N=Obs used in calculating BIC; see [R] BIC note

.
. estimates store M22

.
.
.
. arima lnHi L7DWP L7LST if tin(2012w19, 2013w36), arima(1,1,2)

(setting optimization to BHHH)
Iteration 0:   log likelihood = -11.023342
Iteration 1:   log likelihood = -9.6723745
Iteration 2:   log likelihood = -8.9384683
Iteration 3:   log likelihood = -8.8937635
Iteration 4:   log likelihood = -8.7498709
(switching optimization to BFGS)
Iteration 5:   log likelihood =  -8.721359
Iteration 6:   log likelihood =   -8.66917
Iteration 7:   log likelihood = -8.6659805
Iteration 8:   log likelihood =  -8.664499
```

```
Iteration 9:   log likelihood = -8.6643219
Iteration 10:  log likelihood = -8.6642787
Iteration 11:  log likelihood = -8.6642777
Iteration 12:  log likelihood = -8.6642776

ARIMA regression

Sample:  2012w19 - 2013w36                      Number of obs      =        70
                                                Wald chi2(5)       =     38.84
Log likelihood = -8.664278                      Prob > chi2        =    0.0000

------------------------------------------------------------------------------
             |                 OPG
      D.lnHi |      Coef.   Std. Err.      z    P>|z|     [95% Conf. Interval]
-------------+----------------------------------------------------------------
lnHi         |
       L7DWP |
         D1. |    .035216    .013976     2.52   0.012     .0078236    .0626083
             |
       L7LST |
         D1. |   .1157995   .0275287     4.21   0.000     .0618443    .1697548
             |
       _cons |  -.0191386   .0375829    -0.51   0.611    -.0927997    .0545225
-------------+----------------------------------------------------------------
ARMA         |
          ar |
         L1. |   .1307163   .4053583     0.32   0.747    -.6637714    .9252039
             |
          ma |
         L1. |  -.5145005   .3633739    -1.42   0.157      -1.2267    .1976993
         L2. |   .4233441   .1378593     3.07   0.002     .1531448    .6935435
-------------+----------------------------------------------------------------
      /sigma |   .2730167   .0292358     9.34   0.000     .2157155    .3303179
------------------------------------------------------------------------------
Note: The test of the variance against zero is one sided, and the two-sided confidence interval
is truncated at zero.

.
. estat ic

-----------------------------------------------------------------------------
       Model |    Obs    ll(null)   ll(model)     df          AIC         BIC
-------------+---------------------------------------------------------------
           . |     70           .   -8.664278      7     31.32856    47.06802
-----------------------------------------------------------------------------
               Note:  N=Obs used in calculating BIC; see [R] BIC note

.
. estimates store M23

.
.
.
. arima lnHi L7DWP L7LST if tin(2012w19, 2013w36), arima(3,1,1)

(setting optimization to BHHH)
Iteration 0:   log likelihood = -11.133686
Iteration 1:   log likelihood = -9.7025731
Iteration 2:   log likelihood =  -9.446757
Iteration 3:   log likelihood = -9.4138499
Iteration 4:   log likelihood = -9.3811795
(switching optimization to BFGS)
Iteration 5:   log likelihood =  -9.361189
Iteration 6:   log likelihood =  -9.334346
Iteration 7:   log likelihood = -9.3320159
Iteration 8:   log likelihood = -9.3313732
Iteration 9:   log likelihood =  -9.331326
Iteration 10:  log likelihood = -9.3313083
Iteration 11:  log likelihood =  -9.331308

ARIMA regression
```

```
Sample:  2012w19 - 2013w36                        Number of obs      =        70
                                                  Wald chi2(6)       =     33.35
Log likelihood = -9.331308                        Prob > chi2        =    0.0000

------------------------------------------------------------------------------
             |                 OPG
      D.lnHi |      Coef.   Std. Err.      z    P>|z|     [95% Conf. Interval]
-------------+----------------------------------------------------------------
lnHi         |
       L7DWP |
         D1. |   .0355708   .0159435     2.23   0.026     .0043221    .0668195
             |
       L7LST |
         D1. |   .1127562   .0273669     4.12   0.000     .0591182    .1663943
             |
       _cons |  -.0202084   .0349757    -0.58   0.563    -.0887595    .0483428
-------------+----------------------------------------------------------------
ARMA         |
          ar |
         L1. |  -.2918563   .6880835    -0.42   0.671    -1.640475    1.056763
         L2. |   .1418157   .2683304     0.53   0.597    -.3841023    .6677337
         L3. |   .2090785   .1254461     1.67   0.096    -.0367913    .4549483
             |
          ma |
         L1. |  -.0671967   .6740259    -0.10   0.921    -1.388263     1.25387
-------------+----------------------------------------------------------------
      /sigma |   .2759132   .0296768     9.30   0.000     .2177479    .3340786
------------------------------------------------------------------------------
Note: The test of the variance against zero is one sided, and the two-sided confidence interval
is truncated at zero.

.
. estat ic

-----------------------------------------------------------------------------
       Model |    Obs    ll(null)   ll(model)     df          AIC         BIC
-------------+---------------------------------------------------------------
           . |     70           .   -9.331308      8     34.66262    52.65058
-----------------------------------------------------------------------------
               Note:  N=Obs used in calculating BIC; see [R] BIC note

.
. estimates store M24

.
.
.
. arima lnHi L7DWP L7LST if tin(2012w19, 2013w36), arima(1,1,3)

(setting optimization to BHHH)
Iteration 0:   log likelihood = -11.377772
Iteration 1:   log likelihood = -9.3550268
Iteration 2:   log likelihood = -8.4490478
Iteration 3:   log likelihood = -8.1654966
Iteration 4:   log likelihood = -8.0258455
(switching optimization to BFGS)
Iteration 5:   log likelihood = -7.9593984
Iteration 6:   log likelihood = -7.3715558
Iteration 7:   log likelihood = -7.2792805
Iteration 8:   log likelihood = -7.2583827
Iteration 9:   log likelihood = -7.2447188
Iteration 10:  log likelihood = -7.2442876
Iteration 11:  log likelihood = -7.2442269
Iteration 12:  log likelihood = -7.2442202
Iteration 13:  log likelihood = -7.2442199

ARIMA regression

Sample:  2012w19 - 2013w36                        Number of obs      =        70
                                                  Wald chi2(6)       =    135.79
```

```
Log likelihood =  -7.24422                        Prob > chi2       =    0.0000

------------------------------------------------------------------------------
             |                 OPG
      D.lnHi |      Coef.   Std. Err.      z    P>|z|     [95% Conf. Interval]
-------------+----------------------------------------------------------------
lnHi         |
       L7DWP |
         D1. |   .0329685    .013817     2.39   0.017     .0058876    .0600493
             |
       L7LST |
         D1. |   .1189216   .0272758     4.36   0.000      .065462    .1723813
             |
       _cons |  -.0187553   .0387049    -0.48   0.628    -.0946154    .0571049
-------------+----------------------------------------------------------------
ARMA         |
          ar |
         L1. |  -.8495896   .1299484    -6.54   0.000    -1.104284   -.5948953
             |
          ma |
         L1. |   .5385544   281.0097     0.00   0.998    -550.2303    551.3074
         L2. |   .0009602    129.711     0.00   1.000    -254.2279    254.2299
         L3. |   .4624248   129.9882     0.00   0.997    -254.3097    255.2345
-------------+----------------------------------------------------------------
      /sigma |   .2640037   37.11367     0.01   0.497            0    73.00545
------------------------------------------------------------------------------
Note: The test of the variance against zero is one sided, and the two-sided confidence interval
is truncated at zero.

.
. estat ic

-----------------------------------------------------------------------------
       Model |    Obs    ll(null)   ll(model)     df          AIC         BIC
-------------+---------------------------------------------------------------
           . |     70           .    -7.24422      8     30.48844     48.4764
-----------------------------------------------------------------------------
               Note:  N=Obs used in calculating BIC; see [R] BIC note

.
. estimates store M25

.
.
.
. arima lnHi L7DWP L7LST if tin(2012w19, 2013w36), arima(3,1,2)

(setting optimization to BHHH)
Iteration 0:   log likelihood = -10.874276
Iteration 1:   log likelihood = -9.2671279
Iteration 2:   log likelihood = -6.4539875
Iteration 3:   log likelihood = -5.5789403
Iteration 4:   log likelihood =  -4.130617
(switching optimization to BFGS)
Iteration 5:   log likelihood = -4.1259038
Iteration 6:   log likelihood = -3.8741071
Iteration 7:   log likelihood = -3.6244149
Iteration 8:   log likelihood =  -3.593672
Iteration 9:   log likelihood = -3.5914595
Iteration 10:  log likelihood = -3.5876556
Iteration 11:  log likelihood = -3.5874999
Iteration 12:  log likelihood = -3.5874855
Iteration 13:  log likelihood = -3.5874842
Iteration 14:  log likelihood = -3.5874842

ARIMA regression

Sample:  2012w19 - 2013w36                        Number of obs     =        70
                                                  Wald chi2(6)      =    166.70
Log likelihood = -3.587484                        Prob > chi2       =    0.0000
```

```
------------------------------------------------------------------------------
             |                 OPG
      D.lnHi |      Coef.   Std. Err.      z    P>|z|     [95% Conf. Interval]
-------------+----------------------------------------------------------------
lnHi         |
       L7DWP |
         D1. |   .0319624   .0085803     3.73   0.000     .0151452    .0487795
             |
       L7LST |
         D1. |   .0955552   .0214199     4.46   0.000     .0535729    .1375375
             |
       _cons |  -.0204758   .0332836    -0.62   0.538    -.0857104    .0447588
-------------+----------------------------------------------------------------
ARMA         |
          ar |
         L1. |  -.0752773   .1447457    -0.52   0.603    -.3589737     .208419
         L2. |  -.5590046    .132473    -4.22   0.000    -.8186469   -.2993623
         L3. |   -.039912   .1321117    -0.30   0.763    -.2988463    .2190222
             |
          ma |
         L1. |  -.3264749   .0830424    -3.93   0.000    -.4892351   -.1637147
         L2. |          1          .        .       .            .           .
-------------+----------------------------------------------------------------
      /sigma |   .2451178   .0258135     9.50   0.000     .1945243    .2957114
------------------------------------------------------------------------------
Note: The test of the variance against zero is one sided, and the two-sided confidence interval
is truncated at zero.

.
. estat ic

-----------------------------------------------------------------------------
       Model |    Obs    ll(null)   ll(model)     df          AIC         BIC
-------------+---------------------------------------------------------------
           . |     70           .   -3.587484      8     23.17497    41.16293
-----------------------------------------------------------------------------
               Note:  N=Obs used in calculating BIC; see [R] BIC note

.
. estimates store M26

.
.
.
. arima lnHi L7DWP L7LST if tin(2012w19, 2013w36), arima(2,1,3)

(setting optimization to BHHH)
Iteration 0:   log likelihood = -9.6982613  
Iteration 1:   log likelihood = -8.8527218  
Iteration 2:   log likelihood = -7.6749049  
Iteration 3:   log likelihood = -7.5666556  
Iteration 4:   log likelihood = -7.4944714  
(switching optimization to BFGS)
Iteration 5:   log likelihood = -7.0459002  
Iteration 6:   log likelihood =   -6.04304  
Iteration 7:   log likelihood = -5.1162593  
Iteration 8:   log likelihood = -4.4713257  
Iteration 9:   log likelihood = -4.1301045  
Iteration 10:  log likelihood = -4.0523666  
Iteration 11:  log likelihood =  -3.900442  
Iteration 12:  log likelihood = -3.6377185  
Iteration 13:  log likelihood = -3.6227877  
Iteration 14:  log likelihood = -3.6009898  
(switching optimization to BHHH)
Iteration 15:  log likelihood = -3.5978399  
Iteration 16:  log likelihood = -3.5978189  (backed up)
Iteration 17:  log likelihood =  -3.597807  (backed up)
Iteration 18:  log likelihood =  -3.597807  (backed up)
Iteration 19:  log likelihood = -3.5978069  (backed up)
(switching optimization to BFGS)
Iteration 20:  log likelihood = -3.5978069  (backed up)
```

```
Iteration 21:  log likelihood = -3.5973292
Iteration 22:  log likelihood = -3.5968487
Iteration 23:  log likelihood = -3.5967364
Iteration 24:  log likelihood = -3.5966789
Iteration 25:  log likelihood = -3.5966704
Iteration 26:  log likelihood = -3.5966661
Iteration 27:  log likelihood = -3.5966661

ARIMA regression

Sample:  2012w19 - 2013w36                        Number of obs      =        70
                                                  Wald chi2(6)       =    168.12
Log likelihood = -3.596666                        Prob > chi2        =    0.0000

------------------------------------------------------------------------------
             |                 OPG
      D.lnHi |      Coef.   Std. Err.      z    P>|z|     [95% Conf. Interval]
-------------+----------------------------------------------------------------
lnHi         |
       L7DWP |
         D1. |   .0319703   .0085403     3.74   0.000     .0152317     .048709
             |
       L7LST |
         D1. |   .0954343   .0213476     4.47   0.000     .0535939    .1372748
             |
       _cons |  -.0204479   .0334906    -0.61   0.541    -.0860882    .0451924
-------------+----------------------------------------------------------------
ARMA         |
          ar |
         L1. |  -.0118645   .2195761    -0.05   0.957    -.4422257    .4184967
         L2. |  -.5551411   .1308421    -4.24   0.000     -.811587   -.2986952
             |
          ma |
         L1. |  -.3863592   .2464936    -1.57   0.117    -.8694777    .0967593
         L2. |    1.01953          .        .       .            .           .
         L3. |  -.0598069   .2399576    -0.25   0.803    -.5301152    .4105013
-------------+----------------------------------------------------------------
      /sigma |   .2451048   .0265187     9.24   0.000      .193129    .2970805
------------------------------------------------------------------------------
Note: The test of the variance against zero is one sided, and the two-sided confidence interval
is truncated at zero.

.
. estat ic

-----------------------------------------------------------------------------
       Model |    Obs    ll(null)   ll(model)     df          AIC         BIC
-------------+---------------------------------------------------------------
           . |     70           .   -3.596666      8     23.19333    41.18129
-----------------------------------------------------------------------------
               Note:  N=Obs used in calculating BIC; see [R] BIC note

.
. estimates store M27

.
.
.
. arima lnHi L7DWP L7LST if tin(2012w19, 2013w36), arima(3,1,3)

(setting optimization to BHHH)
Iteration 0:   log likelihood = -9.5910039
Iteration 1:   log likelihood = -6.5356204
Iteration 2:   log likelihood = -6.1924862
Iteration 3:   log likelihood = -6.0367742
Iteration 4:   log likelihood = -4.4508987
(switching optimization to BFGS)
Iteration 5:   log likelihood = -4.2313348
Iteration 6:   log likelihood = -4.1323593  (backed up)
Iteration 7:   log likelihood = -3.5765298
Iteration 8:   log likelihood = -3.5108529
```

```
Iteration 9:   log likelihood = -3.4569551
Iteration 10:  log likelihood = -3.4133925
Iteration 11:  log likelihood =  -3.380646
Iteration 12:  log likelihood = -3.3463118
Iteration 13:  log likelihood = -3.2053805
Iteration 14:  log likelihood = -3.1683602
(switching optimization to BHHH)
Iteration 15:  log likelihood =  -3.139898
Iteration 16:  log likelihood = -3.1390726  (backed up)
Iteration 17:  log likelihood = -3.1387659  (backed up)
Iteration 18:  log likelihood = -3.1386872  (backed up)
Iteration 19:  log likelihood = -3.1381542  (backed up)
(switching optimization to BFGS)
Iteration 20:  log likelihood = -3.1381207  (backed up)
Iteration 21:  log likelihood = -3.1380868  (backed up)
Iteration 22:  log likelihood = -3.1372519
Iteration 23:  log likelihood = -3.1357575
Iteration 24:  log likelihood = -3.1356917
Iteration 25:  log likelihood = -3.1356505
Iteration 26:  log likelihood = -3.1356478
Iteration 27:  log likelihood = -3.1356474

ARIMA regression

Sample:  2012w19 - 2013w36                        Number of obs      =        70
                                                  Wald chi2(6)       =    132.51
Log likelihood = -3.135647                        Prob > chi2        =    0.0000

------------------------------------------------------------------------------
             |                 OPG
      D.lnHi |      Coef.   Std. Err.      z    P>|z|     [95% Conf. Interval]
-------------+----------------------------------------------------------------
lnHi         |
       L7DWP |
         D1. |   .0302273   .0078069     3.87   0.000      .014926    .0455286
             |
       L7LST |
         D1. |   .0977306   .0210483     4.64   0.000     .0564766    .1389845
             |
       _cons |  -.0202096   .0350825    -0.58   0.565      -.08897    .0485508
-------------+----------------------------------------------------------------
ARMA         |
          ar |
         L1. |  -.9913755   .1365483    -7.26   0.000    -1.259005   -.7237458
         L2. |  -.5938832   .2044511    -2.90   0.004    -.9946001   -.1931664
         L3. |  -.4937172   .1492344    -3.31   0.001    -.7862113   -.2012231
             |
          ma |
         L1. |   .6708793   .1667768     4.02   0.000     .3440027    .9977559
         L2. |   .6708795          .        .       .            .           .
         L3. |   .9999995          .        .       .            .           .
-------------+----------------------------------------------------------------
      /sigma |   .2409237   .0248145     9.71   0.000     .1922883    .2895592
------------------------------------------------------------------------------
Note: The test of the variance against zero is one sided, and the two-sided confidence interval
is truncated at zero.

.
. estat ic

-----------------------------------------------------------------------------
       Model |    Obs    ll(null)   ll(model)     df          AIC         BIC
-------------+---------------------------------------------------------------
           . |     70           .   -3.135647      8     22.27129    40.25926
-----------------------------------------------------------------------------
               Note:  N=Obs used in calculating BIC; see [R] BIC note

.
. estimates store M28

.
```

```
.
.
. arima lnHi L7DWP L7LST if tin(2012w19, 2013w36), arima(4,1,3)

(setting optimization to BHHH)
Iteration 0:   log likelihood = -10.063851
Iteration 1:   log likelihood = -7.5392755
Iteration 2:   log likelihood = -6.5304014
Iteration 3:   log likelihood =  -5.293059
Iteration 4:   log likelihood = -4.9550352
(switching optimization to BFGS)
Iteration 5:   log likelihood =    -4.5557
Iteration 6:   log likelihood = -4.1447811  (backed up)
Iteration 7:   log likelihood = -3.7014782
Iteration 8:   log likelihood = -3.4484612
Iteration 9:   log likelihood = -3.3445364
Iteration 10:  log likelihood = -3.2669762
Iteration 11:  log likelihood = -2.9438286
Iteration 12:  log likelihood = -2.8883896
Iteration 13:  log likelihood = -2.8809083
Iteration 14:  log likelihood = -2.8790081
(switching optimization to BHHH)
Iteration 15:  log likelihood = -2.8787865
Iteration 16:  log likelihood = -2.8787855  (backed up)
Iteration 17:  log likelihood = -2.8787854  (backed up)
Iteration 18:  log likelihood = -2.8787853  (backed up)
Iteration 19:  log likelihood = -2.8787853  (backed up)
(switching optimization to BFGS)
Iteration 20:  log likelihood = -2.8787853  (backed up)
Iteration 21:  log likelihood = -2.8787839  (backed up)
Iteration 22:  log likelihood = -2.8787754
Iteration 23:  log likelihood = -2.8787738
Iteration 24:  log likelihood = -2.8787734

ARIMA regression

Sample:  2012w19 - 2013w36                      Number of obs      =        70
                                                Wald chi2(8)       =    190.70
Log likelihood = -2.878773                      Prob > chi2        =    0.0000

------------------------------------------------------------------------------
             |                 OPG
      D.lnHi |      Coef.   Std. Err.      z    P>|z|     [95% Conf. Interval]
-------------+----------------------------------------------------------------
lnHi         |
       L7DWP |
         D1. |   .0299824   .0084759     3.54   0.000     .0133699    .0465949
             |
       L7LST |
         D1. |   .1000267    .022119     4.52   0.000     .0566743     .143379
             |
       _cons |  -.0205276   .0329182    -0.62   0.533    -.0850461    .0439908
-------------+----------------------------------------------------------------
ARMA         |
          ar |
         L1. |  -1.021841   .1617596    -6.32   0.000    -1.338884   -.7047977
         L2. |  -.6406856    .220212    -2.91   0.004    -1.072293    -.209078
         L3. |  -.5767076   .1904488    -3.03   0.002    -.9499804   -.2034348
         L4. |  -.0899142   .1439119    -0.62   0.532    -.3719763    .1921479
             |
          ma |
         L1. |     .66627     1475.2     0.00   1.000    -2890.673    2892.005
         L2. |   .6662668   1475.198     0.00   1.000    -2890.669    2892.002
         L3. |   1.000001          .        .       .            .           .
-------------+----------------------------------------------------------------
      /sigma |   .2402228   .0295312     8.13   0.000     .1823427    .2981029
------------------------------------------------------------------------------
Note: The test of the variance against zero is one sided, and the two-sided confidence interval
is truncated at zero.

.
```

```
. estat ic

-----------------------------------------------------------------------------
       Model |    Obs    ll(null)   ll(model)     df          AIC         BIC
-------------+---------------------------------------------------------------
           . |     70           .   -2.878773     10     25.75755     48.2425
-----------------------------------------------------------------------------
               Note:  N=Obs used in calculating BIC; see [R] BIC note

.
. estimates store M29

.
.
.
. arima lnHi L7DWP L7LST if tin(2012w19, 2013w36), arima(3,1,4)

(setting optimization to BHHH)
Iteration 0:   log likelihood = -9.5936478
Iteration 1:   log likelihood = -5.7435419
Iteration 2:   log likelihood = -4.3580479
Iteration 3:   log likelihood = -4.3439393  (backed up)
Iteration 4:   log likelihood = -4.2668676
(switching optimization to BFGS)
Iteration 5:   log likelihood = -4.2450889
Iteration 6:   log likelihood = -3.8976553
Iteration 7:   log likelihood = -3.2645449
Iteration 8:   log likelihood = -3.1524962
Iteration 9:   log likelihood =  -2.972875
Iteration 10:  log likelihood = -2.9682942
Iteration 11:  log likelihood =  -2.954677
Iteration 12:  log likelihood = -2.9542148
Iteration 13:  log likelihood = -2.9540801
Iteration 14:  log likelihood = -2.9540588
(switching optimization to BHHH)
Iteration 15:  log likelihood = -2.9540568
Iteration 16:  log likelihood = -2.9540568  (backed up)
Iteration 17:  log likelihood = -2.9540568  (backed up)
Iteration 18:  log likelihood = -2.9540568  (not concave)
Iteration 19:  log likelihood = -2.9540568  (not concave)
(switching optimization to BFGS)
Iteration 20:  log likelihood = -2.9540568
Iteration 21:  log likelihood = -2.9540568  (backed up)
Iteration 22:  log likelihood = -2.9540568  (backed up)
Iteration 23:  log likelihood = -2.9540568  (backed up)
Iteration 24:  log likelihood = -2.9540568  (backed up)
Iteration 25:  log likelihood = -2.9540568  (backed up)
Iteration 26:  log likelihood = -2.9540568  (backed up)
Iteration 27:  log likelihood = -2.9540568  (backed up)
Iteration 28:  log likelihood = -2.9540568  (backed up)
Iteration 29:  log likelihood = -2.9540568

ARIMA regression

Sample:  2012w19 - 2013w36                      Number of obs      =        70
                                                Wald chi2(8)       =    163.28
Log likelihood = -2.954057                      Prob > chi2        =    0.0000

------------------------------------------------------------------------------
             |                 OPG
      D.lnHi |      Coef.   Std. Err.      z    P>|z|     [95% Conf. Interval]
-------------+----------------------------------------------------------------
lnHi         |
       L7DWP |
         D1. |   .0300535   .0084335     3.56   0.000     .0135241    .0465829
             |
       L7LST |
         D1. |   .0994312    .021805     4.56   0.000     .0566942    .1421683
             |
       _cons |  -.0204396   .0334837    -0.61   0.542    -.0860665    .0451872
-------------+----------------------------------------------------------------
```

```
ARMA         |
          ar |
         L1. |  -.8819622   .3181541    -2.77   0.006    -1.505533   -.2583916
         L2. |  -.4981217   .3207628    -1.55   0.120    -1.126805    .1305619
         L3. |  -.4766546   .1697211    -2.81   0.005    -.8093017   -.1440075
             |
          ma |
         L1. |   .5354841   3016.254     0.00   1.000    -5911.213    5912.284
         L2. |   .5791886   3700.463     0.00   1.000    -7252.195    7253.354
         L3. |   .9126311          .        .       .            .           .
         L4. |  -.1310758   56.71331    -0.00   0.998    -111.2871     111.025
-------------+----------------------------------------------------------------
      /sigma |   .2404501   52.06303     0.00   0.498            0    102.2821
------------------------------------------------------------------------------
Note: The test of the variance against zero is one sided, and the two-sided confidence interval
is truncated at zero.

.
. estat ic

-----------------------------------------------------------------------------
       Model |    Obs    ll(null)   ll(model)     df          AIC         BIC
-------------+---------------------------------------------------------------
           . |     70           .   -2.954057     10     25.90811    48.39307
-----------------------------------------------------------------------------
               Note:  N=Obs used in calculating BIC; see [R] BIC note

.
. estimates store M30

.
.
.
. arima lnHi L7DWP L7LST if tin(2012w19, 2013w36), arima(4,1,4)

(setting optimization to BHHH)
Iteration 0:   log likelihood = -7.2070807
Iteration 1:   log likelihood = -5.3696846
Iteration 2:   log likelihood = -5.2367946
Iteration 3:   log likelihood = -4.6309181
Iteration 4:   log likelihood = -4.5734749  (backed up)
(switching optimization to BFGS)
Iteration 5:   log likelihood = -4.4828996  (backed up)
Iteration 6:   log likelihood = -4.1905891  (backed up)
Iteration 7:   log likelihood =  -3.804735
Iteration 8:   log likelihood = -3.3787309
Iteration 9:   log likelihood = -3.2797686
Iteration 10:  log likelihood = -3.2360336
Iteration 11:  log likelihood = -3.1085148
Iteration 12:  log likelihood = -3.0710797
Iteration 13:  log likelihood = -3.0643017
Iteration 14:  log likelihood = -3.0593391
(switching optimization to BHHH)
Iteration 15:  log likelihood = -3.0593391  (backed up)
Iteration 16:  log likelihood = -3.0535792  (backed up)
Iteration 17:  log likelihood = -3.0286064  (backed up)
Iteration 18:  log likelihood = -3.0238365  (backed up)
Iteration 19:  log likelihood = -2.9095913  (backed up)
(switching optimization to BFGS)
Iteration 20:  log likelihood = -2.9095187  (backed up)
Iteration 21:  log likelihood = -2.8916874  (backed up)
Iteration 22:  log likelihood =  -2.653272
Iteration 23:  log likelihood = -2.2445247
Iteration 24:  log likelihood = -2.1610407
Iteration 25:  log likelihood = -1.8977748
Iteration 26:  log likelihood = -1.8135675
Iteration 27:  log likelihood = -1.7149351
Iteration 28:  log likelihood =  -1.700635
Iteration 29:  log likelihood = -1.6844307
(switching optimization to BHHH)
Iteration 30:  log likelihood = -1.6759934
```

```
Iteration 31:  log likelihood = -1.6759766  (backed up)
Iteration 32:  log likelihood = -1.6758448  (backed up)
Iteration 33:  log likelihood = -1.6758167  (backed up)
Iteration 34:  log likelihood = -1.6757714  (backed up)
(switching optimization to BFGS)
Iteration 35:  log likelihood = -1.6757658  (backed up)
Iteration 36:  log likelihood = -1.6757389  (backed up)
Iteration 37:  log likelihood = -1.6755766  (backed up)
Iteration 38:  log likelihood = -1.6748406
Iteration 39:  log likelihood = -1.6747784
Iteration 40:  log likelihood = -1.6747649
Iteration 41:  log likelihood = -1.6747623
Iteration 42:  log likelihood = -1.6747613
Iteration 43:  log likelihood = -1.6747613

ARIMA regression

Sample:  2012w19 - 2013w36                        Number of obs      =        70
                                                  Wald chi2(8)       =     75.64
Log likelihood = -1.674761                        Prob > chi2        =    0.0000

------------------------------------------------------------------------------
             |                 OPG
      D.lnHi |      Coef.   Std. Err.      z    P>|z|     [95% Conf. Interval]
-------------+----------------------------------------------------------------
lnHi         |
       L7DWP |
         D1. |   .0312037   .0083254     3.75   0.000     .0148862    .0475212
             |
       L7LST |
         D1. |   .1025752   .0203534     5.04   0.000     .0626834    .1424671
             |
       _cons |  -.0126904   .0109687    -1.16   0.247    -.0341887    .0088078
-------------+----------------------------------------------------------------
ARMA         |
          ar |
         L1. |   -.075203   .1420006    -0.53   0.596     -.353519     .203113
         L2. |   .2982293   .1736459     1.72   0.086    -.0421104     .638569
         L3. |  -.0094616    .165471    -0.06   0.954    -.3337788    .3148556
         L4. |   .3916599   .1723319     2.27   0.023     .0538957    .7294242
             |
          ma |
         L1. |  -.3384826   .1544162    -2.19   0.028    -.6411327   -.0358324
         L2. |  -4.96e-07   36201.89    -0.00   1.000     -70954.4     70954.4
         L3. |   .3384828          .        .       .            .           .
         L4. |  -.9999996          .        .       .            .           .
-------------+----------------------------------------------------------------
      /sigma |   .2330033   .0266203     8.75   0.000     .1808285     .285178
------------------------------------------------------------------------------
Note: The test of the variance against zero is one sided, and the two-sided confidence interval
is truncated at zero.

.
. estat ic

-----------------------------------------------------------------------------
       Model |    Obs    ll(null)   ll(model)     df          AIC         BIC
-------------+---------------------------------------------------------------
           . |     70           .   -1.674761     10     23.34952    45.83448
-----------------------------------------------------------------------------
               Note:  N=Obs used in calculating BIC; see [R] BIC note

.
. estimates store M31

.
.
.
. arima lnHi L7DWP L7LST if tin(2012w19, 2013w36), arima(5,1,4)

(setting optimization to BHHH)
```

```
Iteration 0:   log likelihood = -8.9036424
Iteration 1:   log likelihood = -4.5839343
Iteration 2:   log likelihood = -4.1481359
Iteration 3:   log likelihood = -3.4362395  (not concave)
Iteration 4:   log likelihood = -2.7213308
(switching optimization to BFGS)
Iteration 5:   log likelihood = -2.7196207  (backed up)
Iteration 6:   log likelihood = -2.6951166  (backed up)
Iteration 7:   log likelihood = -2.3259387  (backed up)
Iteration 8:   log likelihood = -2.1663108
Iteration 9:   log likelihood =  -2.121233
Iteration 10:  log likelihood =  -2.089272
Iteration 11:  log likelihood = -1.9901325
Iteration 12:  log likelihood = -1.8198283
Iteration 13:  log likelihood = -1.6375388
Iteration 14:  log likelihood = -1.4487008
(switching optimization to BHHH)
Iteration 15:  log likelihood = -1.4439771
Iteration 16:  log likelihood = -1.4057584  (backed up)
Iteration 17:  log likelihood = -1.3757044  (backed up)
Iteration 18:  log likelihood =  -1.356203  (backed up)
Iteration 19:  log likelihood = -1.2648898  (backed up)
(switching optimization to BFGS)
Iteration 20:  log likelihood = -1.2643286  (backed up)
Iteration 21:  log likelihood = -1.2482869
Iteration 22:  log likelihood = -1.1828283  (backed up)
Iteration 23:  log likelihood = -.96902274
Iteration 24:  log likelihood = -.91887682
Iteration 25:  log likelihood = -.90982101
Iteration 26:  log likelihood = -.90688745
Iteration 27:  log likelihood = -.90593678
Iteration 28:  log likelihood = -.90553687
Iteration 29:  log likelihood =  -.9054921
(switching optimization to BHHH)
Iteration 30:  log likelihood = -.90549031  (not concave)
Iteration 31:  log likelihood =  -.9054903  (not concave)
Iteration 32:  log likelihood = -.90549027  (not concave)
Iteration 33:  log likelihood = -.90549027  (not concave)
Iteration 34:  log likelihood = -.90549027  (not concave)
(switching optimization to BFGS)
Iteration 35:  log likelihood = -.90549027

ARIMA regression

Sample:  2012w19 - 2013w36                      Number of obs      =        70
                                                Wald chi2(9)       =  13211.59
Log likelihood = -.9054903                      Prob > chi2        =    0.0000

------------------------------------------------------------------------------
             |                 OPG
      D.lnHi |      Coef.   Std. Err.      z    P>|z|     [95% Conf. Interval]
-------------+----------------------------------------------------------------
lnHi         |
       L7DWP |
         D1. |   .0277491   .0069697     3.98   0.000     .0140887    .0414096
             |
       L7LST |
         D1. |   .0986595   .0204539     4.82   0.000     .0585706    .1387485
             |
       _cons |  -.0219581   .0352996    -0.62   0.534    -.0911441    .0472278
-------------+----------------------------------------------------------------
ARMA         |
          ar |
         L1. |  -1.921513   .1974089    -9.73   0.000    -2.308427   -1.534599
         L2. |   -1.53587   .3792623    -4.05   0.000     -2.27921   -.7925291
         L3. |  -1.111792   .3800994    -2.93   0.003    -1.856773   -.3668112
         L4. |   -.506518   .3122983    -1.62   0.105    -1.118611    .1055754
         L5. |   .0254745   .1471463     0.17   0.863     -.262927     .313876
             |
          ma |
         L1. |   1.677391          .        .       .            .           .
```

```
         L2. |   1.361685    .120553    11.30   0.000     1.125405    1.597964
         L3. |   1.677416          .        .       .            .           .
         L4. |   1.000007   .1156127     8.65   0.000     .7734101    1.226604
-------------+----------------------------------------------------------------
      /sigma |   .2270149   .0300544     7.55   0.000     .1681093    .2859205
------------------------------------------------------------------------------
Note: The test of the variance against zero is one sided, and the two-sided confidence interval
is truncated at zero.

.
. estat ic

-----------------------------------------------------------------------------
       Model |    Obs    ll(null)   ll(model)     df          AIC         BIC
-------------+---------------------------------------------------------------
           . |     70           .   -.9054903     11     23.81098    48.54443
-----------------------------------------------------------------------------
               Note:  N=Obs used in calculating BIC; see [R] BIC note

.
. estimates store M32

.
.
.
. arima lnHi L7DWP L7LST if tin(2012w19, 2013w36), arima(4,1,5)

(setting optimization to BHHH)
Iteration 0:   log likelihood = -7.4439904
Iteration 1:   log likelihood = -5.5089573
Iteration 2:   log likelihood =  -4.199391
Iteration 3:   log likelihood = -3.9624919
Iteration 4:   log likelihood = -3.0272778
(switching optimization to BFGS)
Iteration 5:   log likelihood = -2.8405881
Iteration 6:   log likelihood =  -2.376467
Iteration 7:   log likelihood = -1.9627229
Iteration 8:   log likelihood = -1.7794883
Iteration 9:   log likelihood = -1.7033051
Iteration 10:  log likelihood = -1.5869252
Iteration 11:  log likelihood = -1.5184721
Iteration 12:  log likelihood = -1.1863389
Iteration 13:  log likelihood =   -.896037
Iteration 14:  log likelihood = -.88254978  (backed up)
(switching optimization to BHHH)
Iteration 15:  log likelihood = -.77298708
Iteration 16:  log likelihood = -.77145458  (backed up)
Iteration 17:  log likelihood = -.76996278  (backed up)
Iteration 18:  log likelihood = -.73759144  (backed up)
Iteration 19:  log likelihood = -.73424362  (backed up)
(switching optimization to BFGS)
Iteration 20:  log likelihood = -.72963335  (backed up)
Iteration 21:  log likelihood = -.67415944
Iteration 22:  log likelihood = -.56587957
Iteration 23:  log likelihood = -.39309154
Iteration 24:  log likelihood = -.34785039
Iteration 25:  log likelihood = -.33833904
Iteration 26:  log likelihood = -.33208735
Iteration 27:  log likelihood = -.33112397
Iteration 28:  log likelihood = -.32714911
Iteration 29:  log likelihood = -.32492804
(switching optimization to BHHH)
Iteration 30:  log likelihood = -.32456464
Iteration 31:  log likelihood = -.32456435  (backed up)
Iteration 32:  log likelihood = -.32456414  (backed up)
Iteration 33:  log likelihood = -.32455832  (backed up)
Iteration 34:  log likelihood = -.32455626  (backed up)
(switching optimization to BFGS)
Iteration 35:  log likelihood =   -.324556  (backed up)
Iteration 36:  log likelihood =  -.3245536  (backed up)
Iteration 37:  log likelihood = -.32444203
```

```
Iteration 38:  log likelihood = -.32443475
Iteration 39:  log likelihood = -.32439141
Iteration 40:  log likelihood = -.32438432
Iteration 41:  log likelihood =   -.324381
Iteration 42:  log likelihood = -.32438043
Iteration 43:  log likelihood = -.32437992
Iteration 44:  log likelihood =  -.3243799

ARIMA regression

Sample:  2012w19 - 2013w36                        Number of obs      =        70
                                                  Wald chi2(9)       =  33510.55
Log likelihood = -.3243799                        Prob > chi2        =    0.0000

------------------------------------------------------------------------------
             |                 OPG
      D.lnHi |      Coef.   Std. Err.      z    P>|z|     [95% Conf. Interval]
-------------+----------------------------------------------------------------
lnHi         |
       L7DWP |
         D1. |   .0316902   .0115037     2.75   0.006     .0091434     .054237
             |
       L7LST |
         D1. |   .1069342   .0236404     4.52   0.000     .0605998    .1532686
             |
       _cons |  -.0155474   .0406968    -0.38   0.702    -.0953116    .0642168
-------------+----------------------------------------------------------------
ARMA         |
          ar |
         L1. |  -1.471874   .2388755    -6.16   0.000    -1.940061   -1.003686
         L2. |  -1.636725   .3232579    -5.06   0.000    -2.270299   -1.003151
         L3. |  -1.194314   .3183322    -3.75   0.000    -1.818234   -.5703949
         L4. |   -.236936   .2369856    -1.00   0.317    -.7014192    .2275472
             |
          ma |
         L1. |   1.128897   .2103231     5.37   0.000     .7166709    1.541122
         L2. |   2.501421          .        .       .            .           .
         L3. |   2.698448          .        .       .            .           .
         L4. |   1.811045    .273559     6.62   0.000      1.27488    2.347211
         L5. |   1.485123   .6457317     2.30   0.021     .2195118    2.750733
-------------+----------------------------------------------------------------
      /sigma |   .1535425   .0309318     4.96   0.000     .0929172    .2141677
------------------------------------------------------------------------------
Note: The test of the variance against zero is one sided, and the two-sided confidence interval
is truncated at zero.

.
. estat ic

-----------------------------------------------------------------------------
       Model |    Obs    ll(null)   ll(model)     df          AIC         BIC
-------------+---------------------------------------------------------------
           . |     70           .   -.3243799     11     22.64876    47.38221
-----------------------------------------------------------------------------
               Note:  N=Obs used in calculating BIC; see [R] BIC note

.
. estimates store M33

.
.
.
. arima lnHi L7DWP L7LST if tin(2012w19, 2013w36), arima(5,1,5)

(setting optimization to BHHH)
Iteration 0:   log likelihood = -8.8223069
Iteration 1:   log likelihood = -5.1029509
Iteration 2:   log likelihood = -3.7090544
Iteration 3:   log likelihood = -3.6425884  (backed up)
Iteration 4:   log likelihood = -3.0759893
(switching optimization to BFGS)
```

```
Iteration 5:   log likelihood = -3.0584781  (backed up)
Iteration 6:   log likelihood = -2.6118809  (backed up)
Iteration 7:   log likelihood = -2.5646984  (backed up)
Iteration 8:   log likelihood = -2.4513116
Iteration 9:   log likelihood = -2.0669599
Iteration 10:  log likelihood = -1.9138389
Iteration 11:  log likelihood =  -1.464224
Iteration 12:  log likelihood = -1.3966835  (backed up)
Iteration 13:  log likelihood = -1.0907508
Iteration 14:  log likelihood = -1.0340787
(switching optimization to BHHH)
Iteration 15:  log likelihood = -.89440802
Iteration 16:  log likelihood = -.86344768  (backed up)
Iteration 17:  log likelihood = -.77235633
Iteration 18:  log likelihood = -.70756281
Iteration 19:  log likelihood = -.70482906
(switching optimization to BFGS)
Iteration 20:  log likelihood = -.69431773  (backed up)
Iteration 21:  log likelihood = -.55889778
Iteration 22:  log likelihood = -.35556813
Iteration 23:  log likelihood = -.34697315
Iteration 24:  log likelihood = -.17703328
Iteration 25:  log likelihood =  .08651624
Iteration 26:  log likelihood =  .10457581
Iteration 27:  log likelihood =  .12218854
Iteration 28:  log likelihood =  .13383754
Iteration 29:  log likelihood =  .13549681
(switching optimization to BHHH)
Iteration 30:  log likelihood =  .13578045
Iteration 31:  log likelihood =  .13578065  (backed up)
Iteration 32:  log likelihood =  .13578227  (backed up)
Iteration 33:  log likelihood =  .13578268  (backed up)
Iteration 34:  log likelihood =  .13578347  (backed up)
(switching optimization to BFGS)
Iteration 35:  log likelihood =  .13578348  (backed up)
Iteration 36:  log likelihood =  .13578371  (backed up)
Iteration 37:  log likelihood =  .13591387  (backed up)
Iteration 38:  log likelihood =  .13643989
Iteration 39:  log likelihood =  .13666104
Iteration 40:  log likelihood =  .13681918
Iteration 41:  log likelihood =  .13682272
Iteration 42:  log likelihood =  .13683159
Iteration 43:  log likelihood =  .13683246
Iteration 44:  log likelihood =  .13683283
(switching optimization to BHHH)
Iteration 45:  log likelihood =  .13683286  (not concave)
Iteration 46:  log likelihood =  .13683286  (not concave)
Iteration 47:  log likelihood =  .13683287  (not concave)
Iteration 48:  log likelihood =  .13683287  (not concave)
Iteration 49:  log likelihood =  .13683287  (not concave)
(switching optimization to BFGS)
Iteration 50:  log likelihood =  .13683287

ARIMA regression

Sample:  2012w19 - 2013w36                      Number of obs      =        70
                                                Wald chi2(9)       =   3311.61
Log likelihood =  .1368329                      Prob > chi2        =    0.0000

------------------------------------------------------------------------------
             |                 OPG
      D.lnHi |      Coef.   Std. Err.      z    P>|z|     [95% Conf. Interval]
-------------+----------------------------------------------------------------
lnHi         |
       L7DWP |
         D1. |   .0329279   .0080372     4.10   0.000     .0171754    .0486805
             |
       L7LST |
         D1. |   .0829415   .0195379     4.25   0.000     .0446479    .1212351
             |
       _cons |  -.0191424   .0401045    -0.48   0.633    -.0977458    .0594609
```

```
-------------+----------------------------------------------------------------
ARMA         |
          ar |
         L1. |  -1.439751   .2082432    -6.91   0.000      -1.8479   -1.031601
         L2. |  -1.822584   .3419752    -5.33   0.000    -2.492843   -1.152325
         L3. |  -1.496998   .3962825    -3.78   0.000    -2.273698    -.720299
         L4. |  -.5517668   .3253167    -1.70   0.090    -1.189376    .0858422
         L5. |  -.2793657   .1620035    -1.72   0.085    -.5968867    .0381554
             |
          ma |
         L1. |   1.159222   .1290978     8.98   0.000     .9061946    1.412249
         L2. |   2.006867          .        .       .            .           .
         L3. |   2.006866          .        .       .            .           .
         L4. |   1.159221          .        .       .            .           .
         L5. |   .9999988   .1520516     6.58   0.000     .7019833    1.298014
-------------+----------------------------------------------------------------
      /sigma |   .2227257   .0326689     6.82   0.000     .1586958    .2867557
------------------------------------------------------------------------------
Note: The test of the variance against zero is one sided, and the two-sided confidence interval
is truncated at zero.

.
. estat ic

-----------------------------------------------------------------------------
       Model |    Obs    ll(null)   ll(model)     df          AIC         BIC
-------------+---------------------------------------------------------------
           . |     70           .    .1368329     11     21.72633    46.45978
-----------------------------------------------------------------------------
               Note:  N=Obs used in calculating BIC; see [R] BIC note

.
. estimates store M34

.
.
.
.
.
. arima lnHi L7DWP L7LST if tin(2012w19, 2013w36), arima(6,1,5)

(setting optimization to BHHH)
Iteration 0:   log likelihood = -9.7314096
Iteration 1:   log likelihood = -4.9454522
Iteration 2:   log likelihood = -3.4487411
Iteration 3:   log likelihood = -3.3578193
Iteration 4:   log likelihood = -3.3576536  (not concave)
(switching optimization to BFGS)
Iteration 5:   log likelihood = -1.0563238
Iteration 6:   log likelihood = -.81445442
Iteration 7:   log likelihood = -.44125328
Iteration 8:   log likelihood = -.32520169
Iteration 9:   log likelihood =   -.284345
Iteration 10:  log likelihood = -.28112462
Iteration 11:  log likelihood = -.25233357
Iteration 12:  log likelihood = -.22425836
Iteration 13:  log likelihood = -.18765604
Iteration 14:  log likelihood = -.14910609
(switching optimization to BHHH)
Iteration 15:  log likelihood = -.11403508
Iteration 16:  log likelihood = -.11363289  (backed up)
Iteration 17:  log likelihood = -.11363235  (backed up)
Iteration 18:  log likelihood = -.11363105  (backed up)
Iteration 19:  log likelihood = -.11362552  (backed up)
(switching optimization to BFGS)
Iteration 20:  log likelihood = -.11354671  (backed up)
Iteration 21:  log likelihood = -.11331772  (backed up)
Iteration 22:  log likelihood = -.11279594
Iteration 23:  log likelihood = -.09995428
Iteration 24:  log likelihood = -.09830908
Iteration 25:  log likelihood = -.09694938
```

```
Iteration 26:  log likelihood = -.09642456
Iteration 27:  log likelihood = -.09571922
Iteration 28:  log likelihood = -.09564546
Iteration 29:  log likelihood = -.09563555
(switching optimization to BHHH)
Iteration 30:  log likelihood = -.09563344  (not concave)
Iteration 31:  log likelihood = -.09563308
Iteration 32:  log likelihood = -.09563302  (backed up)
Iteration 33:  log likelihood = -.09563298  (backed up)
Iteration 34:  log likelihood = -.09563298  (not concave)
(switching optimization to BFGS)
Iteration 35:  log likelihood =  -.0956329
Iteration 36:  log likelihood = -.09563289  (backed up)
Iteration 37:  log likelihood = -.09563286  (backed up)
Iteration 38:  log likelihood = -.09563286  (backed up)
Iteration 39:  log likelihood = -.09563286  (backed up)
Iteration 40:  log likelihood = -.09563286  (backed up)
Iteration 41:  log likelihood = -.09563286  (backed up)
Iteration 42:  log likelihood = -.09563286  (backed up)
Iteration 43:  log likelihood = -.09563286  (backed up)
Iteration 44:  log likelihood = -.09563286  (backed up)
(switching optimization to BHHH)
Iteration 45:  log likelihood = -.09563286  (backed up)
Iteration 46:  log likelihood = -.09563286  (not concave)
Iteration 47:  log likelihood = -.09563285  (not concave)
Iteration 48:  log likelihood = -.09563285  (not concave)
Iteration 49:  log likelihood = -.09563285  (not concave)
(switching optimization to BFGS)
Iteration 50:  log likelihood = -.09563285

ARIMA regression

Sample:  2012w19 - 2013w36                      Number of obs      =        70
                                                Wald chi2(10)      =    127.73
Log likelihood = -.0956328                      Prob > chi2        =    0.0000

------------------------------------------------------------------------------
             |                 OPG
      D.lnHi |      Coef.   Std. Err.      z    P>|z|     [95% Conf. Interval]
-------------+----------------------------------------------------------------
lnHi         |
       L7DWP |
         D1. |    .031413   .0120516     2.61   0.009     .0077922    .0550337
             |
       L7LST |
         D1. |   .1212937   .0270972     4.48   0.000     .0681842    .1744032
             |
       _cons |  -.0085381   .0075858    -1.13   0.260    -.0234061    .0063299
-------------+----------------------------------------------------------------
ARMA         |
          ar |
         L1. |  -.1088023   .4293419    -0.25   0.800    -.9502969    .7326923
         L2. |   .2908357   .1835527     1.58   0.113    -.0689209    .6505923
         L3. |   .1937089   .1332567     1.45   0.146    -.0674695    .4548873
         L4. |   .5850845   .1364439     4.29   0.000     .3176594    .8525096
         L5. |  -.1856658   .2702609    -0.69   0.492    -.7153675    .3440359
         L6. |  -.3550061   .1461252    -2.43   0.015    -.6414063    -.068606
             |
          ma |
         L1. |  -.3058928          .        .       .            .           .
         L2. |   .0123934          .        .       .            .           .
         L3. |   .0480795   .4593642     0.10   0.917    -.8522578    .9484168
         L4. |  -1.012396          .        .       .            .           .
         L5. |    .257816   .4568874     0.56   0.573    -.6376668    1.153299
-------------+----------------------------------------------------------------
      /sigma |   .2254344   .0278798     8.09   0.000     .1707911    .2800777
------------------------------------------------------------------------------
Note: The test of the variance against zero is one sided, and the two-sided confidence interval
is truncated at zero.

.
```

```
. estat ic

-----------------------------------------------------------------------------
       Model |    Obs    ll(null)   ll(model)     df          AIC         BIC
-------------+---------------------------------------------------------------
           . |     70           .   -.0956328     12     24.19127    51.17321
-----------------------------------------------------------------------------
               Note:  N=Obs used in calculating BIC; see [R] BIC note

.
. estimates store M35

.
.
.
.
.
. arima lnHi L7DWP L7LST if tin(2012w19, 2013w36), arima(6,1,6)

(setting optimization to BHHH)
Iteration 0:   log likelihood = -10.484445
Iteration 1:   log likelihood = -5.7755702
Iteration 2:   log likelihood = -5.7541991  (backed up)
Iteration 3:   log likelihood = -5.7534724  (backed up)
Iteration 4:   log likelihood = -5.7534681  (backed up)
(switching optimization to BFGS)
Iteration 5:   log likelihood = -5.7534677  (backed up)
Iteration 6:   log likelihood = -3.9819497
Iteration 7:   log likelihood = -3.8694801
Iteration 8:   log likelihood = -1.4851995
Iteration 9:   log likelihood = -.08461663
Iteration 10:  log likelihood =  .26252501  (backed up)
Iteration 11:  log likelihood =  .75570134
Iteration 12:  log likelihood =  1.1154924
Iteration 13:  log likelihood =  1.3344716
Iteration 14:  log likelihood =  1.3938502
(switching optimization to BHHH)
Iteration 15:  log likelihood =  1.4889979
Iteration 16:  log likelihood =  1.4890462  (backed up)
Iteration 17:  log likelihood =  1.4891336  (backed up)
Iteration 18:  log likelihood =  1.4891551  (backed up)
Iteration 19:  log likelihood =  1.4891602  (backed up)
(switching optimization to BFGS)
Iteration 20:  log likelihood =  1.4891704  (backed up)
Iteration 21:  log likelihood =  1.4891784  (backed up)
Iteration 22:  log likelihood =  1.7737806
Iteration 23:  log likelihood =  1.8476384
Iteration 24:  log likelihood =  2.0209928
Iteration 25:  log likelihood =  2.0787505
Iteration 26:  log likelihood =  2.1173627
Iteration 27:  log likelihood =  2.1363736
Iteration 28:  log likelihood =  2.1411076
Iteration 29:  log likelihood =  2.1440631
(switching optimization to BHHH)
Iteration 30:  log likelihood =  2.1444922
Iteration 31:  log likelihood =  2.1444923  (backed up)
Iteration 32:  log likelihood =  2.1444923  (backed up)
Iteration 33:  log likelihood =  2.1444933  (backed up)
Iteration 34:  log likelihood =  2.1444944  (backed up)
(switching optimization to BFGS)
Iteration 35:  log likelihood =   2.144499  (backed up)
Iteration 36:  log likelihood =  2.1445002  (backed up)
Iteration 37:  log likelihood =  2.1445004  (backed up)
Iteration 38:  log likelihood =   2.144583
Iteration 39:  log likelihood =  2.1445909
Iteration 40:  log likelihood =  2.1446312
Iteration 41:  log likelihood =  2.1446345
Iteration 42:  log likelihood =  2.1446354
Iteration 43:  log likelihood =  2.1446357

ARIMA regression
```

```
Sample:  2012w19 - 2013w36                      Number of obs      =        70
                                                Wald chi2(11)      =  28055.69
Log likelihood =  2.144636                      Prob > chi2        =    0.0000

------------------------------------------------------------------------------
             |                 OPG
      D.lnHi |      Coef.   Std. Err.      z    P>|z|     [95% Conf. Interval]
-------------+----------------------------------------------------------------
lnHi         |
       L7DWP |
         D1. |   .0323895   .0143801     2.25   0.024     .0042051     .060574
             |
       L7LST |
         D1. |   .1065709   .0249831     4.27   0.000      .057605    .1555368
             |
       _cons |  -.0101065   .0100923    -1.00   0.317    -.0298871    .0096741
-------------+----------------------------------------------------------------
ARMA         |
          ar |
         L1. |  -.5466847   .2586152    -2.11   0.035    -1.053561   -.0398083
         L2. |  -.4334176   .2632515    -1.65   0.100    -.9493811    .0825459
         L3. |   .1234847   .1538215     0.80   0.422    -.1779999    .4249693
         L4. |   .6958297   .1827636     3.81   0.000     .3376197     1.05404
         L5. |   .0939592   .2931313     0.32   0.749    -.4805676    .6684861
         L6. |   .0387682   .2495115     0.16   0.877    -.4502654    .5278017
             |
          ma |
         L1. |   .1859132   .2340348     0.79   0.427    -.2727865    .6446129
         L2. |   .6804413          .        .       .            .           .
         L3. |   -.093096          .        .       .            .           .
         L4. |   -.903964          .        .       .            .           .
         L5. |  -.0928176   .2243692    -0.41   0.679    -.5325731    .3469379
         L6. |  -.7764773   .1503228    -5.17   0.000    -1.071105     -.48185
-------------+----------------------------------------------------------------
      /sigma |   .2174904   .0273259     7.96   0.000     .1639327    .2710481
------------------------------------------------------------------------------
Note: The test of the variance against zero is one sided, and the two-sided confidence interval
is truncated at zero.

.
. estat ic

-----------------------------------------------------------------------------
       Model |    Obs    ll(null)   ll(model)     df          AIC         BIC
-------------+---------------------------------------------------------------
           . |     70           .    2.144636     13     21.71073    50.94117
-----------------------------------------------------------------------------
               Note:  N=Obs used in calculating BIC; see [R] BIC note

.
. estimates store M36

.
.
.
. arima lnHi L7DWP L7LST L5NDVI L7NDVI if tin(2012w19, 2013w36), arima(6,1,5)

(setting optimization to BHHH)
Iteration 0:   log likelihood = -9.0988403
Iteration 1:   log likelihood =  -3.753652
Iteration 2:   log likelihood = -3.6865272
Iteration 3:   log likelihood = -2.3544431
Iteration 4:   log likelihood =  .17834954
(switching optimization to BFGS)
Iteration 5:   log likelihood =  1.6714045
Iteration 6:   log likelihood =  2.0529322
Iteration 7:   log likelihood =    2.82479
Iteration 8:   log likelihood =  2.9236373
Iteration 9:   log likelihood =  4.5847295
Iteration 10:  log likelihood =  5.2326107
```

```
Iteration 11:  log likelihood =  5.5099667
Iteration 12:  log likelihood =  5.8557078
Iteration 13:  log likelihood =  5.8918975
Iteration 14:  log likelihood =  5.9022964
(switching optimization to BHHH)
Iteration 15:  log likelihood =  6.4127676
Iteration 16:  log likelihood =   6.548209
Iteration 17:  log likelihood =  6.5610617  (backed up)
Iteration 18:  log likelihood =  6.6050767  (backed up)
Iteration 19:  log likelihood =  6.6098056  (backed up)
(switching optimization to BFGS)
Iteration 20:  log likelihood =   6.610081  (backed up)
Iteration 21:  log likelihood =  6.7251534
Iteration 22:  log likelihood =  7.0449468
Iteration 23:  log likelihood =  7.0930408
Iteration 24:  log likelihood =  7.1830244
Iteration 25:  log likelihood =  7.2536584
Iteration 26:  log likelihood =  7.2870306
Iteration 27:  log likelihood =  7.3172958
Iteration 28:  log likelihood =  7.3189367
Iteration 29:  log likelihood =  7.3249916
(switching optimization to BHHH)
Iteration 30:  log likelihood =  7.3280113
Iteration 31:  log likelihood =  7.3280115  (not concave)
Iteration 32:  log likelihood =  7.3285108
Iteration 33:  log likelihood =  7.3285109  (backed up)
Iteration 34:  log likelihood =   7.328511  (backed up)
(switching optimization to BFGS)
Iteration 35:  log likelihood =  7.3285111  (backed up)
Iteration 36:  log likelihood =  7.3285658  (backed up)
Iteration 37:  log likelihood =  7.3287023  (backed up)
Iteration 38:  log likelihood =  7.3288782
Iteration 39:  log likelihood =  7.3289983
Iteration 40:  log likelihood =   7.329146
Iteration 41:  log likelihood =  7.3291738
Iteration 42:  log likelihood =  7.3291826
Iteration 43:  log likelihood =  7.3291842
Iteration 44:  log likelihood =  7.3291848

ARIMA regression

Sample:  2012w19 - 2013w36                      Number of obs      =        70
                                                Wald chi2(12)      =   2332.22
Log likelihood =  7.329185                      Prob > chi2        =    0.0000

------------------------------------------------------------------------------
             |                 OPG
      D.lnHi |      Coef.   Std. Err.      z    P>|z|     [95% Conf. Interval]
-------------+----------------------------------------------------------------
lnHi         |
       L7DWP |
         D1. |   .0325667   .0122657     2.66   0.008     .0085264     .056607
             |
       L7LST |
         D1. |   .1404217   .0171067     8.21   0.000     .1068931    .1739502
             |
      L5NDVI |
         D1. |   .8278705   .6310043     1.31   0.190    -.4088752    2.064616
             |
      L7NDVI |
         D1. |   1.384557   .4872082     2.84   0.004     .4296462    2.339467
             |
       _cons |  -.0095866   .0016796    -5.71   0.000    -.0128786   -.0062947
-------------+----------------------------------------------------------------
ARMA         |
          ar |
         L1. |   .1528434   .1979723     0.77   0.440    -.2351752    .5408619
         L2. |   .4104153   .1342139     3.06   0.002     .1473608    .6734698
         L3. |   .1320319   .1658664     0.80   0.426    -.1930603    .4571241
         L4. |    .531294   .1102072     4.82   0.000      .315292    .7472961
         L5. |  -.4528669   .1481085    -3.06   0.002    -.7431543   -.1625796
```

```
         L6. |  -.4063789   .1776436    -2.29   0.022     -.754554   -.0582039
             |
          ma |
         L1. |  -.9769594          .        .       .            .           .
         L2. |  -.0166803          .        .       .            .           .
         L3. |   -.016698    .224815    -0.07   0.941    -.4573273    .4239313
         L4. |  -.9769506   .2143595    -4.56   0.000    -1.397087   -.5568138
         L5. |   .9999984          .        .       .            .           .
-------------+----------------------------------------------------------------
      /sigma |   .1948034   .0325176     5.99   0.000     .1310701    .2585367
------------------------------------------------------------------------------
Note: The test of the variance against zero is one sided, and the two-sided confidence interval
is truncated at zero.

.
. estat ic

-----------------------------------------------------------------------------
       Model |    Obs    ll(null)   ll(model)     df          AIC         BIC
-------------+---------------------------------------------------------------
           . |     70           .    7.329185     14     13.34163    44.82056
-----------------------------------------------------------------------------
               Note:  N=Obs used in calculating BIC; see [R] BIC note

.
. estimates store M37

.
.
.
. arima lnHi L7DWP L7LST L5NDVI L7NDVI if tin(2012w19, 2013w36), arima(5,1,5)

(setting optimization to BHHH)
Iteration 0:   log likelihood = -8.6259497
Iteration 1:   log likelihood = -6.9531401
Iteration 2:   log likelihood = -6.8971663
Iteration 3:   log likelihood = -6.2995247
Iteration 4:   log likelihood = -5.1545915
(switching optimization to BFGS)
Iteration 5:   log likelihood = -1.8839926
Iteration 6:   log likelihood = -1.8603221  (backed up)
Iteration 7:   log likelihood = -1.7920547  (backed up)
Iteration 8:   log likelihood = -.43751299
Iteration 9:   log likelihood =  .00740859
Iteration 10:  log likelihood =  .42376019
Iteration 11:  log likelihood =  .67841773
Iteration 12:  log likelihood =  .93662865
Iteration 13:  log likelihood =  1.1373631
Iteration 14:  log likelihood =  1.3448334
(switching optimization to BHHH)
Iteration 15:  log likelihood =  1.6684655
Iteration 16:  log likelihood =  1.6686229  (backed up)
Iteration 17:  log likelihood =  1.6697304  (backed up)
Iteration 18:  log likelihood =  1.6732697  (backed up)
Iteration 19:  log likelihood =   1.676284  (backed up)
(switching optimization to BFGS)
Iteration 20:  log likelihood =  1.6790581  (backed up)
Iteration 21:  log likelihood =  1.6860438
Iteration 22:  log likelihood =  2.0392591
Iteration 23:  log likelihood =  2.0732926
Iteration 24:  log likelihood =  2.2746783
Iteration 25:  log likelihood =  2.3206486
Iteration 26:  log likelihood =    2.34465
Iteration 27:  log likelihood =  2.3500975
Iteration 28:  log likelihood =  2.3641456
Iteration 29:  log likelihood =  2.3657178
(switching optimization to BHHH)
Iteration 30:  log likelihood =  2.3659759  (not concave)
Iteration 31:  log likelihood =  2.3660538  (not concave)
Iteration 32:  log likelihood =  2.3660745  (not concave)
Iteration 33:  log likelihood =  2.3660851  (not concave)
```

```
Iteration 34:  log likelihood =   2.366091  (not concave)
(switching optimization to BFGS)
Iteration 35:  log likelihood =  2.3660976
Iteration 36:  log likelihood =  2.3661251  (backed up)
Iteration 37:  log likelihood =  2.3661274  (backed up)
Iteration 38:  log likelihood =  2.3661278  (backed up)
Iteration 39:  log likelihood =  2.3661283  (backed up)
Iteration 40:  log likelihood =  2.3661286  (backed up)
Iteration 41:  log likelihood =  2.3661294  (backed up)
Iteration 42:  log likelihood =  2.3661295  (backed up)
Iteration 43:  log likelihood =  2.3661297  (backed up)
Iteration 44:  log likelihood =  2.3661298  (backed up)
(switching optimization to BHHH)
Iteration 45:  log likelihood =  2.3661308  (backed up)
Iteration 46:  log likelihood =  2.3661314  (backed up)
Iteration 47:  log likelihood =  2.3661317  (not concave)
Iteration 48:  log likelihood =  2.3661364  (not concave)
Iteration 49:  log likelihood =  2.3661389  (not concave)
(switching optimization to BFGS)
Iteration 50:  log likelihood =  2.3661412
Iteration 51:  log likelihood =  2.3661648  (backed up)
Iteration 52:  log likelihood =   2.366166  (backed up)
Iteration 53:  log likelihood =  2.3661663  (backed up)
Iteration 54:  log likelihood =  2.3661664  (backed up)
Iteration 55:  log likelihood =  2.3661664  (backed up)
Iteration 56:  log likelihood =  2.3661667  (backed up)
Iteration 57:  log likelihood =  2.3661675  (backed up)
Iteration 58:  log likelihood =  2.3661676  (backed up)
Iteration 59:  log likelihood =  2.3661679  (backed up)
(switching optimization to BHHH)
Iteration 60:  log likelihood =  2.3661682  (not concave)
Iteration 61:  log likelihood =  2.3661684  (not concave)
Iteration 62:  log likelihood =  2.3661685  (not concave)
Iteration 63:  log likelihood =  2.3661687  (not concave)
Iteration 64:  log likelihood =  2.3661688  (not concave)
(switching optimization to BFGS)
Iteration 65:  log likelihood =  2.3661689
Iteration 66:  log likelihood =  2.3661689  (backed up)
Iteration 67:  log likelihood =   2.366169  (backed up)
Iteration 68:  log likelihood =  2.3661691  (backed up)
Iteration 69:  log likelihood =  2.3661691  (backed up)
Iteration 70:  log likelihood =  2.3661691  (backed up)
Iteration 71:  log likelihood =  2.3661692  (backed up)
Iteration 72:  log likelihood =  2.3661692  (backed up)
Iteration 73:  log likelihood =  2.3661692  (backed up)
Iteration 74:  log likelihood =  2.3661692  (backed up)
(switching optimization to BHHH)
Iteration 75:  log likelihood =  2.3661692  (not concave)
Iteration 76:  log likelihood =  2.3661693  (not concave)
Iteration 77:  log likelihood =  2.3661693  (not concave)
Iteration 78:  log likelihood =  2.3661693  (not concave)
Iteration 79:  log likelihood =  2.3661693  (not concave)
(switching optimization to BFGS)
Iteration 80:  log likelihood =  2.3661693
Iteration 81:  log likelihood =  2.3661693  (backed up)
Iteration 82:  log likelihood =  2.3661694  (backed up)
Iteration 83:  log likelihood =  2.3661694  (backed up)
Iteration 84:  log likelihood =  2.3661694  (backed up)
Iteration 85:  log likelihood =  2.3661694  (backed up)
Iteration 86:  log likelihood =  2.3661694  (backed up)
Iteration 87:  log likelihood =  2.3661694  (backed up)
Iteration 88:  log likelihood =  2.3661694  (backed up)
Iteration 89:  log likelihood =  2.3661694  (backed up)
(switching optimization to BHHH)
Iteration 90:  log likelihood =  2.3661694  (not concave)
Iteration 91:  log likelihood =  2.3661694  (not concave)
Iteration 92:  log likelihood =  2.3661694  (not concave)
Iteration 93:  log likelihood =  2.3661694  (not concave)
Iteration 94:  log likelihood =  2.3661694  (not concave)
(switching optimization to BFGS)
Iteration 95:  log likelihood =  2.3661694
```

```
ARIMA regression

Sample:  2012w19 - 2013w36                      Number of obs      =        70
                                                Wald chi2(11)      =   8926.00
Log likelihood =  2.366169                      Prob > chi2        =    0.0000

------------------------------------------------------------------------------
             |                 OPG
      D.lnHi |      Coef.   Std. Err.      z    P>|z|     [95% Conf. Interval]
-------------+----------------------------------------------------------------
lnHi         |
       L7DWP |
         D1. |   .0297894   .0092286     3.23   0.001     .0117017    .0478771
             |
       L7LST |
         D1. |   .0927742   .0230971     4.02   0.000     .0475048    .1380437
             |
      L5NDVI |
         D1. |   1.031031   .6701947     1.54   0.124     -.282526    2.344589
             |
      L7NDVI |
         D1. |   1.410961   .6441065     2.19   0.028     .1485356    2.673387
             |
       _cons |  -.0094847   .0058952    -1.61   0.108    -.0210392    .0020697
-------------+----------------------------------------------------------------
ARMA         |
          ar |
         L1. |  -1.190511   .1548594    -7.69   0.000     -1.49403   -.8869921
         L2. |  -.0864805   .2843526    -0.30   0.761    -.6438014    .4708404
         L3. |   .0146235   .2440309     0.06   0.952    -.4636682    .4929152
         L4. |   .3262406   .2933412     1.11   0.266    -.2486975    .9011787
         L5. |   .4663633   .1622761     2.87   0.004     .1483079    .7844186
             |
          ma |
         L1. |   .7111666   .1972891     3.60   0.000     .3244871    1.097846
         L2. |  -.2699879          .        .       .            .           .
         L3. |   .2700154   .1925918     1.40   0.161    -.1074577    .6474884
         L4. |  -.7112077          .        .       .            .           .
         L5. |  -1.000035          .        .       .            .           .
-------------+----------------------------------------------------------------
      /sigma |   .2157641   .0224006     9.63   0.000     .1718597    .2596685
------------------------------------------------------------------------------
Note: The test of the variance against zero is one sided, and the two-sided confidence interval
is truncated at zero.

.
. estat ic

-----------------------------------------------------------------------------
       Model |    Obs    ll(null)   ll(model)     df          AIC         BIC
-------------+---------------------------------------------------------------
           . |     70           .    2.366169     13     21.26766     50.4981
-----------------------------------------------------------------------------
               Note:  N=Obs used in calculating BIC; see [R] BIC note

.
. estimates store M38

.
.
.
. arima lnHi L7DWP L7LST L5NDVI L7NDVI if tin(2012w19, 2013w36), arima(4,1,5)

(setting optimization to BHHH)
Iteration 0:   log likelihood = -7.5377054
Iteration 1:   log likelihood = -5.7167335
Iteration 2:   log likelihood = -5.6998595  (backed up)
Iteration 3:   log likelihood = -5.6955487  (backed up)
Iteration 4:   log likelihood = -5.6952544  (backed up)
(switching optimization to BFGS)
```

```
Iteration 5:   log likelihood = -5.6858519  (backed up)
Iteration 6:   log likelihood = -2.5369203
Iteration 7:   log likelihood = -1.9739519
Iteration 8:   log likelihood = -.86960564
Iteration 9:   log likelihood =  -.6129126
Iteration 10:  log likelihood = -.32858821
Iteration 11:  log likelihood = -.08267033
Iteration 12:  log likelihood = -.02310451
Iteration 13:  log likelihood =  .08795086
Iteration 14:  log likelihood =  .16085068
(switching optimization to BHHH)
Iteration 15:  log likelihood =  .17231189
Iteration 16:  log likelihood =  .17232923  (backed up)
Iteration 17:  log likelihood =  .17233221  (backed up)
Iteration 18:  log likelihood =  .17233225  (backed up)
Iteration 19:  log likelihood =  .17233226  (backed up)
(switching optimization to BFGS)
Iteration 20:  log likelihood =  .17233228  (backed up)
Iteration 21:  log likelihood =  .17279008  (backed up)
Iteration 22:  log likelihood =  .17707584
Iteration 23:  log likelihood =  .17883138
Iteration 24:  log likelihood =  .18054497
Iteration 25:  log likelihood =  .18118543
Iteration 26:  log likelihood =  .18143258
Iteration 27:  log likelihood =  .18152745
Iteration 28:  log likelihood =   .1817194
Iteration 29:  log likelihood =  .18173679
(switching optimization to BHHH)
Iteration 30:  log likelihood =  .18174197  (not concave)
Iteration 31:  log likelihood =  .18174206  (not concave)
Iteration 32:  log likelihood =  .18174207
Iteration 33:  log likelihood =  .18174208  (not concave)
Iteration 34:  log likelihood =  .18174208  (not concave)
(switching optimization to BFGS)
Iteration 35:  log likelihood =  .18174208
Iteration 36:  log likelihood =  .18174208  (backed up)
Iteration 37:  log likelihood =  .18174208  (backed up)
Iteration 38:  log likelihood =  .18174208  (backed up)
Iteration 39:  log likelihood =  .18174208  (backed up)
Iteration 40:  log likelihood =  .18174208  (backed up)
Iteration 41:  log likelihood =  .18174208  (backed up)
Iteration 42:  log likelihood =  .18174208  (backed up)
Iteration 43:  log likelihood =  .18174208  (backed up)
Iteration 44:  log likelihood =  .18174208  (backed up)
(switching optimization to BHHH)
Iteration 45:  log likelihood =  .18174208  (not concave)
Iteration 46:  log likelihood =  .18174208  (not concave)
Iteration 47:  log likelihood =  .18174208  (not concave)
Iteration 48:  log likelihood =  .18174209  (not concave)
Iteration 49:  log likelihood =  .18174209  (not concave)
(switching optimization to BFGS)
Iteration 50:  log likelihood =  .18174209

ARIMA regression

Sample:  2012w19 - 2013w36                      Number of obs      =        70
                                                Wald chi2(11)      =    169.46
Log likelihood =  .1817421                      Prob > chi2        =    0.0000

------------------------------------------------------------------------------
             |                 OPG
      D.lnHi |      Coef.   Std. Err.      z    P>|z|     [95% Conf. Interval]
-------------+----------------------------------------------------------------
lnHi         |
      L7DWP |
         D1. |   .0332634   .0098597     3.37   0.001     .0139386    .0525881
             |
       L7LST |
         D1. |   .0899009   .0243424     3.69   0.000     .0421907    .1376112
             |
      L5NDVI |
```

```
         D1. |   .8351238   .6194121     1.35   0.178    -.3789016    2.049149
             |
      L7NDVI |
         D1. |   1.327547   .6053621     2.19   0.028      .141059    2.514035
             |
       _cons |  -.0090746   .0060191    -1.51   0.132    -.0208718    .0027226
-------------+----------------------------------------------------------------
ARMA         |
          ar |
         L1. |  -.1792738   .5871357    -0.31   0.760    -1.330039    .9714911
         L2. |   .1817347   .2382908     0.76   0.446    -.2853066     .648776
         L3. |  -.1227614   .1783554    -0.69   0.491    -.4723315    .2268088
         L4. |   .3864101   .2046556     1.89   0.059    -.0147076    .7875277
             |
          ma |
         L1. |  -.3786442   .8603982    -0.44   0.660    -2.064994    1.307705
         L2. |   .0452942          .        .       .            .           .
         L3. |   .2697856   .4878272     0.55   0.580    -.6863382    1.225909
         L4. |  -.9968925          .        .       .            .           .
         L5. |   .0604583   .4957896     0.12   0.903    -.9112714    1.032188
-------------+----------------------------------------------------------------
      /sigma |   .2284507   .0317594     7.19   0.000     .1662034    .2906981
------------------------------------------------------------------------------
Note: The test of the variance against zero is one sided, and the two-sided confidence interval
is truncated at zero.

.
. estat ic

-----------------------------------------------------------------------------
       Model |    Obs    ll(null)   ll(model)     df          AIC         BIC
-------------+---------------------------------------------------------------
           . |     70           .    .1817421     13     25.63652    54.86695
-----------------------------------------------------------------------------
               Note:  N=Obs used in calculating BIC; see [R] BIC note

.
. estimates store M39

.
.
.
. arima lnHi L7DWP L7LST L5NDVI L7NDVI if tin(2012w19, 2013w36), arima(4,1,4)

(setting optimization to BHHH)
Iteration 0:   log likelihood = -7.3257399
Iteration 1:   log likelihood =  -4.753895
Iteration 2:   log likelihood =  -4.567625
Iteration 3:   log likelihood = -4.4877472  (backed up)
Iteration 4:   log likelihood = -4.4873649  (backed up)
(switching optimization to BFGS)
Iteration 5:   log likelihood = -4.4873088  (backed up)
Iteration 6:   log likelihood = -1.8132975
Iteration 7:   log likelihood = -.84778752
Iteration 8:   log likelihood = -.42186544
Iteration 9:   log likelihood = -.29792567
Iteration 10:  log likelihood = -.14047295
Iteration 11:  log likelihood = -.08906573
Iteration 12:  log likelihood =  .08832849
Iteration 13:  log likelihood =  .09951972
Iteration 14:  log likelihood =  .13238818
(switching optimization to BHHH)
Iteration 15:  log likelihood =  .13996654
Iteration 16:  log likelihood =  .13996778  (backed up)
Iteration 17:  log likelihood =  .13996822  (backed up)
Iteration 18:  log likelihood =  .13996828  (backed up)
Iteration 19:  log likelihood =  .13996828  (backed up)
(switching optimization to BFGS)
Iteration 20:  log likelihood =  .13996828  (backed up)
Iteration 21:  log likelihood =  .14182455  (backed up)
Iteration 22:  log likelihood =  .14570765
```

```
Iteration 23:  log likelihood =  .14686778
Iteration 24:  log likelihood =   .1474736
Iteration 25:  log likelihood =  .14750265
Iteration 26:  log likelihood =  .14752537
Iteration 27:  log likelihood =  .14752873
Iteration 28:  log likelihood =  .14752901
Iteration 29:  log likelihood =  .14752911

ARIMA regression

Sample:  2012w19 - 2013w36                      Number of obs      =        70
                                                Wald chi2(10)      =    173.44
Log likelihood =  .1475291                      Prob > chi2        =    0.0000

------------------------------------------------------------------------------
             |                 OPG
      D.lnHi |      Coef.   Std. Err.      z    P>|z|     [95% Conf. Interval]
-------------+----------------------------------------------------------------
lnHi         |
       L7DWP |
         D1. |   .0336097   .0093779     3.58   0.000     .0152292    .0519901
             |
       L7LST |
         D1. |   .0893415   .0240812     3.71   0.000     .0421431    .1365398
             |
      L5NDVI |
         D1. |   .8242303    .579412     1.42   0.155    -.3113964    1.959857
             |
      L7NDVI |
         D1. |   1.312499   .5783868     2.27   0.023     .1788816    2.446116
             |
       _cons |  -.0090038   .0058818    -1.53   0.126     -.020532    .0025244
-------------+----------------------------------------------------------------
ARMA         |
          ar |
         L1. |  -.2352599   .2322101    -1.01   0.311    -.6903833    .2198635
         L2. |   .1668797   .2260443     0.74   0.460    -.2761591    .6099184
         L3. |  -.1087537   .1741796    -0.62   0.532    -.4501394     .232632
         L4. |   .3735496   .2093134     1.78   0.074    -.0366972    .7837964
             |
          ma |
         L1. |  -.2762705   .4355393    -0.63   0.526    -1.129912    .5773709
         L2. |  -.0267155          .        .       .            .           .
         L3. |   .3235882   .4529449     0.71   0.475    -.5641675    1.211344
         L4. |  -1.020602          .        .       .            .           .
-------------+----------------------------------------------------------------
      /sigma |   .2237878   .0300965     7.44   0.000     .1647998    .2827758
------------------------------------------------------------------------------
Note: The test of the variance against zero is one sided, and the two-sided confidence interval
is truncated at zero.

.
. estat ic

-----------------------------------------------------------------------------
       Model |    Obs    ll(null)   ll(model)     df          AIC         BIC
-------------+---------------------------------------------------------------
           . |     70           .    .1475291     12     23.70494    50.68688
-----------------------------------------------------------------------------
               Note:  N=Obs used in calculating BIC; see [R] BIC note

.
. estimates store M40

.
.
.
. estimates stats M1 M101 M102 M103 M2 M3 M4 M5 M6 M7 M8 M9 M10 M11 M12 M13 M14 M15 M16 M17 M18
M19 M20 M21 M22 M23 M24 M25 M26 M27 M28 M29 M30 M31 M32 M33 M34 M35 M36 M201 M202 M37 M38 M39
>  M40
```

Model	Obs	ll(null)	ll(model)	df	AIC	BIC
M1	70	.	-7.168514	10	34.33703	56.82198
M101	70	.	-13.42246	6	38.84492	52.33589
M102	70	.	-16.42423	5	42.84847	54.09095
M103	70	.	-11.47655	6	34.95309	48.44406
M2	70	.	-7.355228	9	32.71046	52.94691
M3	70	.	-7.716914	8	31.43383	49.42179
M4	70	.	-8.655586	7	31.31117	47.05064
M5	70	.	-10.88849	6	33.77699	47.26796
M6	70	.	-13.99401	5	37.98802	49.2305
M7	70	.	-21.10717	4	50.21433	59.20831
M8	70	.	-8.655586	7	31.31117	47.05064
M9	70	.	-7.94795	8	31.8959	49.88386
M10	70	.	-7.203828	8	30.40766	48.39562
M11	70	.	-7.725932	9	33.45186	53.68832
M12	70	.	-6.32069	8	28.64138	46.62934
M13	70	.	-2.767082	9	23.53416	43.77062
M14	70	.	-2.792328	10	25.58466	48.06961
M15	70	.	-2.670309	10	25.34062	47.82557
M16	70	.	-2.309591	11	26.61918	51.35263
M17	70	.	.468079	11	21.06384	45.79729
M18	70	.	-.8144143	12	25.62883	52.61077
M19	70	.	-1.186507	12	26.37301	53.35496
M20	70	.	1.781855	12	20.43629	47.41823
M21	70	.	-10.88849	6	33.77699	47.26796
M22	70	.	-9.779038	7	33.55808	49.29754
M23	70	.	-8.664278	7	31.32856	47.06802
M24	70	.	-9.331308	8	34.66262	52.65058
M25	70	.	-7.24422	8	30.48844	48.4764
M26	70	.	-3.587484	8	23.17497	41.16293
M27	70	.	-3.596666	8	23.19333	41.18129
M28	70	.	-3.135647	8	22.27129	40.25926
M29	70	.	-2.878773	10	25.75755	48.2425
M30	70	.	-2.954057	10	25.90811	48.39307
M31	70	.	-1.674761	10	23.34952	45.83448
M32	70	.	-.9054903	11	23.81098	48.54443
M33	70	.	-.3243799	11	22.64876	47.38221
M34	70	.	.1368329	11	21.72633	46.45978
M35	70	.	-.0956328	12	24.19127	51.17321
M36	70	.	2.144636	13	21.71073	50.94117
M201	70	.	2.74636	14	22.50728	53.98621
M202	70	.	6.712289	14	14.57542	46.05436
M37	70	.	7.329185	14	13.34163	44.82056
M38	70	.	2.366169	13	21.26766	50.4981
M39	70	.	.1817421	13	25.63652	54.86695
M40	70	.	.1475291	12	23.70494	50.68688

Note: N=Obs used in calculating BIC; see [R] BIC note

```
.
.
.
. ** We see here that M37 and M202 are the best models in terms of AIC. Therefore we use these
two to create

.
. ** predictions for the study period, now involving the fitting period -(2012w19 - 2013w36) as
well as the

.
. ** evaluation period (2011w19-2012w18). Then we recalculate the logtransformation, creating
numbers in actual predicted incidence.

.
.
.
. arima lnHi L7DWP L7LST L5NDVI L7NDVI, arima(6,1,5)

(setting optimization to BHHH)
```

```
Iteration 0:   log likelihood = -22.298679
Iteration 1:   log likelihood = -18.887532
Iteration 2:   log likelihood = -18.691433
Iteration 3:   log likelihood = -18.284163
Iteration 4:   log likelihood = -18.270472  (backed up)
(switching optimization to BFGS)
Iteration 5:   log likelihood = -18.255182  (backed up)
Iteration 6:   log likelihood = -15.923284
Iteration 7:   log likelihood = -15.853381
Iteration 8:   log likelihood = -15.460629
Iteration 9:   log likelihood = -14.689023
Iteration 10:  log likelihood = -14.587843
Iteration 11:  log likelihood =  -13.90904
Iteration 12:  log likelihood = -13.417209
Iteration 13:  log likelihood = -13.048731
Iteration 14:  log likelihood = -12.862278
(switching optimization to BHHH)
Iteration 15:  log likelihood = -12.562738
Iteration 16:  log likelihood = -12.540436
Iteration 17:  log likelihood = -12.534138  (backed up)
Iteration 18:  log likelihood = -12.512145
Iteration 19:  log likelihood = -12.505572  (backed up)
(switching optimization to BFGS)
Iteration 20:  log likelihood = -12.505435  (backed up)
Iteration 21:  log likelihood = -12.468327
Iteration 22:  log likelihood = -12.071319
Iteration 23:  log likelihood = -11.569511
Iteration 24:  log likelihood = -11.433567
Iteration 25:  log likelihood = -11.239107
Iteration 26:  log likelihood = -11.190798
Iteration 27:  log likelihood = -10.799541
Iteration 28:  log likelihood = -10.645571
Iteration 29:  log likelihood = -10.527881
(switching optimization to BHHH)
Iteration 30:  log likelihood = -10.475649
Iteration 31:  log likelihood = -10.467962  (backed up)
Iteration 32:  log likelihood = -10.467136  (backed up)
Iteration 33:  log likelihood =  -10.46706  (backed up)
Iteration 34:  log likelihood = -10.466921  (backed up)
(switching optimization to BFGS)
Iteration 35:  log likelihood =  -10.46692  (backed up)
Iteration 36:  log likelihood =  -10.46648  (backed up)
Iteration 37:  log likelihood = -10.463073
Iteration 38:  log likelihood = -10.449686
Iteration 39:  log likelihood = -10.442555
Iteration 40:  log likelihood = -10.440498
Iteration 41:  log likelihood = -10.439305
Iteration 42:  log likelihood = -10.439111
Iteration 43:  log likelihood = -10.439062
Iteration 44:  log likelihood = -10.439049
(switching optimization to BHHH)
Iteration 45:  log likelihood = -10.439045  (not concave)
Iteration 46:  log likelihood = -10.439045  (not concave)
Iteration 47:  log likelihood = -10.439045  (not concave)
Iteration 48:  log likelihood = -10.439045  (not concave)
Iteration 49:  log likelihood = -10.439045  (not concave)
(switching optimization to BFGS)
Iteration 50:  log likelihood = -10.439045

ARIMA regression

Sample:  2011w27 - 2013w36                      Number of obs      =       114
                                                Wald chi2(13)      =    448.71
Log likelihood = -10.43905                      Prob > chi2        =    0.0000

------------------------------------------------------------------------------
             |                 OPG
      D.lnHi |      Coef.   Std. Err.      z    P>|z|     [95% Conf. Interval]
-------------+----------------------------------------------------------------
lnHi         |
       L7DWP |
```

```
         D1. |   .0257025   .0111891     2.30   0.022     .0037722    .0476329
             |
       L7LST |
         D1. |   .1152267   .0146107     7.89   0.000     .0865903    .1438632
             |
      L5NDVI |
         D1. |   .6947368   .5566177     1.25   0.212    -.3962139    1.785688
             |
      L7NDVI |
         D1. |   1.537456   .4923495     3.12   0.002     .5724683    2.502443
             |
       _cons |  -.0083395   .0006945   -12.01   0.000    -.0097008   -.0069783
-------------+----------------------------------------------------------------
ARMA         |
          ar |
         L1. |   .2909708   .1402287     2.07   0.038     .0161276     .565814
         L2. |   .2988342   .1578226     1.89   0.058    -.0104925    .6081609
         L3. |    .298608   .1275222     2.34   0.019     .0486692    .5485469
         L4. |    .561064   .1195376     4.69   0.000     .3267745    .7953534
         L5. |  -.5501359   .1226556    -4.49   0.000    -.7905364   -.3097354
         L6. |  -.2597369   .1335631    -1.94   0.052    -.5215157    .0020419
             |
          ma |
         L1. |  -.8485374          .        .       .            .           .
         L2. |  -.1277489   .0961706    -1.33   0.184    -.3162398     .060742
         L3. |  -.2187091          .        .       .            .           .
         L4. |  -.7745337   .2491407    -3.11   0.002     -1.26284   -.2862268
         L5. |   .9737784   .2796871     3.48   0.000     .4256018    1.521955
-------------+----------------------------------------------------------------
      /sigma |   .2489817   .0307607     8.09   0.000     .1886919    .3092716
------------------------------------------------------------------------------
Note: The test of the variance against zero is one sided, and the two-sided confidence interval
is truncated at zero.

.
. predict M37, y
(14 missing values generated)

.
. gen ARIMAHIGHM37=exp(M37)
(14 missing values generated)

.
.
.
. arima lnHi L7DWP L7LST L7NDVI, arima(6,1,5)

(setting optimization to BHHH)
Iteration 0:   log likelihood = -23.607841
Iteration 1:   log likelihood = -20.761276
Iteration 2:   log likelihood = -18.646794
Iteration 3:   log likelihood = -18.339528
Iteration 4:   log likelihood = -17.842438
(switching optimization to BFGS)
Iteration 5:   log likelihood = -17.818927  (backed up)
Iteration 6:   log likelihood = -16.086962
Iteration 7:   log likelihood = -15.512626
Iteration 8:   log likelihood = -15.262353
Iteration 9:   log likelihood = -14.712251
Iteration 10:  log likelihood = -14.628691
Iteration 11:  log likelihood = -14.507857
Iteration 12:  log likelihood = -14.440531
Iteration 13:  log likelihood = -14.438149
Iteration 14:  log likelihood = -14.408116
(switching optimization to BHHH)
Iteration 15:  log likelihood = -14.233257
Iteration 16:  log likelihood = -14.218202  (backed up)
Iteration 17:  log likelihood = -14.210509  (backed up)
Iteration 18:  log likelihood = -14.210361  (backed up)
Iteration 19:  log likelihood =  -14.21034  (backed up)
(switching optimization to BFGS)
```

```
Iteration 20:  log likelihood = -14.210334  (backed up)
Iteration 21:  log likelihood = -14.155911
Iteration 22:  log likelihood =  -14.04352
Iteration 23:  log likelihood =  -13.88572
Iteration 24:  log likelihood =  -13.39612
Iteration 25:  log likelihood = -12.963994
Iteration 26:  log likelihood = -12.616855
Iteration 27:  log likelihood = -12.118749
Iteration 28:  log likelihood =  -11.80311
Iteration 29:  log likelihood =  -11.64575
(switching optimization to BHHH)
Iteration 30:  log likelihood = -11.538191
Iteration 31:  log likelihood = -11.537383  (backed up)
Iteration 32:  log likelihood = -11.537136  (backed up)
Iteration 33:  log likelihood =  -11.53707  (backed up)
Iteration 34:  log likelihood =  -11.53707  (backed up)
(switching optimization to BFGS)
Iteration 35:  log likelihood =  -11.53707  (backed up)
Iteration 36:  log likelihood = -11.534729
Iteration 37:  log likelihood = -11.526747
Iteration 38:  log likelihood = -11.523731
Iteration 39:  log likelihood = -11.515017
Iteration 40:  log likelihood = -11.511784
Iteration 41:  log likelihood =  -11.51116
Iteration 42:  log likelihood = -11.511021
Iteration 43:  log likelihood = -11.510877
Iteration 44:  log likelihood =  -11.51086
(switching optimization to BHHH)
Iteration 45:  log likelihood = -11.510857  (not concave)
Iteration 46:  log likelihood = -11.510857  (not concave)
Iteration 47:  log likelihood = -11.510857  (not concave)
Iteration 48:  log likelihood = -11.510857  (not concave)
Iteration 49:  log likelihood = -11.510857  (not concave)
(switching optimization to BFGS)
Iteration 50:  log likelihood = -11.510857
Iteration 51:  log likelihood = -11.510857  (backed up)
Iteration 52:  log likelihood = -11.510857  (backed up)
Iteration 53:  log likelihood = -11.510857  (backed up)
Iteration 54:  log likelihood = -11.510857  (backed up)
Iteration 55:  log likelihood = -11.510857  (backed up)
Iteration 56:  log likelihood = -11.510857  (backed up)
Iteration 57:  log likelihood = -11.510857  (backed up)
Iteration 58:  log likelihood = -11.510857  (backed up)
Iteration 59:  log likelihood = -11.510857  (backed up)
(switching optimization to BHHH)
Iteration 60:  log likelihood = -11.510857  (not concave)
Iteration 61:  log likelihood = -11.510857  (not concave)
Iteration 62:  log likelihood = -11.510857  (not concave)
Iteration 63:  log likelihood = -11.510857  (not concave)
Iteration 64:  log likelihood = -11.510857  (not concave)
(switching optimization to BFGS)
Iteration 65:  log likelihood = -11.510857

ARIMA regression

Sample:  2011w27 - 2013w36                      Number of obs      =       114
                                                Wald chi2(12)      =   7745.02
Log likelihood = -11.51086                      Prob > chi2        =    0.0000

------------------------------------------------------------------------------
             |                 OPG
      D.lnHi |      Coef.   Std. Err.      z    P>|z|     [95% Conf. Interval]
-------------+----------------------------------------------------------------
lnHi         |
       L7DWP |
         D1. |   .0298873   .0096808     3.09   0.002     .0109132    .0488614
             |
       L7LST |
         D1. |   .1175246   .0145969     8.05   0.000     .0889152     .146134
             |
      L7NDVI |
```

```
         D1. |   1.928777    .392817     4.91   0.000     1.15887    2.698684
             |
       _cons |  -.0084066   .0006988   -12.03   0.000   -.0097763   -.007037
-------------+----------------------------------------------------------------
ARMA         |
          ar |
         L1. |   .3213923   .1426251     2.25   0.024     .0418522    .6009324
         L2. |   .3060204    .159101     1.92   0.054    -.0058117    .6178526
         L3. |   .2884556   .1298891     2.22   0.026     .0338777    .5430336
         L4. |   .5146181   .1334147     3.86   0.000     .2531301    .7761061
         L5. |   -.553195   .1309212    -4.23   0.000    -.8097958   -.2965942
         L6. |  -.2160199   .1352549    -1.60   0.110    -.4811146    .0490747
             |
          ma |
         L1. |  -.8612027   .3005348    -2.87   0.004     -1.45024   -.2721653
         L2. |  -.1114014   .0766365    -1.45   0.146    -.2616063    .0388034
         L3. |  -.2169854          .        .       .            .           .
         L4. |  -.7756819   .3081842    -2.52   0.012    -1.379712   -.1716519
         L5. |   .9697876          .        .       .            .           .
-------------+----------------------------------------------------------------
      /sigma |   .2513475   .0204828    12.27   0.000     .2112018    .2914931
------------------------------------------------------------------------------
Note: The test of the variance against zero is one sided, and the two-sided confidence interval
is truncated at zero.

.
. predict M202, y
(14 missing values generated)

.
. gen ARIMAHIGHM202=exp(M202)
(14 missing values generated)

.
.
.
. ** To graph these two with the actual incidence we write:

.
.
.
. tsline Hi ARIMAHIGHM202 ARIMAHIGHM37

.
.
.
. ** To analyze the residuals of the model, we use Ljung Box Q-test to see whether there is
autocorrelation in the residuals, then indicating missing

.
. *variables/missing model fit, or if these are represented by random white noise.

.
.
.
. arima lnHi L7DWP L7LST L5NDVI L7NDVI, arima(6,1,5)

(setting optimization to BHHH)
Iteration 0:   log likelihood = -22.298679
Iteration 1:   log likelihood = -18.887532
Iteration 2:   log likelihood = -18.691433
Iteration 3:   log likelihood = -18.284163
Iteration 4:   log likelihood = -18.270472  (backed up)
(switching optimization to BFGS)
Iteration 5:   log likelihood = -18.255182  (backed up)
Iteration 6:   log likelihood = -15.923284
Iteration 7:   log likelihood = -15.853381
Iteration 8:   log likelihood = -15.460629
Iteration 9:   log likelihood = -14.689023
Iteration 10:  log likelihood = -14.587843
Iteration 11:  log likelihood =  -13.90904
```

```
Iteration 12:  log likelihood = -13.417209
Iteration 13:  log likelihood = -13.048731
Iteration 14:  log likelihood = -12.862278
(switching optimization to BHHH)
Iteration 15:  log likelihood = -12.562738
Iteration 16:  log likelihood = -12.540436
Iteration 17:  log likelihood = -12.534138  (backed up)
Iteration 18:  log likelihood = -12.512145
Iteration 19:  log likelihood = -12.505572  (backed up)
(switching optimization to BFGS)
Iteration 20:  log likelihood = -12.505435  (backed up)
Iteration 21:  log likelihood = -12.468327
Iteration 22:  log likelihood = -12.071319
Iteration 23:  log likelihood = -11.569511
Iteration 24:  log likelihood = -11.433567
Iteration 25:  log likelihood = -11.239107
Iteration 26:  log likelihood = -11.190798
Iteration 27:  log likelihood = -10.799541
Iteration 28:  log likelihood = -10.645571
Iteration 29:  log likelihood = -10.527881
(switching optimization to BHHH)
Iteration 30:  log likelihood = -10.475649
Iteration 31:  log likelihood = -10.467962  (backed up)
Iteration 32:  log likelihood = -10.467136  (backed up)
Iteration 33:  log likelihood =  -10.46706  (backed up)
Iteration 34:  log likelihood = -10.466921  (backed up)
(switching optimization to BFGS)
Iteration 35:  log likelihood =  -10.46692  (backed up)
Iteration 36:  log likelihood =  -10.46648  (backed up)
Iteration 37:  log likelihood = -10.463073
Iteration 38:  log likelihood = -10.449686
Iteration 39:  log likelihood = -10.442555
Iteration 40:  log likelihood = -10.440498
Iteration 41:  log likelihood = -10.439305
Iteration 42:  log likelihood = -10.439111
Iteration 43:  log likelihood = -10.439062
Iteration 44:  log likelihood = -10.439049
(switching optimization to BHHH)
Iteration 45:  log likelihood = -10.439045  (not concave)
Iteration 46:  log likelihood = -10.439045  (not concave)
Iteration 47:  log likelihood = -10.439045  (not concave)
Iteration 48:  log likelihood = -10.439045  (not concave)
Iteration 49:  log likelihood = -10.439045  (not concave)
(switching optimization to BFGS)
Iteration 50:  log likelihood = -10.439045

ARIMA regression

Sample:  2011w27 - 2013w36                      Number of obs      =       114
                                                Wald chi2(13)      =    448.71
Log likelihood = -10.43905                      Prob > chi2        =    0.0000

------------------------------------------------------------------------------
             |                 OPG
      D.lnHi |      Coef.   Std. Err.      z    P>|z|     [95% Conf. Interval]
-------------+----------------------------------------------------------------
lnHi         |
       L7DWP |
         D1. |   .0257025   .0111891     2.30   0.022     .0037722    .0476329
             |
       L7LST |
         D1. |   .1152267   .0146107     7.89   0.000     .0865903    .1438632
             |
      L5NDVI |
         D1. |   .6947368   .5566177     1.25   0.212    -.3962139    1.785688
             |
      L7NDVI |
         D1. |   1.537456   .4923495     3.12   0.002     .5724683    2.502443
             |
       _cons |  -.0083395   .0006945   -12.01   0.000    -.0097008   -.0069783
-------------+----------------------------------------------------------------
```

```
ARMA         |
          ar |
         L1. |   .2909708   .1402287     2.07   0.038     .0161276     .565814
         L2. |   .2988342   .1578226     1.89   0.058    -.0104925    .6081609
         L3. |    .298608   .1275222     2.34   0.019     .0486692    .5485469
         L4. |    .561064   .1195376     4.69   0.000     .3267745    .7953534
         L5. |  -.5501359   .1226556    -4.49   0.000    -.7905364   -.3097354
         L6. |  -.2597369   .1335631    -1.94   0.052    -.5215157    .0020419
             |
          ma |
         L1. |  -.8485374          .        .       .            .           .
         L2. |  -.1277489   .0961706    -1.33   0.184    -.3162398     .060742
         L3. |  -.2187091          .        .       .            .           .
         L4. |  -.7745337   .2491407    -3.11   0.002     -1.26284   -.2862268
         L5. |   .9737784   .2796871     3.48   0.000     .4256018    1.521955
-------------+----------------------------------------------------------------
      /sigma |   .2489817   .0307607     8.09   0.000     .1886919    .3092716
------------------------------------------------------------------------------
Note: The test of the variance against zero is one sided, and the two-sided confidence interval
is truncated at zero.

.
. predict res, r
(15 missing values generated)

.
. wntestq res

Portmanteau test for white noise
---------------------------------------
 Portmanteau (Q) statistic =    23.9975
 Prob > chi2(40)           =     0.9787

.
.
.
. ** No autocorrelation is seen in the residuals of the models.

.
.
.
. ** Now, we also want to see what the mean average prediction error is for the evaluation
period of the best fit model.

.
. ** This is done by taking ARIMAHIGHM37 minus actual cases, then dividing this with the actual
cases. All is done as time series,

.
. ** then we divide this by (100/no of observations in the evaluation period) and multiplicate
with the sum of the residuals.

.
.
.
.
.
. gen MPEhi=(ARIMAHIGHM37-Hi)/ Hi if tin(2011w19, 2012w18)
(85 missing values generated)

.
. sum MPEhi

    Variable |       Obs        Mean    Std. Dev.       Min        Max
-------------+--------------------------------------------------------
       MPEhi |        44      .06368    .3500036  -.6812162   1.390622

.
. display r(sum)
2.8019194
```

```
.
. display = (100/(44))* 2.8019194
6.3679986

.
.
.
.
.
. ** Now, we see that the ARIMAHIGHM37 is the best model regarding AIC and has a good MAE for
the evaluation period as well as looks good visually.

.
. ** We use this model to create a forecast, this is done by:

.
.
.
. arima lnHi L7DWP L7LST L5NDVI L7NDVI, arima(6,1,5)

(setting optimization to BHHH)
Iteration 0:   log likelihood = -22.298679
Iteration 1:   log likelihood = -18.887532
Iteration 2:   log likelihood = -18.691433
Iteration 3:   log likelihood = -18.284163
Iteration 4:   log likelihood = -18.270472  (backed up)
(switching optimization to BFGS)
Iteration 5:   log likelihood = -18.255182  (backed up)
Iteration 6:   log likelihood = -15.923284
Iteration 7:   log likelihood = -15.853381
Iteration 8:   log likelihood = -15.460629
Iteration 9:   log likelihood = -14.689023
Iteration 10:  log likelihood = -14.587843
Iteration 11:  log likelihood =  -13.90904
Iteration 12:  log likelihood = -13.417209
Iteration 13:  log likelihood = -13.048731
Iteration 14:  log likelihood = -12.862278
(switching optimization to BHHH)
Iteration 15:  log likelihood = -12.562738
Iteration 16:  log likelihood = -12.540436
Iteration 17:  log likelihood = -12.534138  (backed up)
Iteration 18:  log likelihood = -12.512145
Iteration 19:  log likelihood = -12.505572  (backed up)
(switching optimization to BFGS)
Iteration 20:  log likelihood = -12.505435  (backed up)
Iteration 21:  log likelihood = -12.468327
Iteration 22:  log likelihood = -12.071319
Iteration 23:  log likelihood = -11.569511
Iteration 24:  log likelihood = -11.433567
Iteration 25:  log likelihood = -11.239107
Iteration 26:  log likelihood = -11.190798
Iteration 27:  log likelihood = -10.799541
Iteration 28:  log likelihood = -10.645571
Iteration 29:  log likelihood = -10.527881
(switching optimization to BHHH)
Iteration 30:  log likelihood = -10.475649
Iteration 31:  log likelihood = -10.467962  (backed up)
Iteration 32:  log likelihood = -10.467136  (backed up)
Iteration 33:  log likelihood =  -10.46706  (backed up)
Iteration 34:  log likelihood = -10.466921  (backed up)
(switching optimization to BFGS)
Iteration 35:  log likelihood =  -10.46692  (backed up)
Iteration 36:  log likelihood =  -10.46648  (backed up)
Iteration 37:  log likelihood = -10.463073
Iteration 38:  log likelihood = -10.449686
Iteration 39:  log likelihood = -10.442555
Iteration 40:  log likelihood = -10.440498
Iteration 41:  log likelihood = -10.439305
Iteration 42:  log likelihood = -10.439111
Iteration 43:  log likelihood = -10.439062
Iteration 44:  log likelihood = -10.439049
```

```
(switching optimization to BHHH)
Iteration 45:  log likelihood = -10.439045  (not concave)
Iteration 46:  log likelihood = -10.439045  (not concave)
Iteration 47:  log likelihood = -10.439045  (not concave)
Iteration 48:  log likelihood = -10.439045  (not concave)
Iteration 49:  log likelihood = -10.439045  (not concave)
(switching optimization to BFGS)
Iteration 50:  log likelihood = -10.439045

ARIMA regression

Sample:  2011w27 - 2013w36                      Number of obs      =       114
                                                Wald chi2(13)      =    448.71
Log likelihood = -10.43905                      Prob > chi2        =    0.0000

------------------------------------------------------------------------------
             |                 OPG
      D.lnHi |      Coef.   Std. Err.      z    P>|z|     [95% Conf. Interval]
-------------+----------------------------------------------------------------
lnHi         |
       L7DWP |
         D1. |   .0257025   .0111891     2.30   0.022     .0037722    .0476329
             |
       L7LST |
         D1. |   .1152267   .0146107     7.89   0.000     .0865903    .1438632
             |
      L5NDVI |
         D1. |   .6947368   .5566177     1.25   0.212    -.3962139    1.785688
             |
      L7NDVI |
         D1. |   1.537456   .4923495     3.12   0.002     .5724683    2.502443
             |
       _cons |  -.0083395   .0006945   -12.01   0.000    -.0097008   -.0069783
-------------+----------------------------------------------------------------
ARMA         |
          ar |
         L1. |   .2909708   .1402287     2.07   0.038     .0161276     .565814
         L2. |   .2988342   .1578226     1.89   0.058    -.0104925    .6081609
         L3. |    .298608   .1275222     2.34   0.019     .0486692    .5485469
         L4. |    .561064   .1195376     4.69   0.000     .3267745    .7953534
         L5. |  -.5501359   .1226556    -4.49   0.000    -.7905364   -.3097354
         L6. |  -.2597369   .1335631    -1.94   0.052    -.5215157    .0020419
             |
          ma |
         L1. |  -.8485374          .        .       .            .           .
         L2. |  -.1277489   .0961706    -1.33   0.184    -.3162398     .060742
         L3. |  -.2187091          .        .       .            .           .
         L4. |  -.7745337   .2491407    -3.11   0.002     -1.26284   -.2862268
         L5. |   .9737784   .2796871     3.48   0.000     .4256018    1.521955
-------------+----------------------------------------------------------------
      /sigma |   .2489817   .0307607     8.09   0.000     .1886919    .3092716
------------------------------------------------------------------------------
Note: The test of the variance against zero is one sided, and the two-sided confidence interval
is truncated at zero.

.
. predict M37fcast, dynamic(tw(2013w37)) y
(8 missing values generated)

.
.
.
. gen Forecast=exp(M37fcast)
(8 missing values generated)

.
.
.
. * In our study, we choose to use 4weekly forecasts. Therefore we first calculate the residuals
for each week comparing forecast with the actual incidence just
```

```
.
. * as the MAE was calculated for the evaluation period. See example below. However, this was
done in Excel and is not included here more than by example.

.
.
.
. * gen MPEForecast=(ARIMAHIGHM37-Hi)/ Hi if tin(2013w37, 2013w41)

.
. * sum MPEhi

.
. * display r(sum)

.
. * display = (100/(4))* r(sum)

. log close
      name:  <unnamed>
       log:  C:\Users\David\Google Drive\Zambia - ISEX T10\Data Monze + Choma\FINAL MODELS -
GRAPHS AND DOFILES\Log-file - exemplifying the process using High Zone.smcl
  log type:  smcl
 closed on:  27 Dec 2013, 12:42:18
-------------------------------------------------------------------------------------------------
----------------------------------------------------------------------------------------------
```
