# Supplementary material for: Remotely-sensed, nocturnal, dew point correlates with malaria transmission in Southern Province, Zambia: a time-series study
Source: Malar J. 2014 Jun 13;13:231. doi: 10.1186/1475-2875-13-231 (PMC4078093; doi:10.1186/1475-2875-13-231)
Supplement: Additional file 2 — Graphs of environment variables: graphs explaining temporal variations of environmental variables studied within zonal comparisons. [file 1475-2875-13-231-S2.docx]

Additional file 2

*Remotely sensed nocturnal dew point correlates with malaria transmission in Southern Province, Zambia: a time-series study.*

*Graphs of environmental variables studied:*

***Figure 4 - Zonal Nocturnal Dew Point Comparison****: Graph Showing Nocturnal Dew Point average measurements for the High, Medium and Low transmission zones for the study period 2011 w19 – 2013 w41.*

***Figure 5 – Zonal Nocturnal Land Surface Temperature Comparison:*** *Graph showing nocturnal land surface temperature average measurements for the High, Medium and Low transmission zones for the study period 2011 w19 – 2013 w41.*

***Figure 6 – Zonal Normalized Differentiation Vegetation Index Comparison:*** *Graph showing normalized differentiation vegetation index (NDVI) average measurements for the High, Medium and Low Transmission zones for the study period 2011 w19 – 2013 w41.*

***Figure 7 – Zonal Rainfall Comparison:*** *Graph showing rainfall amount estimates for the High, Medium and Low Transmission zones from May 2011 to July 2013.*

***Figure 8 – Zonal Incidence Comparison****: Graphs of weekly incidence of the three zones created from the RHCs we have been looking at in Southern Province with reporting starting between 2011w19 and 2012w19 and then continuing up until 2013w36. The zonal division is based on their transmission May-November. The seasonal pattern throughout SP is here seen with December-April as the high season and May-November as the low.*
